# Effectiveness and safety of 7-day high-dose primaquine and single-dose tafenoquine versus 14-day low-dose primaquine in patients with *Plasmodium vivax* malaria (EFFORT): a multicentre, open-label, randomised, controlled, superiority trial

**DOI:** 10.1016/S1473-3099(25)00729-7

**Published:** 2026-06

**Authors:** Tamiru Shibiru Degaga, Ayodhia Pitaloka Pasaribu, Rupam Tripura, Najia Ghanchi, Megha Rajasekhar, Bipin Adhikari, Benedikt Ley, Samuel Alemu Bamboro, Fareeha Abdul Jabbar, Nurfadhilah Hasibuan, Tedla Teferi Tego, Shane Zehra, Bushra Qurashi, Hellen Mnjala, Grant Lee, Peixuan Li, Abdul Momin Kazi, Widaya Safitri, Yulita Yulita, Dedi Sahat Parningotan Siagian, Deni Syahputra, Halik Hadi, Taj Muhammad, Amna Ibrahim, Nasreen Syed, Khuda Dost, Agatha Mia Puspitrasari, Pinkan Pertiwi Kariodimedjo, Anjana Rai, Angela Rumaseb, Edwin Sutanto, Mom Ean, Asia Khan, Meas Sokha, Robert James Commons, Sophie Weston, Rintis Noviyanti, Tom Peto, James J Callery, Umair Ali, Tariq Mehmood, Arjen Dondorp, Angela Devine, Ery Setiawan, Muthoni Mwaura, Sarah Cassidy-Seyoum, Rodas Temesgen, Dagimawie Tadesse Abate, Eyerusalem Beyene Erjabo, Geremew Gashaw Gessa, Fitsum Getahun Kiros, Muhammad Imran Usmani, Ali Raza, Adugna Woyessa, Asrat Hailu, Julie A Simpson, Amalia Karahalios, Mohammad Asim Beg, Lorenz von Seidlein, Lek Dysoley, Sarah Auburn, Ric N Price, Kamala Thriemer

**Affiliations:** a Global and Tropical Health Division, Menzies School of Health Research and Charles Darwin University, Darwin, NT, Australia; b College of Medicine & Health Sciences, Arba Minch University, Arba Minch, Ethiopia; c Department of Pediatrics, Medical Faculty, Universitas Sumatera Utara, Medan, Indonesia; d Yayasan Penguatan Kesehatan Masyarakat Tridarma (YPKMT; Tridarma Healthcare Empowerment Foundation), Medan, Indonesia; e Mahidol Oxford Tropical Medicine Research Unit, Faculty of Tropical Medicine, Mahidol University, Bangkok, Thailand; f Centre for Tropical Medicine and Global Health, Nuffield Department of Clinical Medicine, University of Oxford, Oxford, UK; g Department of Pathology and Laboratory Medicine, Aga Khan University, Karachi, Pakistan; h Centre for Epidemiology and Biostatistics, Melbourne School of Population and Global Health, University of Melbourne, Melbourne, VIC, Australia; i Arba Minch General Hospital, Arba Minch, Ethiopia; j Methods and Implementation Support for Clinical Health (MISCH) Research Hub, Faculty of Medicine, Dentistry and Health Sciences, University of Melbourne, Melbourne, VIC, Australia; k Department of Paediatrics, Aga Khan University, Karachi, Pakistan; l Batubara District Health Office, North Sumatera, Indonesia; m North Sumatera Provincial Health Office, North Sumatera, Indonesia; n Clinical Trial Unit, Aha Khan University, Karachi, Pakistan; o Exeins Health Initiative, Jakarta, Indonesia; p Eijkman Research Centre for Molecular Biology, National Research and Innovation, Jakarta, Indonesia; q Centre for Health Policy, Melbourne School of Population and Global Health, University of Melbourne, Melbourne, VIC, Australia; r Department of Health Ethics and Society, Care and Public Health Research Institute (CAPHRI), Maastricht University, Maastricht, Netherlands; s Ethiopian Public Health Institute, Addis Abeba, Ethiopia; t Addis Abeba University, Addis Abeba, Ethiopia; u National Center for Parasitology, Entomology and Malaria Control, Phnom Penh, Cambodia; v National Institute of Public Health, School of Public Health, Phnom Penh, Cambodia

## Abstract

**Background:**

Shorter courses of primaquine and single-dose tafenoquine have potential to improve the prevention of recurrent *Plasmodium vivax* infections, but there are few data on their comparative effectiveness when provided unsupervised. We aimed to assess the effectiveness and safety of these new treatment options.

**Methods:**

We conducted a multicentre, open-label, randomised, controlled, superiority trial in Ethiopia, Pakistan, Indonesia, and Cambodia. Adult patients (aged ≥18 years, or aged ≥16 years in Indonesia) with uncomplicated *P vivax* infection and glucose-6-phosphate dehydrogenase (G6PD) activity of 70% or greater were eligible for enrolment. Patients were treated with blood schizonticidal drugs (chloroquine in Ethiopia and Pakistan, dihydroartemisinin–piperaquine in Indonesia, and artesunate–pyronaridine in Cambodia) and randomly assigned (1:1:1) by an independent statistician using randomly permuted blocks of varying sizes to receive 7 days of unsupervised high-dose primaquine (total dose 7 mg/kg), single-dose tafenoquine (300 mg), or 14 days of low-dose primaquine (total dose 3·5 mg/kg). Randomisation was stratified by site. The primary endpoint, assessed in the modified intention-to-treat population (ie, patients who received allocated treatment, and were not lost to follow-up before day 15), was the cumulative incidence of any *P vivax* parasitaemia within 6 months compared between the primaquine groups, with the comparison of cumulative incidence between the tafenoquine group and the 14-day low-dose primaquine group a secondary endpoint. Key safety outcomes included the numbers of adverse and serious adverse events, including the number of gastrointestinal symptoms as well as the risk of anaemia. Safety outcomes were assessed in all patients who received any doses of study drug. The study is registered at ClinicalTrials.gov, NCT04411836, and is complete.

**Findings:**

Between April 25, 2021 and Sept 13, 2024, 4850 patients were screened for eligibility, of whom 960 patients (672 [70·0%] were male and 288 [30·0%] were female) were enrolled and randomly assigned (320 patients per group). The modified intention-to-treat population of 295 patients allocated to the 7-day high-dose primaquine group, 305 patients allocated to the tafenoquine group, and 301 patients allocated to the 14-day low-dose primaquine group were considered at risk of *P vivax* recurrence from day 15 onwards. The cumulative incidence of *P vivax* recurrence at 6 months was 13·0% (97·55% CI 9·0–18·5) in the 7-day high-dose primaquine group and 18·5% (13·8–24·6) in the 14-day low-dose primaquine group (hazard ratio [HR] 0·66 [97·55% CI 0·40–1·09], p=0·063). The corresponding incidence in the tafenoquine group was 12·6% (97·55% CI 8·8–18·0), with an HR of 0·64 (97·55% CI 0·39–1·05, p=0·041) compared with the 14-day low-dose primaquine group. Of the adverse events occurring before day 42, 24 (42·9%) of 56 were considered drug related in the 7-day high-dose primaquine group, compared with 16 (22·2%) of 72 in the tafenoquine group and 13 (34·2%) of 38 in the 14-day low-dose primaquine group. Four serious adverse events occurred, of which two (gastrointestinal symptoms in two patients in the 7-day high-dose primaquine group) were considered probably related to study drug. One study drug unrelated death occurred in the 14-day low-dose primaquine group.

**Interpretation:**

Both unsupervised 7-day high-dose primaquine and single-dose tafenoquine were well tolerated in patients with normal G6PD activity and they had a lower risk of *P vivax* recurrence compared with those in the 14-day low-dose primaquine group, albeit with uncertain magnitude. Our findings support the effectiveness and operational feasibility of shorter radical cure regimens across diverse malaria-endemic settings.

**Funding:**

National Health and Medical Research Council of Australia and Bill and Melinda Gates Foundation.

## Abstract (am)

**ረቂቅ**

**ዳራ**

ለአጭር ጊዜ የሚወሰድ ፕሪማኩዊን እና አንድ ጊዜ ብቻ የሚወሰድ የታፌኖኩዊን መድሃኒት፡ በተደጋጋሚ ጊዜ የሚከሰት የፕላዝሞዲየም ቫይቫክስ ወባ በሽታን የመከላከል የማሻሻል አቅም አላቸው፣ ሆኖም ግን ያለ ክትትል ሲሰጡ ያላቸውን አንጻራዊ ውጤታማነት በተመለከተ ያለው መረጃ ውስን ነው። የእነዚህን አዳዲስ የሕክምና አማራጮች ውጤታማነት እና ደህንነት ለመገምገም አስበናል ።

የምርምር ዘዴዎች

በኢትዮጵያ፣ ፓኪስታን፣ ኢንዶኔዥያ እና ካምቦዲያ ውስጥ ባለብዙ ማዕከል፣ ክፍት መለያ ያለው፣በምደባ እጣ የተደረገ እና ቁጥጥር የተደረገበት የበላይነት የሙከራ ጥናት አድርገናል። በዚህ ጥናት ለመካተት ብቁ የሆኑት ታካሚዎች፡ ያልተወሳሰበ ፕላዝሞዲየም ቫይቫክስ ወባ የታማሙ እና 70% ወይም ከዚያ በላይ የሆነ የግሉኮስ-6-ፎስፌት ዴሃይድሮጂኔስ(G6PD) እንቅስቃሴ ያላቸው አዋቂ ታካሚዎች እድሜያቸው 18 ዓመት እና ከዛ በላይ ወይም በኢንዶኔዥያ ከ16 ዓመት እና ከዛ በላይ ናቸዉ።

ታካሚዎቹ በደም ዉስጥ ያለዉን ወባ በሚያጠፉ መድኃኒቶች (በኢትዮጵያ እና ፓኪስታን በክሎሮኩዊን፣ በኢንዶኔዥያ በዳይሃይድሮአርቴሚሲን-ፓይፔራኩዊን እና በካምቦዲያ በአርቴሱኔት-ፓይሮናሪዲን) እና በገለልተኛ የስታትስቲክስ ባለሙያ ለየጥናት ጣቢያው በተለዋዋጭና ተቀያያሪ ብሎኮች የተዘጋጀ በግለሰብ ደረጃ በሚወጣ እጣ ምደባ (1፡1፡1 ጥምርታ) ፤ ክትትል የማይደረግበት ለሰባት ቀን የሚሰጥ ከፍተኛ መጠን ያለው ፕሪማኩዊን (ጠቅላላ መጠን 7 ሚ.ግ./በኪሎ ግራም) የሚወስዱ ምድብ፤ ወይም አንድ ጊዜ ብቻ የሚሰጥ ታፌኖኩዊን (300 ሚ.ግ) የሚወስዱ ምድብ፤ወይም ለ14 ቀናት የሚሰጥ ዝቅተኛ መጠን ያለው ፕሪማኩዊን (ጠቅላላ መጠን 3.5 ሚ.ግ. / በኪሎ ግራም) የሚወስዱ ምድብ ሆነው ተመድበዋል።

ዋናው የጥናቱ የመጨረሻ ዉጤት፣ በተሻሻለዉ የማከም ዓላማ ባለው ህዝብ የተመደበላቸውን ህክምና በሙሉ ያጠናቀቁ እንዲሁም ክትትላቸውን እስከ 15 ቀን ውስጥ ያላቋረጡ ውስጥ (modified intention-to-treat population) የተገመገመ፣ በ6 ወራት ውስጥ የማንኛውም ፕላዝሞዲየም ቫይቫክስ ወባ ድምር ክስተት ሲሆን ፕሪማኩዊንን ከወሰዱ ምድቦች መካከል እርስ በእርስ ማወዳደር ነዉ፤ የቫይቫክስ ወባ ድምር ክስተት ንጽጽር ታፌኖኩዊን የወሰዱ ምድብ እና የ14 ቀናት የሚሰጥ ዝቅተኛ መጠን ያለው ፕሪማኩዊን የወሰዱ ምድብ በሁለተኛ ደረጃ ያየነው የመጨረሻ ዉጤት ነበር ። ቁልፍ የደህንነት መለኪያ የነበሩት ቀለል ያሉና ከበዱ ያሉ የጎንዮሽ ጉዳቶች፤ የሆድ ህመምና የደም ማነስ ተጋላጭነትን ያካተተ ነበር፡፡ ቁልፍ የደህንነት መለኪያዎች በሁሉም የጥናቱን መድሐኒት በወሰዱት ላይ ተጠንቷል፡፡ ጥናቱ በ ClinicalTrials.gov (NCT04411836) ተመዝግቦና ስራው ተጠናቆ ይገኛል።

ግኝቶች

እ.ኤ.አ ከሚያዚያ 25፣ 2021 እስከ መስከረም 13፣ 2024 ባለው ጊዜ ውስጥ፤ 4850 ታካሚዎች ለጥናቱ ልየታ ተደርጎባቸው፤ 960 ታካሚዎች (672 [70·0%] ወንዶችና 288 [30·0%] ሴቶች) በእጣ በሚደረግ ምደባ በአንድ የሕክምና ቡድን 320 ታካሚዎች ተመድበዋል። በተሻሻለዉ የማከም ዓላማ ባለው፤ 295 ለሰባት ቀን የሚሰጥ ከፍተኛ መጠን ያለው ፕሪማኩዊን ምደብ ከተመደቡ፤ 305 ታካሚዎች ታፌኖኩዊን ምድብ ከተመደቡ፤ 301 ታካሚዎች የ14 ቀናት ፕሪማኩዊን መድብ ከተመደቡ የወባ ድግግሞሽ ተጋላጭነት ህክምና ከጀመሩ ከ 15 ቀናት በኋላ ጀምሮ ታይቷል፡፡

የቫይቫክስ ወባ በ6 ወራት ውስጥ የማገርሸት ያለው ድምር መጠን በ7-ቀን-ከፍተኛ-መጠን ፕሪማኩዊን ምድብ 13.0% (97.55% CI 9.0-18.5) ሲሆን፣ በ14-ቀን-ዝቅተኛ-መጠን ፕሪማኩዊን ምድብ ደግሞ 18.5% (97.55% CI 13.8-24.6) ነበር (የአደጋ ምጣኔ (HR) = 0.66፤ 97.55% CI 0.40-1.09, p=0.063)፤ ታፌኖኩዊን ምደብ ውስጥ ያለው ተጓዳኝ መጠን 12.6% (97.55% CI 8.8-18.0) ነበር፤ ከ14-ቀን-ዝቅተኛ-መጠን ፕሪማኩዊን ምድብ ጋር ሲነጻጸር (የአደጋ ምጣኔ (HR)=0.64 (97.55% CI 0.39-1.05, p=0.041) ነበር። እንዲሁም ታፌኖኩዊን በወሰዱት ታካሚዎች ዉስጥ ያለው የማገርሸት ክስተት ለ 14-ቀን ዝቅተኛ መጠን ያለው ፕሪማኩዊን ከወሳዱት ታካሚዎች ጋር ሲነጻጸር 12·6% (97.55% CI 8·8-18·0)፤ የአደጋ ምጣኔ (HR)=0·64 (97.55% CI 0·39-1·05፣ p=0·041) ነበር።

ከ42ኛዉ ቀን በፊት ከተከሰቱት የጎንዮሽ ጉዳቶች ውስጥ 24 ከ 56 ዉስጥ (42·9%) of ለ7 ቀን ከፍተኛ መጠን ያለው ፕሪማኩዊን በወሳዱት ታካሚዎች ውስጥ ሲሆን በንጽጽርም ታፌኖኩዊን በወሳዱት ታካሚዎች ውስጥ 16 ከ 72 ዉስጥ (22·2%) እና ለ14 ቀን ዝቅተኛ መጠን ያለው ፕሪማኩዊን ከወሳዱት ታካሚዎች ዉስጥ 13 ከ 38 ዉስጥ (34·2%) ነው። ይህም ከመድኃኒት ጋር የተያያዘ እንደሆነ ሪፖርት ተደርጓል። አራት ከበድ ያሉ የጎንዮሽ ጉዳቶች ተከስተዋል፤ ከነዚህም ሁለቱ ከጥናቱ መድሐኒት ጋር የተገናኘ የሆድ ህመም ለሰባት ቀን የሚሰጥ ከፍተኛ መጠን ያለው ፕሪማኩዊን የወሰዱት ምድብ ውስጥ ነው፡፡ አንድ ከጥናቱ መድሐኒት ጋር ያልተገናኘ ሞት የ14 ቀናት ፕሪማኩዊን ምድብ ውስጥ ተከስቷል፡፡ **ትርጓሜ ወይም ገለጸ**

ሁለቱም መድኃኒቶች፣ ማለትም ያለ ክትትል ለ7-ቀን የሚሰጥ ከፍተኛ መጠን ያለው ፕሪማኩዊን እና አንድ ጊዜ ብቻ የሚወሰድ ታፌኖኩዊን በቂ የG6PD መጠን ባላቸው ታካሚዎች በጥሩ ሁኔታ ተስማሚ ሲሆን፣ መጠኑ እርግጠኛ ባይሆንም ለ14-ቀን ከሚሰጥ ዝቅተኛ መጠን ያለው ፕሪማኩዊን ጋር ሲነጻጸር የፕላዝሞዲየም ቫይቫክስ ወባ የማገርሸት አደጋም ዝቅተኛ ነበር፡፡

ግኝቶቻችን በተለያዩ የወባ በሽታ በሚከሰትባቸው አካባቢዎች የአጭር ጊዜ ሥር ነቀል የሕክምና ዘዴዎች ውጤታማነት እና ተግባራዊ ሊሆኑ መቻላቸውን ይደግፋሉ።

**የገንዘብ ድጋፍ ሰጪዎች፡** የአውስትራሊያ ብሔራዊ የጤና እና የህክምና ምርምር ምክር ቤት (NHMRC) እና የቢል እና ሜሊንዳ ጌትስ ፋውንዴሽን።

**ቁልፍ ቃላት፡** ፕላዝሞዲየም ቫይቫክስ፣ ቫይቫክስ ወባ፣ ሥር-ነቀል ፈውስ፣ ውጤታማነት፣ ፕሪማኩዊን፣ ታፌኖኩዊን።

## Abstract (id)

**Latar Belakang**

Primakuin durasi singkat dan tafenokuin dosis tunggal memiliki kemampuan untuk meningkatkan efektivitas dalam mencegah infeksi *Plasmodium vivax*, namun data perbandingan efektivitas kedua obat tersebut jika diberikan tanpa pengawasan masih terbatas. Adapun tujuan penelitian ini adalah untuk menilai efektivitas dan keamanan kedua pilihan terapi ini.

**Metode**

Kami melakukan studi uji superioritas multisenter, acak terbuka, terkontrol di Etiopia, Pakistan, Indonesia dan Kamboja. Pasien dewasa (berusia ≥ 18 tahun, atau ≥ 16 tahun di Indonesia) dengan infeksi *P. vivax* tanpa komplikasi dan aktivitas glucose-6-phosphate dehydrogenase (G6PD) lebih atau sama dengan 70% dimasukkan sebagai subjek penelitian. Pasien diterapi dengan obat skizontosidal darah (klorokuin di Etiopia dan Pakistan, dihydroartemisinin-piperakuin di Indonesia dan artesunate-pyronaridine di Kamboja) dan kemudian diacak (1:1:1) oleh seorang ahli statistik independen menggunakan randomisasi blok yang disusun secara acak dengan berbagai ukuran untuk mendapatkan 7 hari primakuin dosis tinggi (total dosis 7mg/kg), dosis tunggal tafenokuin (300mg) atau primakuin dosis rendah 14 hari (total dosis 3.5mg/kg). Randomisasi distratifikasi berdasarkan lokasi. Luaran primer, yang dinilai pada populasi *intention-to-treat* termodifikasi (yaitu pasien yang menerima terapi sesuai alokasi dan tidak hilang dari tindak lanjut sebelum hari ke-15) adalah insidens kumulatif terjadinya parasitemia *P. vivax* dalam waktu 6 bulan dibandingkan dengan kelompok primakuin. Sedangkan perbandingan insidens kumulatif antara kelompok tafenokuin dan kelompok primakuin dosis rendah selama 14 hari merupakan luaran sekunder. Luaran keamanan utama meliputi jumlah kejadian tidak diinginkan dan kejadian tidak diinginkan serius, termasuk jumlah gejala gastrointestinal serta risiko terjadinya anemia. Luaran keamanan dinilai pada semua pasien yang mendapatkan obat penelitian dosis berapapun. Penelitian ini telah diregistrasi pada ClinicalTrials.gov (NCT04411836) dan telah selesai.

Hasil

Antara 25 April 2021 hingga 13 September 2024, sebanyak 4850 pasien di skrining untuk kelayakan, diantaranya 960 pasien (672 [70,0%] laki-laki dan 288 [30,0%] perempuan) direkrut dan dilakukan randomisasi (320 pasien setiap lengan). Populasi *intention-to-treat* yang dimodifikasi terdiri dari 295 pasien yang dialokasikan ke kelompok primakuin dosis tinggi 7 hari, 305 pasien yang dialokasikan ke kelompok tafenokuin, dan 301 pasien yang dialokasikan ke kelompok primakuin dosis rendah selama 14 hari, yang dianggap berisiko mengalami kekambuhan *P. vivax* dari hari ke-15 dan seterusnya. Insiden kumulatif dari rekuren *P. vivax* pada 6 bulan adalah 13·0% (97.55% CI 9·0-18·5) pada kelompok primakuin dosis tinggi 7-hari dan 18·5% (97.55% CI 13·8-24·6) pada kelompok primakuin dosis rendah 14-hari (Hazard Ratio (HR) =0·66; 97.55% CI 0·40-1·09, p=0·063). Angka kejadian yang bersesuaian pada kelompok tafenokuin adalah 12·6% (97.55% CI 8·8-18·0); HR=0·64 (97.55% CI 0·39-1·05, p=0·041) dibandingkan pada kelompok dosis rendah 14-hari. Berdasarkan kejadian tidak diinginkan sebelum hari ke-42, 24 dari 56 (42.9%) dilaporkan berkaitan dengan obat pada kelompok primakuin dosis tinggi 7-hari, dibandingkan dengan 16 dari 72 (22.2%) pada kelompok tafenokuin dan 13 dari 38 (34.2%) pada kelompok primakuin dosis rendah 14-hari. Empat kejadian tidak diinginkan serius muncul, dua diantaranya (gejala gastrointestinal pada dua pasien dari kelompok primakuin dosis tinggi 7 hari) dianggap kemungkinan berkaitan dengan obat studi. Satu kematian yang tidak berikaitan dengan obat studi muncul pada kelompok primakuin dosis rendah 14 hari.

**Kesimpulan**

Baik primakuin dosis tinggi 7-hari tanpa pengawasan ataupun tafenokuin dosis tunggal dapat ditoleransi dengan baik pada pasien dengan G6PD normal dan memiliki risiko rekurensi lebih rendah untuk *P. vivax* dibandingkan primakuin dosis rendah 14-hari, walaupun seberapa besar efeknya belum bisa dipastikan. Temuan kami mendukung efektivitas dan kelayakan operasional dari rejimen pengobatan radikal yang lebih singkat di berbagai wilayah endemik malaria.

## Abstract (km)

**សចកផម**

វគពបល radical cure រយពលខដយបថ primaquine​ រយពល៧ថ នងថ tafenoquine មងតមយ​​ ដសមនសកនពលដមបងនបសទភពនករអនវតករពបល កងករករពរករលបឡងវញនករឆង ​​Plasmodium vivax បនទននយបបធបកមលកខណផលថដយគនករតតពនតនតមនកមត។ ករសកនមនគលបណងវយតមបសទភព នងសវតភពនវគពបលខទងន។

**វធសស**

ករសកលងវជសសចដនបកចហពហមជមណល តវបនអនវតនបទស អតព បគសន ឥណនស នងកមជ លអកជងពញវយមន P. vivax ​ គនចញសល នងសកមភព G6PD ≥ ៧០%ឬមនកមតសកមភព G6PD ចនជងនតវបនអនញតឱចលរមករសក ។ អកជងទងអសបនទទលថសមបបរ សត​គនចញកងឈមដចជថ chloroquine ន​​​​អតព នងបគសន​ ​ ថdihydroartemisinin-piperaquine នឥណនស នង ​ថ​artesunate​ pyronaridine នកមជ។ បនបមកបងចក ចដន (១:១:១) ទថ primaquine ៧ថកមតខស (៧ mg/kg), ​ថ tafenoquine ៣០០ mg មងតមយដស, ឬ ​ថ primaquine ១៤ថកមតទប (៣.៥ mg/kg)។ ចណចបញបចមងគអតលបឡងវញនបរសតមរគ P. vivax កងរយពល ៦ខ ដយបបធបចមងរវងថ primaquine ៧ថ នងថ primaquine ១៤ថ។ ករសកនបនចបញន ClinicalTrials.gov (NCT04411836)។

**លទផល**

ចនខមស ២០២១ ដលខកញ ២០២៤ មនអកចលរមសរប ៩៦០ នក (៣២០ នកកងមយកម)។ អតលបឡងវញកងរយពល ៦ខគ ១៣.០% នកម primaquine ៧ថ នង ១៨.៥% នកម primaquine ១៤ថ (HR = 0.66; 97.55% CI 0.40–1.09; p=0.063)។ កម tafenoquine មនអត ១២.៦% (HR = 0.64; 97.55% CI 0.39–1.05; p=0.041) បបបធបទនង primaquine ១៤ថ។ លទផលបងញននករកតបនយហនភយនករលបឡងវញជមយវគពបលខ បនចនជជករមបញលតម ១ ដលបងញពភពមនបកដនទហផលបពល។

ផលរខននឱសថពកពនថមនថទ ៤២ មនសមមតខសជងគនកម primaquine ៧ថ (៤៣%) បបបធបទនង tafenoquine (២២%) នង primaquine ១៤ថ (៣៤%)។ មនពតករណអវជមនផលរខននឱសថធនធរពកពនថចនន ២ កតឡងនកម primaquine ៧ថមនរកឃញសញសវតភពថឡយ។

**ករបកសយ**

វគពបលថ primaquine ៧ថ នងថ tafenoquine មងតមយដស តវបនទទលយកបនលនអកជងមនសកមភព G6PD ធមត នងបងញននករកតបនយហនភយនករលបឡងវញ បបបធបទនងថ primaquine ១៤ថ ទបជទហផលបពលនតមនបកដកដយ។ លទផលទងនគទសកនពលបតបតករនវគពបល radical cure រយពលខ កងបរបទលបបបតជងគនចញ។

## Abstract (ur)

**پس منظر**

پریماکوئن کے مختصر دورانیے کے علاج اور ٹیفینوکوئن کی واحد خوراک پلازموڈیم ویواکس انفیکشن کی دوبارہ واپسی کو روکنے میں مؤثریت کو بہتر بنانے کی صلاحیت رکھتے ہیں، تاہم بغیر نگرانی کے دیے جانے کی صورت میں ان کے تقابلی اثرات سے متعلق معلومات محدود ہیں۔ ہم نے ان نئے علاج کی مؤثریت اور حفاظت کا جائزہ لینے کا ارادہ کیا۔

طریقۂ کار

ہم نے ایتھوپیا، پاکستان، انڈونیشیا اور کمبوڈیا میں ایک کثیرالمراکز، اوپن لیبل، رینڈمائزڈ کنٹرولڈ سپیریئرٹی ٹرائل منعقد کیا۔ غیر پیچیدہ پلازموڈیم ویواکس انفیکشن کے حامل بالغ مریض (عمر ≥18 سال، یا انڈونیشیا میں عمر ≥16 سال)، جن میں گلوکوز-6-فاسفیٹ ڈی ہائیڈروجنیز کی سرگرمی 70 فیصد یا اس سے زیادہ تھی، مطالعے میں شمولیت کے اہل تھے-مریضوں کا علاج خون میں شیزونٹوسائیڈل ادویات کے ذریعے کیا گیا (ایتھوپیا اور پاکستان میں کلوروکوئن، انڈونیشیا میں ڈائی ہائیڈروآرٹیمیسینن-پائپیراکوئن، اور کمبوڈیا میں آرٹی سونیٹ-پائرو ناریڈین)، اور اس کے بعد ایک آزاد شماریات دان نے مختلف سائز کے بلاکس کو تصادفی طور پر ترتیب دے کر(1:1:1) تین گروپوں میں تقسیم کیا تاکہ وہ یا تو 7 دن تک بغیر نگرانی کے زیادہ خوراک والی پریماکوئن (کل خوراک 7 ملی گرام/کلوگرام)، یا ٹیفینوکوئن کی واحد خوراک (300 ملی گرام)، یا 14 دن تک کم خوراک والی پریماکوئن (کل خوراک 3.5 ملی گرام/کلوگرام) حاصل کریں۔ ہر مقام پر بےترتیبی تقسیم کی گئی ۔پرائمری اینڈ پوائنٹ کو modified intention-to-treat آبادی (یعنی وہ مریض جنہیں مقررہ علاج دیا گیا اور جو پندرہویں دن سے پہلے فالو اَپ سے محروم نہیں ہوئے) میں جانچا گیا۔ چھ ماہ کے اندر کسی بھی پلازموڈیم ویویکس پیراسائٹیمیا کے مجموعی وقوعے کا موازنہ پرائماکوئین کے گروپوں کے درمیان کیا گیا، جبکہ ٹیفینوکوئین گروپ اور چودہ دن کی کم خوراک پرائماکوئین گروپ کے درمیان مجموعی وقوعے کا موازنہ ثانوی اختتامی نکتہ تھا۔ حفاظتی نتائج میں مضر اور سنگین مضر واقعات کی تعداد شامل تھی، جن میں معدے سے متعلق علامات کی تعداد کے ساتھ ساتھ خون کی کمی (انیمیا) کے خطرے کا جائزہ بھی شامل تھا۔ حفاظتی نتائج کا تجزیہ ان تمام مریضوں میں کیا گیا جنہیں مطالعے کی دوا کی کوئی بھی خوراک دی گئی تھی۔اس تحقیق کو ClinicalTrials.gov (NCT04411836) میں رجسٹر کیا گیا تھا اور یہ مکمل ہو چکا ہے۔

اپریل25 2021 سے 13 ستمبر 2024 کے درمیان، 4850 مریضوں کی اہلیت کے لیے جانچ کی گئی، جن میں سے 960 مریض (672 [٪70] مرد اور 288 [٪30] خواتین) شامل کیے گئے اور تصادفی طور پر تقسیم کیے گئے۔ ہر گروپ میں 320 مریض تھے۔ ترمیم شدہ علاج کے ارادے کے مطابق آبادی میں شامل 295 مریض جو 7 دن کی زیادہ خوراک پرائماکوئین گروپ میں تقسیم کیے گئے، 305 مریض جو ٹیفینوکوئین گروپ میں تقسیم کیے گئے، اور 301 مریض جو 14 دن کی کم خوراک پرائماکوئین گروپ میں تقسیم کیے گئے، پندرہویں دن کے بعد پلازموڈیم ویویکس کے دوبارہ ہونے کے خطرے میں سمجھے گئے۔ چھ ماہ کے عرصے میں P. vivax کی دوبارہ بیماری کی مجموعی شرح سات دن کے زیادہ خوراک والے پرائماکوئن گروپ میں 13 فیصد تھی (اعتماد کی حد% 97.55: 9.0–18.5)، جبکہ چودہ دن کے کم خوراک والے پرائماکوئن گروپ میں یہ شرح 18.5 فیصد تھی (اعتماد کی حد% 97.55: 13.8–24.6)۔

ٹیفینوکوئن گروپ میں اسی مدت کے دوران دوبارہ بیماری کی شرح 12.6 فیصد تھی (اعتماد کی حد %97.55: 8.8–18.0)

(Hazard Ratio (HR)= 0·64 (97.55% CI 0·39–1·05، p=0·041)

جو چودہ دن کے کم خوراک والے پرائماکوئن گروپ کے مقابلے میں بہتر تھ

بیا لیس دن سے پہلے پیش آنے والے مضر واقعات میں سے، 7 دن کی زیادہ خوراک پرائماکوئین گروپ میں 56 میں سے 24 (٪42.9) کو دوا سے متعلق سمجھا گیا، جبکہ ٹیفینوکوئین گروپ میں 72 میں سے 16 (٪22.2) اور 14 دن کی کم خوراک پرائماکوئین گروپ میں 38 میں سے 13 (٪34.2) کو دوا سے متعلق سمجھا گیا۔ چار سنگین مضر واقعات پیش آئے، جن میں سے دو (7 دن کی زیادہ خوراک پرائماکوئین گروپ میں دو مریضوں میں معدے سے متعلق علامات) کو ممکنہ طور پر مطالعے کی دوا سے متعلق سمجھا گیا۔ 14 دن کی کم خوراک پرائماکوئین گروپ میں ایک موت پیش آئی جو مطالعے کی دوا سے غیر متعلق تھی۔

تشریح

**غیر نگرانی شدہ 7 دن کی زیادہ خوراک پرائماکوئین اور واحد خوراک ٹیفینوکوئین دونوں کو ان مریضوں میں اچھے طریقے سے برداشت کیا گیا جن کی گلوکوز-6-فاسفیٹ ڈی ہائیڈروجنیز سرگرمی معمول کے مطابق تھی، اور ان میں پلازموڈیم ویویکس کے دوبارہ ہونے کا خطرہ 14 دن کی کم خوراک پرائماکوئین گروپ کے مقابلے میں کم تھا، حالانکہ اس کی شدت غیر یقینی تھی۔ ہمارے نتائج مختلف ملیریا سے متاثرہ علاقوں میں مختصر دورانیے کے ریڈیکل کیور علاجی طریقوں کی مؤثریت اور عملی قابلِ عملیت کی حمایت کرتے ہیں-**

## Introduction

*Plasmodium vivax* malaria continues to pose a substantial public health challenge in the Asia–Pacific region, the Horn of Africa, and the Americas.^1^ *P vivax* forms dormant liver-stage parasites, called hypnozoites, that can reactivate weeks or months after an initial infection, leading to recurrent episodes of malaria. These relapses contribute to an increased risk of severe anaemia, morbidity, and mortality.^2^ To prevent relapse, radical curative treatment is required, which consists of treatment of both the acute blood stages (schizonticidal treatment) and the dormant liver stages (hypnozoitocidal treatment) of the *P vivax* parasite. Primaquine and tafenoquine are the only available drugs capable of eliminating *P vivax* hypnozoites, but both can induce severe haemolysis in patients with glucose-6-phosphate-dehydrogenase (G6PD) deficiency.^3^ In the absence of routine G6PD testing, many *P vivax*-endemic countries recommend a low-dose course of primaquine (3·5 mg/kg total dose) administered over 14 days to reduce the risk of haemolysis.^4^ However, in routine clinical practice, adherence to the 14-day regimen is poor, and, when treatment is unsupervised, the effectiveness of the prolonged regimen has been reported to be as low as 12%.^5^, ^6^ Moreover, a meta-analysis demonstrated that, even when drug administration was supervised, patients treated with low-dose primaquine had almost twice the risk of recurrent *P vivax* malaria compared with those treated with high total doses of primaquine (7 mg/kg).^7^

**Research in context**

**Evidence before this study**

The first-line treatment of *Plasmodium vivax* malaria in most endemic countries is a low-dose primaquine regimen (total dose of 3·5 mg/kg) administered over 14 days. A systematic review and individual patient data meta-analysis using MEDLINE, Embase, Web of Science, and the Cochrane Database of Systematic Reviews identified 23 antimalarial clinical trials, published between Jan 1, 2000, and June 8, 2023, that assessed anti-relapse efficacy of primaquine in patients with *P vivax* malaria. 6879 patients were included in the analysis. High total dose (7 mg/kg) regimens of primaquine reduced the cumulative incidence of recurrences by 55% compared with the low-dose (3·5 mg/kg) regimen, and this finding was apparent in all *P vivax* endemic areas. The efficacy of high-dose primaquine administered over 14 days was similar to the same total dose administered over 7 days. High-dose primaquine was safe in patients with normal (≥70%) glucose-6-phosphate dehydrogenase (G6PD) activity.

A systematic review on tafenoquine included data from three drug registration trials published between October, 2010, and May, 2017, that compared the efficacy of tafenoquine in combination with chloroquine against low-dose primaquine in 1073 patients; the cumulative incidence at 4 months after a single 300 mg dose of tafenoquine was 15%. In a separate trial, in which 50 patients were treated with tafenoquine in combination with dihydroartemisinin–piperaquine and relocated to a non-endemic area to prevent the risk of reinfection, the cumulative incidence of recurrent parasitaemia within 6 months was 79%.

We searched MEDLINE (PubMed), Embase, and CENTRAL, plus ClinicalTrials.gov/WHO ICTRP, from database inception to Jan 15, 2026, using the terms “*P vivax”*, “tafenoquine”, and “primaquine”, combined with a randomised controlled trial filter. We additionally ran a sensitivity search adding adherence terms to identify programmatic and unsupervised comparisons. To date, no randomised clinical trials have compared the effectiveness and safety of an unsupervised course of high-dose or low-dose primaquine with a single dose of tafenoquine.

**Added value of this study**

Our multicentre, open-label, randomised, controlled, superiority trial evaluated the effectiveness and safety of a 7-day course of high-dose primaquine, a 14-day course of low-dose primaquine, and single-dose tafenoquine to prevent recurrent parasitaemia in patients presenting with uncomplicated *P vivax* malaria. In patients with normal G6PD activity, the high-dose primaquine and tafenoquine regimens were well tolerated and both treatments led to a substantial reduction in recurrences compared with patients treated with low-dose primaquine. The overall risk of recurrence following 300 mg tafenoquine was low. The predefined subgroup analysis by country showed a much lower risk of recurrence in patients in Cambodia treated with tafenoquine plus artesunate–pyronaridine than expected.

**Implications of all the available evidence**

The evidence supports the 2024 revision of the WHO guidelines, recommending the use of a high total dose of primaquine and the safety of the 1 mg/kg per day primaquine regimen in patients with normal G6PD activity. The results also support the use of tafenoquine in combination with chloroquine, in endemic areas outside of South America. The low risk of recurrence in patients treated with tafenoquine plus artesunate–pyronaridine challenges current recommendations that tafenoquine should only be co-administered with chloroquine and highlights a need for further studies to quantify the risk of recurrence of tafenoquine with different artemisinin-based combination therapies.

The efficacy of primaquine is related to the total dose administered.^7^ Shorter 7-day regimens have shown similar efficacy to the same total dose administered over 14 days.^8^, ^9^ However, the higher daily dose, required for the short-course regimen (1 mg/kg per day), is associated with greater risk of gastrointestinal intolerance and, in patients with reduced G6PD activity, a greater risk of haemolysis.^10^ The 2024 WHO guidelines recommended a high total dose of primaquine administered over 14 days or 7 days, but restricted the use of the 7-day regimen to patients with normal (≥70%) G6PD activity.^11^

Tafenoquine is the first new anti-relapse treatment to have been licensed in the past 70 years and represents a major advance in the treatment of malaria. Because of its extended half-life (approximately 14 days), tafenoquine can be given as a single-dose treatment, thus overcoming the challenge of adherence with prolonged primaquine regimens (4–7 h half-life). The pivotal phase 3 clinical trials compared 300 mg of tafenoquine with low-dose primaquine (3·5 mg/kg); in a pooled analysis the efficacy of tafenoquine was similar but not non-inferior to low-dose primaquine.^12^ Tafenoquine is currently restricted to use with chloroquine, the blood schizonticidal treatment used in the licensing trials.^12^, ^13^ In a randomised trial of soldiers infected with *P vivax* in Papua, Indonesia, the risk of *P vivax* recurrence at 6 months was 79% when tafenoquine was combined with dihydroartemisinin–piperaquine, an artemisinin combination therapy (ACT).^14^ Although the low efficacy of this combination has been attributed to a drug–drug interaction,^15^ an alternative hypothesis is that tafenoquine was under-dosed, particularly in an area with high relapse frequency and high parasite burden.^16^, ^17^

There has been no direct randomised comparison of safety and efficacy or effectiveness of high-dose primaquine and tafenoquine. We conducted a multicentre, open-label, randomised controlled trial to assess the effectiveness and safety of unsupervised 7-day high-dose primaquine (7 mg/kg total dose) and single-dose tafenoquine (300 mg fixed dose) in patients with normal G6PD activity and *P vivax* malaria compared with low-dose primaquine (3·5 mg/kg total dose) given over 14 days.

## Methods

### Study design

The EFFORT trial is a multicentre, open-label, randomised, controlled, superiority trial in patients with *P vivax* malaria and normal G6PD (≥70%) activity. The study was conducted at sites in Cambodia (Kravanh, Siem Pang, and Chambak), Ethiopia (Arba Minch), Indonesia (Batubara, Sumatra), and Pakistan (Karachi and Thatta, Sindh; appendix 1 pp 3–5, 8; appendix 2). Ethical and drug regulatory approvals were obtained from the Human Research Ethics Committee of the Northern Territory Department of Health and Menzies School of Health Research and by the relevant national and institutional independent review boards and regulatory authorities at the participating study sites (appendix 1 pp 6–7). The study was overseen by an independent Data Safety Monitoring Board (appendix 1 pp 9–13). Protocol violations and deviations and details of patient or public involvement in the design, conduct, and reporting of the trial are reported in appendix 1 (pp 9–15). Our trial is reported in accordance with the CONSORT guidelines, including the CONSORT extension for better reporting of harms in randomised trials (appendix 1 pp 16–22). The study is registered at ClinicalTrials.gov, NCT04411836.

### Participants

Adult patients (aged ≥18 years, or ≥16 years in Indonesia) presenting with uncomplicated *P vivax* mono-infection (or mixed infection with *P vivax* and *Plasmodium falciparum* in Indonesia), as determined by microscopy, who had fever (axillary temperature ≥37·5°C) or a history of fever in the preceding 48 h, were eligible for enrolment. Eligible patients were also required to have G6PD activity of 70% or higher of the site-specific adjusted male median (AMM)^18^ based on readings from the STANDARD G6PD (SD Biosensor, Gyeonggi-do, South Korea; appendix 1 p 23), live in the study area, and be willing to be followed up for 6 months. Patients were excluded from enrolment if their haemoglobin measured by the STANDARD G6PD was less than 8 g/dL, they were pregnant or lactating, they had a known allergy or sensitivity to any of the study drugs, they had danger signs or symptoms of severe malaria, or they had a history of regular use of drugs with haemolytic potential. Written informed consent was obtained from all patients, and assent from patients younger than 18 years at the Indonesian site, before enrolment.

### Randomisation and masking

Patients were treated with a blood schizonticidal treatment according to national protocols and randomly assigned (1:1:1) to one of three treatment groups: high-dose primaquine (total dose 7 mg/kg) administered over 7 days (high-dose primaquine group), single-dose tafenoquine (300 mg; single-dose tafenoquine group), or low-dose primaquine (total dose 3·5 mg/kg) administered over 14 days (low-dose primaquine group or control group). Randomisation was stratified by study site, using randomly permuted blocks of varying sizes. The randomisation list was prepared by an independent statistician, and individual allocations were sealed in opaque envelopes and only opened after screening was completed by the qualified study team (appendix 1 p 24). All treatments were open label.

### Procedures

Data on sex were collected via self-report. Patients were treated as outpatients and received a 3-day schizonticidal drug regimen (chloroquine in Ethiopia and Pakistan, dihydroartemisinin–piperaquine in Indonesia, and artesunate–pyronaridine in Cambodia). Patients in the control group were treated with 14 days of oral primaquine (0·25 mg/kg per day; 3·5 mg/kg total dose). Patients in the intervention groups were treated with either 7 days of oral primaquine (1 mg/kg per day; 7 mg/kg total dose) or a single dose of oral tafenoquine (300 mg). Schizonticidal treatment and primaquine were provided according to bodyweight, whereas tafenoquine was provided as a fixed dose for all patients (appendix 1 pp 25–30). The first dose of schizonticidal drug and study drug were administered on the day of enrolment under supervision. Patients were instructed to take the remaining treatment at home (unsupervised treatment). Chloroquine, dihydroartemisinin–piperaquine, and primaquine were obtained from the respective national malaria control programmes, artesunate–pyronaridine was donated by Shin Poong Pharmaceutical (Seoul, South Korea) and tafenoquine (Kodatef, Batch number 84961) was purchased from Biocelect (Sydney, NSW, Australia).

To minimise interference with adherence, follow-up during treatment was limited to a single visit on day 3, conducted to ensure clinical recovery and detection of early signs of adverse events. After completion of treatment, participants were reviewed weekly from day 21 until day 42 and thereafter monthly until 6 months. At each visit, a medical history was taken, a symptom questionnaire was completed, and any adverse events or serious adverse events were recorded. A capillary or venous blood sample was collected at each visit for malaria microscopy and haemoglobin measurement by STANDARD G6PD. Patients in the single-dose tafenoquine group had two additional scheduled visits on day 7 and day 14 for safety monitoring, including symptom questionnaires, adverse event assessment, and measurement of haemoglobin.

Patients with recurrent *P vivax* mono-infection at or after day 28 were treated as per their allocation at enrolment and remained in follow-up. Patients presenting with recurrent *P vivax* malaria before day 28 were offered rescue treatment as per national guidelines. Patients presenting with *P falciparum* or a mixed infection at any time during the follow-up period were treated per the national guidelines with schizonticidal treatment but no hypnozoitocidal treatment.

Molecular analysis of the parasite was done on venous or capillary blood samples collected at enrolment and recurrence. DNA was extracted using commercial kits (Qiagen; Hilden, Germany) and *Plasmodium* species was determined using PCR.^19^ All *P vivax*-positive isolates were genotyped using a 93-microhaplotype marker panel on the Illumina sequencing platform (appendix 1 p 31).^20^

### Outcomes

The primary outcome was the cumulative incidence (time to first event) of any *P vivax* parasitaemia (symtomatic and asymtomatic) microscopically confirmed between day 15 and 6 months in the 7-day high-dose primaquine group and the 14-day low-dose primaquine group, with the comparison of the single-dose tafenoquine group with the 7-day high-dose primaquine group and the 14-day low-dose primaquine group serving as key secondary outcomes. Other secondary outcomes at 6 months were the cumulative incidence of symptomatic episodes, and incidence rate (events per person-time) of any *P vivax* parasitaemia and symptomatic *P vivax* parasitaemia.

The safety endpoints included the risk of severe (haemoglobin <5 g/dL) or moderate (≥5 g/dL and <7 g/dL) anaemia or need for blood transfusion, the risk of an acute decrease in haemoglobin of more than 5 g/dL from baseline, and the risk of an acute decrease in haemoglobin of more than 25% to less than 7 g/dL within 3 days of treatment initiation. Other safety endpoints included the number of adverse and serious adverse events, including gastrointestinal symptoms. Relatedness to study drug (certain, probably, possible, or unlikely) was assessed by the treating clinician and discussed with the study team. All prespecified outcomes are listed in appendix 2 (protocol).

### Statistical analysis

A sample size of 720 participants was calculated, assuming 95% power to detect a reduction in the risk of recurrent *P vivax* parasitaemia within 6 months from 36% in the control group^5^ to 20% in the 7-day high-dose primaquine group^8^ (expected hazard ratio [HR] 0·5), and 80% power to detect a reduction in the risk of recurrence of *P vivax* parasitaemia from 33% in the single-dose tafenoquine group^12^ to 20% in the 7-day high-dose primaquine group (expected HR 0·6), reflecting target effects. A two-sided α of 0·025 was chosen due to the three-arm trial design with a projected 10% loss to follow-up. To increase geographical representation, an additional 240 patients were included from Pakistan, increasing power to 98% for the primary effectiveness outcome, resulting in a total sample size of 960 patients (appendix 1 p 32).

The analyses for the effectiveness endpoints were conducted using a modified intention-to-treat approach, including all patients assigned to the treatment groups as randomised (1:1:1). The safety dataset included all randomly assigned patients who received any dose of study drug and were analysed according to treatment received. The cumulative incidence of *P vivax* parasitaemia between day 15 and 6 months was estimated by Kaplan–Meier analysis. Patients were censored for any non-vivax Plasmodium infections. Comparisons of relative hazards between the trial groups were done using a Cox proportional hazards model with stratification by study site. The incidence rates were calculated by dividing the number of *P vivax* episodes by the number of person-years of observation in the study population, with incidence rate ratios estimated using mixed-effects Poisson regression with random intercepts for study site. There was no penalisation for multiplicity for the secondary outcomes beyond presentation of 97·55% CIs. The formal definition of superiority, based on the sample size calculations performed, was p≤0·0245. The statistical analysis was conducted according to an a-priori statistical analysis plan (appendix 1 pp 34–35), including a subgroup analysis for the primary outcome stratified by country (appendix 1 p 46). An unplanned interim analysis was done at the availability of 6 months of follow-up data for 50% of the sample size and allowed stopping for superiority using a Haybittle–Peto boundary.^21^ As a result, the α for the main analysis of the three study groups was reduced to 0·0245 and 97·55% CIs are presented for the main analysis too. The results of the interim analysis were presented by an independent statistician in a closed session to the Data Safety Monitoring Board. No changes were made to the study as a result. As this was an unplanned interim analysis and the study team were not apprised of the results, no bias adjustment was done (appendix 1 pp 47–48).

Post-hoc sensitivity analysis for the primary outcome was done including only patients for whom *P vivax* parasitaemia at enrolment and recurrence events were confirmed by PCR. All analyses were done using Stata version 18.

### Role of the funding source

The funders of the study had no role in study design, data collection, data analysis, data interpretation, or writing of the report.

## Results

**Figure 1:**
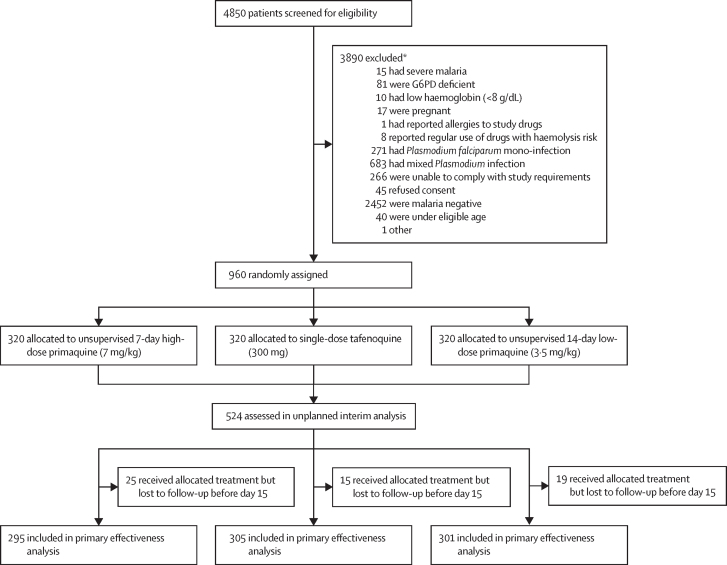
Trial profile G6PD=glucose-6-phosphate-dehydrogenase. *Not all screening tests were performed on every patient (eg, when one exclusion criterion was reached no further screening tests were conducted). The unplanned interim analysis was done approximately 2 years after the first patient was recruited.

Between April 25, 2021, and Sept 13, 2024, 4850 patients were screened for eligibility (appendix 1 p 49), of whom 960 (19·8%) were enrolled in the study and randomly assigned (320 in each group; figure 1). Baseline characteristics were similar between treatment groups (table 1). Overall, the median age was 26·0 years (IQR 20·0–36·5). Patients were predominantly male (672 [70·0%] patients), and this proportion was highest in Cambodia (198 [90·0%] patients; appendix 1 pp 50–51). At enrolment, median *P vivax* parasitaemia was 3360 parasites per μL (IQR 1312–8048) and the mean haemoglobin concentration was 13·9 g/dL (SD 2·2). Based on the site-specific AMM (appendix 1 p 23) there were 142 (14·8%) patients recruited with G6PD activities between 5 U/gHb and <6 U/gHb, below the manufacturer recommended cutoff, of whom 106 (74·7%) patients were male and 36 (25·3%) were female (table 1).

**Table 1:** Baseline characteristics of patients enrolled in the study (intention to treat)

			**7-day high-dose primaquine (n=320)**	**Single-dose tafenoquine (n=320)**	**14-day low-dose primaquine (n=320)**
Site					
	Cambodia		73 (22·8%)	73 (22·8%)	74 (23·1%)
		Kravanh	34 (10·6%)	32 (10·0%)	34 (10·6%)
		Siem Pang	39 (12·2%)	39 (12·2%)	39 (12·2%)
		Chambak	0	2 (0·6%)	1 (0·3%)
	Ethiopia (Arba Minch)		117 (36·6%)	117 (36·6%)	116 (36·2%)
	Indonesia (Batubara, Sumatra)		50 (15·6%)	50 (15·6%)	50 (15·6%)
	Pakistan		80 (25·0%)	80 (25·0%)	80 (25·0%)
		Thatta	72 (22·5%)	73 (22·8%)	73 (22·8%)
		Karachi	8 (2·5%)	7 (2·2%)	7 (2·2%)
Age, years			26·0 (20·0–36·0)	25·0 (20·0–36·5)	26·0 (20·0–37·0)
Sex					
	Male		228 (71·2%)	215 (67·2%)	229 (71·6%)
	Female		92 (28·7%)	105 (32·8%)	91 (28·4%)
Height, m			1·6 (1·6–1·7)	1·6 (1·6–1·7)	1·6 (1·6–1·7)
BMI, kg/m^2^			21·2 (19·1–23·9)	21·1 (19·2–23·9)	21·1 (18·8–23·5)
Bodyweight, kg			57·0 (51·0–65·0)	56·0 (51·0–64·0)	56·0 (51·0–63·4)
*Plasmodium vivax* parasites per μL			3360 (1312·0–7824·0)	3360 (1224·0–8392·0)	3344 (1432·0–8168·0)
Fever at presentation*			90 (28·1%)	110 (34·4%)	105 (32·8%)
Body temperature, °C			37·0 (36·6–37·6)	37·1 (36·7–37·8)	37·0 (36·7–37·7)
History of fever in previous 48 h			319 (99·7%)	320 (100·0%)	320 (100·0%)
Haemoglobin, g/dL			13·9 (2·2)	13·8 (2·3)	14·0 (2·1)
G6PD activity, U/g haemoglobin			7·2 (6·2–8·4)	7·2 (6·3–8·5)	7·3 (6·2–8·4)
G6PD activity <6 U/gH†			49 (15·3%)	45 (14·1%)	48 (15·0%)

Data are n (%), median (IQR), or mean (SD), unless otherwise specified. G6PD=glucose-6-phosphate dehydrogenase.

* Axillary temperature of ≥37·5°C.

† G6PD activity for enrolment was based on the adjusted male median activity per site (100% activity ranged between 6·45 U/gHb and 7·73 U/gHb; appendix 1 p 23), thus some patients with less than the 6 U/gHb cutoff usually applied for tafenoquine were included in the analysis.

All randomly assigned patients received the study drug. At the start of treatment, one (0·3%) patient treated in the 7-day high-dose primaquine group and one (0·3%) patient in the 14-day low-dose primaquine group vomited within 60 min, compared with none of the patients treated with tafenoquine. The study drug was readministered to both patients without further vomiting.

The mean total dose of primaquine provided was 7·6 mg/kg (range 5·1–11·1) in the 7-day high-dose primaquine group and 4·0 mg/kg (2·2–7·9) in the 14-day low-dose primaquine group. The mean dose of tafenoquine prescribed to patients in the tafenoquine group was 5·3 mg/kg (range 2·5–8·3; appendix 1 p 52). The total dose of study drugs provided did not differ across countries (appendix 1 p 53).

An unplanned interim analysis was conducted approximately 2 years after the first patient was recruited to enable analysis of data from three sites only, due to the protracted start of the study across all the sites.

The interim analysis used data from 524 patients, examining the primary and key secondary effectiveness outcomes. A Haybittle–Peto boundary was considered to allow early stopping, but this was not reached and hence recruitment continued until the planned sample size was reached.

59 (6·1%) of 960 patients were lost to follow-up between day 0 and day 15 and excluded from the primary outcome analysis: 25 (7·8%) in the 7-day high-dose primaquine group, 19 (5·9%) in the 14-day low-dose primaquine group, and 15 (4·7%) in the tafenoquine group (figure 1).

**Figure 2:**
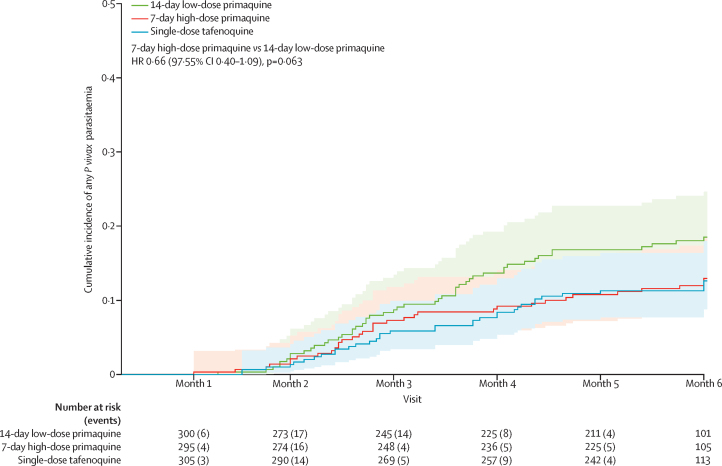
Cumulative incidence of the first recurrence of *Plasmodium vivax* parasitaemia Shading depicts 97·55% CIs. The HR for the primary comparison is shown. HR=hazard ratio.

The cumulative incidence of any *P vivax* recurrence at 6 months was 13·0% (97·55% CI 9·0–18·5) in the 7-day high-dose primaquine group and 18·5% (13·8–24·6) in the 14-day low-dose primaquine group (HR 0·66 [97·55% CI 0·40–1·09], p=0·063). The corresponding incidence in the tafenoquine group was 12·6% (97·55% CI 8·8–18·0), with an HR of 0·64 ([97·55% CI 0·39–1·05], p=0·041) for the comparison between the tafenoquine and 14-day low-dose primaquine groups and 0·96 ([0·56–1·66], p=0·875) when comparing the tafenoquine and 7-day high-dose primaquine groups (figure 2, table 2). The largest decrease in numbers at risk occurred between months 5 and 6 due to loss to follow-up.

**Table 2:** Cumulative incidence and incidence rate of any and symptomatic *Plasmodium vivax* parasitaemia by treatment group (modified intention-to-treat population)

		**7-day high-dose primaquine (n=295)**	**Single-dose tafenoquine (n=305)**	**14-day low-dose primaquine (n=301)**	**7-day high-dose primaquine *vs* 14-day low-dose primaquine**	**Tafenoquine *vs* 14-day low-dose primaquine**	Tafenoquine *vs* 7-day high-dose primaquine*
**Any *P vivax* parasitaemia**							
Cumulative incidence at 6 months (97·55% CI)		13·0% (9·0–18·5)	12·6% (8·8–18·0)	18·5% (13·8–24·6)	..	..	..
	HR (97·55% CI)	..	..	..	0·66 (0·40–1·09); p=0·063	0·64 (0·39–1·05); p=0·041	0·96 (0·56–1·66); p=0·875
Incidence rate per person-year (97·55% CI)		0·32 (0·23–0·46)	0·32 (0·23–0·45)	0·48 (0·36–0·63)	..	..	..
	IRR (97·55% CI)	..	..	..	0·67 (0·43–1·05); p=0·045	0·68 (0·44–1·06); p=0·050	1·01 (0·62–1·65); p=0·947
**Symptomatic *P vivax* parasitaemia**							
Cumulative incidence at 6 months (97·55% CI)		12·1% (8·3–17·5)	11·5% (7·9–16·6)	16·6% (12·1–22·6)	..	..	..
	HR (97·55% CI)	..	..	..	0·70 (0·42–1·19); p=0·129	0·66 (0·39–1·11); p=0·069	0·93 (0·53–1·64); p=0·78
Incidence rate per person-year (97·55% CI)		0·28 (0·19–0·40)	0·27 (0·19–0·38)	0·42 (0·32–0·57)	..	..	..
	IRR (97·55% CI)	..	..	..	0·65 (0·41–1·05); p=0·043	0·64 (0·40–1·02); p=0·032	0·97 (0·58–1·63); p=0·912

HR=hazard ratio. IRR=incidence rate ratio.

* This is an additional prespecified comparison outside of the multiplicity-adjusted α=0·0245 for the 7-day high-dose primaquine *vs* 14-day low-dose primaquine and tafenoquine *vs* 14-day low-dose primaquine comparisons.

The incidence rate for any *P vivax* parasitaemia was 0·32 (97·55% CI 0·23–0·46) recurrences per person-year in the 7-day high-dose primaquine group, 0·32 (0·23–0·45) in the tafenoquine group, and 0·48 (0·36–0·63) in the 14-day low-dose primaquine group (table 2).

**Figure 3:**
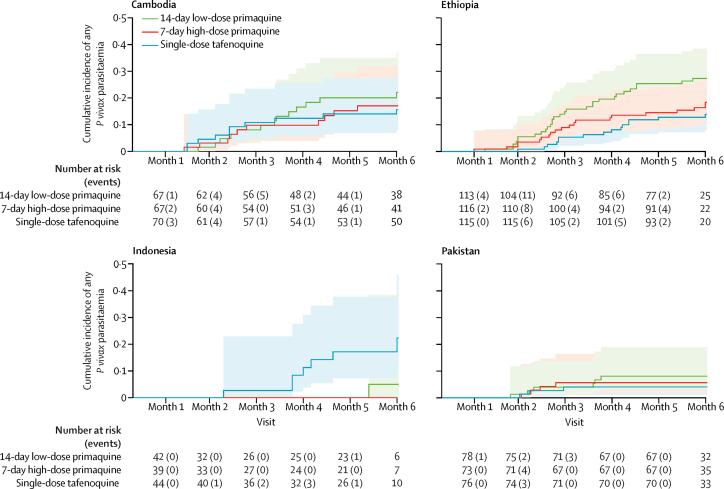
Cumulative incidence of the first recurrence of *Plasmodium vivax* parasitaemia, by country Shading depicts 97·55% CIs.

Subgroup analyses were prespecified for the primary and secondary outcomes (risk and rate). In a prespecified subgroup analysis, the effectiveness outcomes were assessed by country (figure 3, appendix 1 pp 54–56). Consistent with the main analysis, risk of *P vivax* recurrence at 6 months did not differ considerably between groups in this subgroup analysis, except in Indonesia where tafenoquine was combined with dihydroartemisinin–piperaquine. In Indonesia, the cumulative incidence of *P vivax* recurrence at 6 months was higher in the tafenoquine group (22·4% [97·55% CI 9·8–46·2]) compared with the 7-day high-dose primaquine group (0% [0·0–10·7]; HR not calculable) and the 14-day low-dose primaquine group (5·0% [0·5–38·5]; HR 5·47 [97·55% CI 0·49–60·53], p=0·112). However, in Cambodia, where tafenoquine was combined with artesunate–pyronaridine, the corresponding cumulative incidence in the tafenoquine group was 15·6% (97·55% CI 8·0–29·3), compared with 17·1% (8·7–31·8) in the 7-day high-dose primaquine group (HR 0·94 [97·55% CI 0·34–2·56], p=0·886) and 22·1% (97·55% CI 12·4–37·4) in the 14-day low-dose primaquine group (HR 0·72 [97·55% CI 0·28–1·85], p=0·432).

*P vivax* mono-infection was confirmed by PCR in 889 (92·6%) of 960 patients at enrolment (appendix 1 p 57), and in 24 (70·6%) of 34 patients with first recurrences in the 7-day high-dose primaquine group, 26 (74·3%) of 35 patients in the tafenoquine group, and 35 (71·4%) of 49 patients in the 14-day low-dose primaquine group (appendix 1 p 58). In a post-hoc sensitivity analysis excluding patients without confirmation of *P vivax* mono-infection at enrolment or at recurrence, the adjusted cumulative incidence of *P vivax* parasitaemia at 6 months was 10·5% (97·55% CI 6·8–16·0) in the 7-day high-dose primaquine group, 10·6% (7·0–16·0) in the tafenoquine group, and 14·1% (9·9–20·0) in the 14-day low-dose primaquine group (appendix 1 p 59).

Genotyping data on paired *P vivax* isolates at enrolment and recurrence were generated post hoc from 78 (90·6%) of 86 PCR-positive paired patient samples (appendix 3). Homologous recurrences were detected in nine (37·5%) of 24 recurrences in the 7-day high-dose primaquine group, 11 (47·8%) of 23 in the tafenoquine group, and 12 (38·7%) of 31 in the 14-day low-dose primaquine group (appendix 1 pp 60–61).

The median dose of tafenoquine was 5·4 mg/kg (IQR 4·7–6·0) in patients who did not have recurrent *P vivax*, 5·3 mg/kg (4·4–5·6) in patients who had a homologous recurrence, and 4·9 mg/kg (4·6–5·5) in patients who had a heterologous recurrence (appendix 1 pp 61–64).

Overall, 771 (80·3%) of 960 patients had haemoglobin concentrations recorded on both baseline and day 3, and 779 (81·1%) on baseline and day 28. On day 3, the mean decrease in haemoglobin from baseline was 0·2 g/dL (97·55% CI 0·0–0·5) in the 7-day high-dose primaquine group, 0·4 g/dL (0·2–0·7) in the tafenoquine group, and 0·5 g/dL in the 14-day low-dose primaquine group (0·2–0·7). Three patients, two in the tafenoquine group and one in the 14-day low-dose primaquine group, had a decrease in haemoglobin greater than 5 g/dL. All three patients had a high haemoglobin measurement (>15 g/dL) at enrolment (appendix 1 p 65). By day 28, haemoglobin exceeded the baseline value in 453 (58·2%) of 779 patients. No patient had a more than 25% decrease in haemoglobin to less than 7 g/dL, and no patient developed moderate or severe anaemia or required blood transfusion.

Among 818 patients with G6PD activity of 6 U/gHb or higher at baseline (screening), haemoglobin measurements on day 3 were available for 609 (74·4%) patients. The mean decrease in haemoglobin between baseline and day 3 was 0·1 g/dL (97·55% CI –0·2 to 0·4) in the 7-day high-dose primaquine group, 0·4 g/dL (0·1 to 0·6) in the tafenoquine group, and 0·4 g/dL (0·1 to 0·7) in the 14-day low-dose primaquine group. The corresponding decreases in the patients with G6PD less than 6 U/gHb who had a day 3 haemoglobin measurement (121 of 142 patients) were 0·7 g/dL (97·55% CI 0·3 to 1·2), 0·9 g/dL (0·2 to 1·5), and 0·7 g/dL (0·0 to 1·5), respectively (appendix 1 p 66).

On day 3, 729 (99·5%) of 733 patients were afebrile. Based on the checklist, symptoms reported on day 3 that had occurred since enrolment included gastrointestinal symptoms (at least one of vomiting, diarrhoea, loss of appetite, nausea, and abdominal pain) in 183 (75·6%) of 242 patients available in the 7-day high-dose primaquine group, 194 (81·2%) of 239 patients in the tafenoquine group, and 187 (75·1%) of 249 patients in the 14-day low-dose primaquine group (table 3).

**Table 3:** Safety and tolerability outcomes (safety population)

			**7-day high-dose primaquine (n=320)**	**Single-dose tafenoquine (n=320)**	**14-day low-dose primaquine (n=320)**
All serious adverse events			3	0	1
	Study drug related*		2	0	0
	Study drug unrelated		1	0	0
	Study drug unrelated resulting in death		0	0	1
All adverse events until day 42			56	72	38
	Study drug related*		24 (42·9%)	16 (22·2%)	13 (34·2%)
	Study drug unrelated		32 (57·1%)	56 (77·8%)	25 (65·8%)
	Grade 1†		49 (87·5%)	64 (88·9%)	31 (81·6%)
	Grade 2‡		6 (10·7%)	7 (9·7%)	7 (18·4%)
	Grade 3§		1 (1·8%)	1 (1·4%)	0
	Adverse events occurring from day 0 to 3		26 (46·4%)	20 (27·8%)	12 (31·6%)
	Adverse events occurring from day 4 to 42		30 (53·6%)	52 (72·2%)	26 (68·4%)
	Adverse events (expected) until day 42 grouped by body system		..	..	..
	Haemoglobin decrease (anaemia)		3 (5·4%)	3 (4·2%)	5 (13·2%)
	Gastrointestinal		22 (39·3%)	22 (30·6%)	11 (28·9%)
	Neuro-psychiatric		0	0	0
	Other adverse events until day 42 grouped by body system		..	..	..
	Respiratory		6 (10·7%)	22 (30·6%)	9 (23·7%)
	Musculoskeletal		4 (7·1%)	4 (5·6%)	1 (2·6%)
	Febrile illness		5 (8·9%)	10 (13·9%)	3 (7·9%)
	Generally unwell		11 (19·6%)	9 (12·5%)	4 (10·5%)
	Others		5 (8·9%)	2 (2·8%)	5 (13·2%)
Symptoms occurring since enrolment and reported on day 3					
	Any gastrointestinal symptoms¶		183/242 (75·6%)	194/239 (81·2%)	187/249 (75·1%)
		Vomiting	60/242 (24·8%)	65/239 (27·2%)	63/249 (25·3%)
		Nausea	98/242 (40·5%)	99/239 (41·4%)	94/249 (37·8%)
		Diarrhoea	9/242 (3·7%)	20/239 (8·4%)	9/249 (3·6%)
		Loss of appetite	147/242 (60·7%)	152/239 (63·6%)	145/249 (58·2%)
		Abdominal pain	46/242 (19·0%)	38/239 (15·9%)	45/249 (18·1%)
	Headache		221/242 (91·3%)	212/239 (88·7%)	225/249 (90·4%)
	Muscle pain		194/242 (80·2%)	170/239 (71·1%)	179/249 (71·9%)
	Joint pain		181/242 (74·8%)	176/239 (73·6%)	178/249 (71·5%)
	Fever		238/242 (98·3%)	236/239 (98·7%)	247/249 (99·2%)
	Dark urine		2/242 (0·8%)	4/239 (1·7%)	0/249
	Dizziness		131/242 (54·1%)	133/239 (55·6%)	132/249 (53·0%)
	Shortness of breath		11/242 (4·5%)	9/239 (3·8%)	6/249 (2·4%)
	Irritability		1/242 (0·4%)	3/239 (1·3%)	3/249 (1·2%)
	Jaundice		6/242 (2·5%)	1/239 (0·4%)	1/249 (0·4%)
	Fatigue		198/242 (81·8%)	200/239 (83·7%)	203/249 (81·5%)
	Malaise		145/242 (59·9%)	138/239 (57·7%)	142/249 (57·0%)
	Chills		222/242 (91·7%)	228/239 (95·4%)	237/249 (95·2%)

* Study drug-related events are possibly, probably, or definitely related to tafenoquine or primaquine.

† Mild symptoms causing no or minimal interference with usual activities.

‡ Moderate symptoms causing greater than minimal interference with usual activities.

§ Severe symptoms causing inability to perform usual activities.

¶ Includes at least any of the following: vomiting, diarrhoea, loss of appetite, nausea, and abdominal pain.

166 adverse events were recorded in 109 patients before day 42, of which 58 (34·9%) occurred within the first 3 days (table 3). Up to day 42, 56 (33·7%) events occurred in patients in the 7-day high-dose primaquine group, 72 (43·4%) in the tafenoquine group, and 38 (22·9%) in the 14-day low-dose primaquine group. Reporting of adverse events varied by study site, with 75 (45·2%) of 166 events occurring in Indonesia (appendix 1 pp 67–70). In the 7-day high-dose primaquine group, 43% (97·55% CI 29–58) of events were categorised as drug related compared with 34% (20–52) in the 14-day low-dose primaquine group and 22% (13–15) in the tafenoquine group (table 3). 55 (33·1%) of 166 adverse events were related to gastrointestinal symptoms, and 144 (86·7%) of 166 were categorised as mild and self-limiting (grade 1). Two patients reported grade 3 events, one of which, in the 7-day-high-dose primaquine group, was categorised as being related to the study drug (appendix 1 p 71).

Four serious adverse events were reported, including three in the 7-day high-dose primaquine group and one in the 14-day low-dose primaquine group (table 3, appendix 1 p 72). Two serious adverse events occurring in the 7-day high-dose primaquine group were associated with gastrointestinal symptoms and classified as probably drug related. A 27-year-old Cambodian male patient who had worsening nausea and vomiting after presenting with vomiting at enrolment and following initial study drug intake required hospital admission. The patient's symptoms resolved within 24 h after treatment with anti-emetics. The second patient was a 46-year-old male patient with known Gilbert syndrome enrolled in Pakistan, who presented with persistent nausea and vomiting on day 6, requiring hospital admission. This patient was discharged after 48 h but re-admitted on day 13 with jaundice, nausea, and vomiting. Symptoms resolved by day 21 and he made a full recovery. There were two serious adverse events classified as unrelated to study drug: a 45-year-old female patient in Pakistan with a history of hypertension who had a fatal myocardial infarction at month 4 and a 57-year-old Indonesian female patient who presented with appendicitis on day 35 requiring appendicectomy.

## Discussion

In this multicentre, open-label, randomised, controlled, superiority trial, the cumulative incidence of *P vivax* recurrence within 6 months was around 30% lower in patients treated with tafenoquine or 7-day high-dose primaquine than in patients treated with 14-day low-dose primaquine, even though absolute risks were lower than expected. The magnitudes of the HRs were as expected, although the p values were higher than the pre-planned α threshold for defining superiority of 0·0245. Both high-dose primaquine and tafenoquine were well tolerated and safe in patients with normal G6PD activity.

The cumulative incidence of *P vivax* recurrence at 6 months was 13% when high-dose primaquine was given unsupervised over 7 days. By contrast, efficacy estimates from supervised treatment trials indicate a cumulative incidence of only 8% with high-dose primaquine, independent of treatment duration,^8^ suggesting that adherence to short-course treatment in our study was suboptimal.^6^ Qualitative data suggest that pill burden poses a notable barrier to adherence,^22^ and this might have been particularly important in Ethiopia where 7·5 mg tablets of primaquine were used, necessitating up to 12 tablets per day for patients with a bodyweight of more than 70 kg.

Our study highlights the benefits of a single-dose regimen that avoids the issues of poor adherence inherent with prolonged primaquine regimens. Patients treated with tafenoquine had fewer recurrences than those treated with low-dose primaquine and comparable outcomes with those treated with the 7-day high-dose regimen. The cumulative incidence of *P vivax* recurrence after tafenoquine observed in our trial was 13%, which was better than expected based on data generated from tafenoquine registration trials, which ranged between 28% and 38%.^12^, ^13^ This variation likely reflects regional differences in the risk of relapse, host immunity, and the administered mg/kg dose of tafenoquine.^16^

Tafenoquine is recommended as a fixed 300 mg dose for all patients aged 16 years and older and weighing more than 35 kg, resulting in a large variation in the total mg/kg dose administered. A pooled meta-analysis of patients treated with tafenoquine estimated that increasing the dose of tafenoquine from the current target of 5·0 mg/kg (equivalent to the fixed dose of 300 mg) to 7·5 mg/kg would reduce the risk of recurrence by 90%.^16^ Trials are now underway to explore whether higher doses and weight-based dosing in adults could improve tafenoquine efficacy even further (NCT16148792; NCT04704999; NCT05788094).

The analysis of clinical trials of patients with *P vivax* malaria is complicated by the challenge of distinguishing whether recurrent infections are due to reinfection, recrudescence, or relapse. In this study, we applied a novel microhaplotype assay to detect highly related (defined here as homologous) recurrence pairs that likely represent relapses or recrudescence rather than reinfections.^20^ A total of 59% (46/78) of recurrences with paired genotyping were found to be heterologous and thus potential reinfections; hence, our estimates of effectiveness are likely to be conservative. The proportion of heterologous recurrences was higher in Ethiopia compared to Cambodia, potentially reflecting a greater contribution of reinfections to recurrences in this higher endemic setting.

The only previous study to assess the efficacy of tafenoquine co-administered with an ACT was conducted in Papua, Indonesia, where patients were removed from the endemic setting to eliminate the risk of new infections; the reported risk of recurrence at 6 months in patients treated with tafenoquine plus dihydroartemisinin–piperaquine was very high (79%).^14^ These findings raised concerns about a potential drug–drug interaction between tafenoquine and ACTs, prompting a revision of the drug label. As a result, tafenoquine has been restricted to patients treated with chloroquine, effectively precluding its use in 14 of the 41 *P vivax*-endemic countries that have adopted ACTs for all species of malaria.^23^ At the study sites in Cambodia, tafenoquine was administered with artesunate–pyronaridine and the cumulative incidence of *P vivax* at 6 months was 15·6%, similar to that following artesunate–pyronaridine plus 7-day high-dose primaquine. Our study did not include patients without an anti-relapse regimen to determine the background risk of recurrence. However, a separate trial in Cambodia reported that 81% of patients treated without a radical cure regimen had at least one recurrence by 3 months,^24^ suggesting that tafenoquine might retain good efficacy when administered with artesunate–pyronaridine. The data from Indonesia, where patients were treated with tafenoquine plus dihydroartemisinin–piperaquine, are more difficult to interpret since the risk of recurrence was higher in the tafenoquine group than in the primaquine groups. Without a control group, it is not possible to derive anti-relapse efficacy beyond the observed risk of recurrence. The divergent risk estimates together with the molecular data from paired samples,^25^ as well as timing of recurrences after tafenoquine with different ACTs, suggest that potential drug–drug interactions might not contribute equally to all antimalarial combinations and raise uncertainty that such interactions alone can account for the poor efficacy of tafenoquine reported in the INSPECTOR study.^14^, ^16^ Our results highlight the importance of further prospective clinical trials of tafenoquine with different ACTs.

The greatest concern for the introduction of high-dose primaquine and tafenoquine is severe haemolysis in individuals with reduced G6PD activity.^26^ Consistent with the tafenoquine label, we only enrolled patients with normal G6PD activity according to the G6PD STANDARD. The manufacturer defines G6PD normal activity as more than 6 U/gHb. In this study, a site-specific AMM was used instead, with 70% or higher activity of the AMM considered normal G6PD.^18^ Nearly 15% of recruited patients had a G6PD activity between 5 U/gHb and 6 U/gHb, including 11 female patients treated with 7-day high-dose primaquine and 14 female patients treated with tafenoquine, none of whom had haemolysis. Further studies are warranted to explore the haemolytic safety of new radical cure regimens in patients with activities less than the G6PD STANDARD threshold of 6 U/gHb.

Most of the reported adverse events were classified as mild and self-limiting and did not require medical intervention. The overall reporting of adverse events was higher in the tafenoquine group than in the primaquine groups, but this finding is likely a function of the additional clinical reviews on day 7 and 14 in the tafenoquine group. Almost a third of events were due to gastrointestinal complaints, as has been reported previously with high daily doses of primaquine exceeding 0·5 mg/kg per day.^10^ Two of the four serious adverse events included patients with gastrointestinal symptoms. Gastrointestinal tolerability can be improved by taking primaquine with food.^27^ In our study, the study team advised patients to take their tablets with food, although in routine practice this might be difficult.

An important aspect of our intervention was a routine clinical review on day 3, a timepoint identified to be best suited to identify patients at risk of severe haemolysis.^28^ Although such a review visit was achievable within the trial setting, integration into routine care is likely challenging. A clinical review can serve to improve the early diagnosis of impending haemolysis and can also be used as an opportunity to encourage patients to complete a full course of treatment. However, strategies to implement follow-up visits as part of routine *P vivax* case management are highly dependent on country context and will require collaboration across health system levels.^29^

Our study has several limitations. First, the lack of a comparator group including patients not treated with either tafenoquine or primaquine restricts the analysis to comparisons between treatment groups and prevents assessment of treatment efficacy compared with the background incidence of recurrence. During study planning, inclusion of a placebo or untreated group was considered but not adopted, as radical cure was already routine clinical practice at the study sites and withholding it was judged inappropriate in that context.^30^ Second, the study was powered for the overall analysis rather than for country-specific analyses or analysis by schizonticidal drug, resulting in wide confidence intervals for the latter, particularly in Indonesia where there was a substantial loss to follow-up. Our estimates of the cumulative incidence in the treatment groups for different countries need to be substantiated with appropriately powered prospective trials. Third, patients presenting during follow-up with mixed infections were treated with schizonticidal treatment but not with primaquine or tafenoquine. This might have affected our estimates of incidence rates, but not of the cumulative incidence of first *P vivax* recurrence, which was the primary endpoint. This limitation is particularly relevant for the Ethiopian site, where a large proportion of mixed species infections were reported. Fourth, late relapses occurring after 6 months will not have been captured, thus potentially overestimating treatment effectiveness, particularly in Pakistan where late latency relapses can occur.^31^ Fifth, the additional visit days in the tafenoquine group have likely biased the adverse event reporting, underestimating primaquine-related events. Sixth, although we aimed to conduct the study with minimal interaction during the treatment duration to reflect real-world conditions, the structured enrolment and consent process inherent to clinical trials potentially encouraged higher adherence compared with routine care settings, leading to an overestimate of treatment effectiveness in the primaquine groups. Seventh, the use of sealed envelopes for randomisation could potentially introduce a risk of allocation bias, although safeguards such as sequential numbering, opaque envelopes, and close monitoring were implemented to minimise this risk. Last, the exclusion of children limits the generalisability of the findings, particularly in high-burden settings where *P vivax* frequently affects paediatric populations.

In conclusion, unsupervised 7-day high-dose primaquine (7 mg/kg total dose) and tafenoquine (single 300 mg dose) were well tolerated in patients with normal G6PD activity and resulted in improved effectiveness compared to 14-day low-dose primaquine, which is currently recommended in most *P vivax*-endemic countries. Tafenoquine as a single-dose treatment has considerable operational advantages over 7-day high-dose primaquine. Our data support the geographical extension of the current tafenoquine recommendation in combination with chloroquine to high-burden areas such as Ethiopia and Pakistan. In contrast to previous findings, our trial showed good efficacy of tafenoquine with artesunate–pyronaridine and highlights the need for further trials to explore the use of tafenoquine in combination with different ACTs.

### Contributors

### Data sharing

The database is closed and the data are extracted and stored on WorldWide Antimalarial Resistance Network (WWARN.org) servers. De-identified individual participant data are available following application to the WWARN Data Access Committee. The *P vivax* genetic data generated in the trial will be deposited in the European Nucleotide Archive for open access upon publication.

## Declaration of interests

RNP is the pro-bono chair of the Data Safety Monitoring Board for a GSK-led trial titled: A randomized, open-label, multi-center, interventional phase 3 study of the efficacy and safety of tafenoquine compared to primaquine (both co-administered with chloroquine) for the radical cure (relapse prevention) of *Plasmodium vivax* (*P vivax*) malaria in Indian participants (paediatric and adult population). All other authors declare no competing interests.
