# Supplementary material for: Effectiveness and safety of 7-day high-dose primaquine and single-dose tafenoquine versus 14-day low-dose primaquine in patients with Plasmodium vivax malaria (EFFORT): a multicentre, open-label, randomised, controlled, superiority trial
Source: Lancet Infect Dis. 2026 Jun;26(6):614–26. doi: 10.1016/S1473-3099(25)00729-7 (PMC13215974; doi:10.1016/S1473-3099(25)00729-7)
Supplement: Supplementary appendix 1 [file mmc1.pdf]

THE LANCET
Infectious Diseases

Supplementary appendix 1

This appendix formed part of the original submission and has been peer reviewed. We post it as supplied by the authors.

Supplement to: Degaga TS, Pasaribu AP, Tripura R, et al. Effectiveness and safety of 7-day high-dose primaquine and single-dose tafenoquine versus 14-day low-dose primaquine in patients with *Plasmodium vivax* malaria (EFFORT): a multicentre, open-label, randomised, controlled, superiority trial. *Lancet Infect Dis* 2026; published online Feb 11. https://doi.org/10.1016/S1473-3099(25)00729-7.

Table of Contents

Table S1: Details of study sites 3

Table S2: List of Ethics Review Boards and Regulatory Authorities 6

Text S1: Database and Data Handling Procedures 8

Text S2: DSMB charter 9

Table S3: Protocol deviations and violations 14

Text S3: Patient or public involvement 15

Table S4: CONSORT 2025 checklist item description 16

Table S5: CONSORT Harms 2022 integrated into CONSORT 2010 items checklist 19

Table S6: CONSORT extension for abstracts 22

Table S7: Site-specific adjusted male median of G6PD activity 23

Text S4: Methodological details on randomisation 24

Table S8a: Dosing chart for patients in the 14-day-low-dose primaquine arm 25

Table S8b: Dosing chart for patients in the 7-day-high-dose primaquine arm 26

Table S8c: Dosing chart for patients in the Tafenoquine arm 27

Table S8d: Dosing chart for chloroquine for patients recruited in Ethiopia and Pakistan 28

Table S8e: Dosing chart for DHA-Piperaquine for patients recruited in Indonesia 29

Table S8f: Dosing chart for artesunate-pyronaridine for patients recruited in Cambodia 30

Text 5: Parasite genotyping and recurrence classification 31

Table S9: DELTA2 recommended reporting items for the sample size calculation of a randomised controlled trial with a superiority question 32

Text S6: Statistical Analysis Plan 34

Text S7: Details on handing of missing data 45

Text S8: Details of sub-group analysis 46

Text S9: Details on interim analysis 47

Figure S1: Interim analysis results 48

Table S10: Study sites and dates of enrolment 49

Table S11: Baseline characteristics by study country 50

Figure S2: Distribution of total primaquine doses administered in 7-day-high-dose and 14-day-low-dose primaquine arms, and total tafenoquine doses administered in the tafenoquine arm 52

Table S12: Total primaquine doses administered in the 7-day-high-dose and 14-day-low-dose primaquine arms, and total tafenoquine dose administered in the tafenoquine arm by country 53

Table S13: Cumulative incidence of any *P. vivax* recurrence at 6 months by country 54

Table S14: Total number of recurrences by study site 55

Table S15: Number of patients with multiple recurrences 56

Table S16: Summary of PCR confirmation of microscopy results of samples collected at enrolment....... 57
Table S17: Summary of PCR confirmation of microscopy results of samples collected at first *P. vivax* recurrence...... 58
Table S18: Sensitivity analysis excluding patients where *P. vivax* parasitaemia could not be confirmed by PCR (overall and by country) 59
Figure S3: Identity-by-descent distribution amongst baseline infections 60
Table S19: Homologous recurrences overall and per country 61
Figure S4: Tafenoquine dose (mg/kg) in patients with and without recurrences in Tafenoquine arm........ 62
Figure S5: Tafenoquine dose (mg/kg) in patients with homologous and heterologous recurrences and no recurrences in Tafenoquine arm 63
Figure S6: Tafenoquine dose (mg/kg) of patients with and without recurrences in each country in Tafenoquine arm 64
Table S20: Details of the three patients with an acute drop of haemoglobin (Hb) of >5g/dL by day 3...... 65
Figure S7: Change in haemoglobin between Day 0 and Day 3 in patients with G6PD <6 U/gHb in the 7-day-high-dose primaquine arm, tafenoquine arm and 14-day-low-dose primaquine arm 66
Table S21: Safety and tolerability outcomes, Cambodia 67
Table S22: Safety and tolerability outcomes, Ethiopia 68
Table S23: Safety and tolerability outcomes, Indonesia 69
Table S24: Safety and tolerability outcomes, Pakistan 70
Table S25: Details of the two patients with grade 3 (severe) adverse events 71
Table S26: Details of the four patients with serious adverse events (SAE) 72

Table S1: Details of study sites

	Cambodia	Ethiopia	Indonesia	Pakistan
**Study center(s)**	Kravanh District Hospital<br>Siem Pang Health Center<br>Chambak Health Center	Arba Minch General Hospital	Batubara Health Center, Sumatra	Khidmat-e-Alam Medical Center, Karachi<br>Thatta Civil Hospital
**Global Positioning System (GPS) coordinates of study center(s)**	12.1780/ 103.8216<br>14.1125/ 106.3905<br>11.2374/ 104.8098	6.025204 / 37.556696	3.1741/ 99.5006	24.8607/ 67.0011<br>24.7475/ 67.9106
**Altitude above sea level**	1,813m<br>70m<br>35m	1285m	29m	10m<br>13m
**Treatment policy (including G6PD testing)**	***P. falciparum***: AS-MQ<br>***P. vivax:*** AS-MQ + PQ for patients with normal G6PD activity 0.25-0.5 mg/kg over 14 days, and for G6PD deficient & females with intermediate G6PD activity, 0.75 mg/kg once a week for 8 weeks<br>**G6PD testing** is required as per guidelines (last edition 2022)	***P. falciparum***: AL+PQ (single dose 0.25 mg/kg)<br>***P. vivax:*** CQ+PQ (0.25 mg/kg for 14 days)<br>**G6PD testing** not required as per guidelines (last edition 2018)	***P. falciparum***: DHAP+PQ (single dose 0.25 mg/kg)<br>***P. vivax:*** DHAP+PQ (0.25 mg/kg for 14 days), 0.5mg/kg over 14 days (for relapse)<br>**G6PD testing** not explicitly mentioned in guidelines (last edition 2020) but required for relapsing cases with higher dose treatment. Testing however not implemented.	***P. falciparum***: AL+PQ (single dose 0.25 mg/kg)<br>***P. vivax:*** CQ+PQ (0.25 mg/kg for 14 days)<br>**G6PD testing** not required but can be done if available
**G6PD variant**	Viangchan variant[1]	Unknown prevalence,<br>likely A- and Mediterranean	8%[2],<br>Vanua lava, Chatham, Viangchan[3]	2-9%, Most commonly Mediterranean variant[4]
**Approximate catchment population of study center**	50,000<br>24,000<br>12,834	164,529	30,000	2,971,626<br>979,817
**Seasonality of malaria**	Year-round with a peak between May and October	Year-round with a peak in between December and February	Year-round with a peak between September and February	Year-round with a peak between April and October

**Ratio between vivax and falciparum malaria cases**	Unknown	30:70	15:85	80:20
**Relapse periodicity**	Unknown	3-5 months[5]	3 months	In temperate regions and parts of the sub-tropics *P. vivax* infections are characterized either by a long incubation or a long-latency period between illness and relapse - in both cases approximating 8-10 months[6]
**Vectors**	*A. dirus, A. maculatus and A. minimus*[7]	*A. arabiensis*	*A. sundaicus, A. subpictus*, *A. barbitrosis*[8]	*A. culicifacies*, *A. stephensi* [9]
**Annual Parasite Index (API)**	Unknown	Unknown	1.16[10]	11.03[11]

AS-MQ=artesunate-mefloquine, PQ=primaquine, AL=artemether-lumefantrine, DHAP= dihydroartemisinin-piperaquine, CQ=chloroquine, G6PD=Glucose-6-phosphate-dehydrogenase

[1]Matsuoka H, Nguon C, Kanbe T, Jalloh A, Sato H, Yoshida S, et al. Glucose-6-phosphate dehydrogenase (G6PD) mutations in Cambodia: G6PD Viangchan (871G>A) is the most common variant in the Cambodian population. Journal of Human Genetics 2005 50:9 [Internet]. 2005 Sep 1 [cited 2025 Apr 9];50(9):468–72. Available from: https://www.nature.com/articles/jhg200570

[2]Shimizu H, Tamam M, Soemantri A, Ishida T. Glucose-6-phosphate dehydrogenase deficiency and Southeast Asian ovalocytosis in asymptomatic Plasmodium carriers in Sumba island, Indonesia. J Hum Genet [Internet]. 2005 Sep [cited 2025 Apr 9];50(8):420–4. Available from: https://pubmed.ncbi.nlm.nih.gov/16059744/

[3]Satyagraha AW, Sadhewa A, Baramuli V, Elvira R, Ridenour C, Elyazar I, et al. G6PD deficiency at Sumba in Eastern Indonesia is prevalent, diverse and severe: implications for primaquine therapy against relapsing vivax malaria. PLoS Negl Trop Dis [Internet]. 2015 Mar 6 [cited 2025 Apr 9];9(3). Available from: https://pubmed.ncbi.nlm.nih.gov/25746733/

[4]Moiz B, Nasir A, Moatter T, Naqvi ZA, Khurshid M. Molecular characterization of glucose-6-phosphate dehydrogenase deficiency in Pakistani population. Int J Lab Hematol [Internet]. 2011 Dec [cited 2025 Apr 2];33(6):570–8. Available from: https://pubmed.ncbi.nlm.nih.gov/21507207/

[5]Abreha T, Hwang J, Thriemer K, Tadesse Y, Girma S, Melaku Z, et al. Comparison of artemether-lumefantrine and chloroquine with and without primaquine for the treatment of Plasmodium vivax infection in Ethiopia: A randomized controlled trial. PLoS Med. 2017 May 1;14(5).

[6]White NJ. Determinants of relapse periodicity in Plasmodium vivax malaria. Vol. 10, Malaria Journal. 2011.

[7]Boyer S, Doeurk B, Rakotonirina A, Chy S, Vong C, Piv E, et al. Anopheles mosquitoes in Mondulkiri forest, Cambodia: abundance, distribution, seasonal patterns and Plasmodium prevalence. Malar J [Internet]. 2025 Dec 1 [cited 2025 Apr 3];24(1):6. Available from: https://malariajournal.biomedcentral.com/articles/10.1186/s12936-024-05166-9

[8]Kazwaini M, Mading M, Litbang LP. Jenis Dan Status Anopheles Spp. Sebagai Vektor Potensial Malaria Di Pulau Sumba Provinsi Nusatenggara Timur. Indonesian Journal of Health Ecology [Internet]. 2014 [cited 2025 Apr 9];13(4):298–307. Available from: https://www.neliti.com/publications/81393/
[9]Klinkenberg E, Konradsen F, Herrel N, Mukhtar M, van der Hoek W, Amerasinghe FP. Malaria vectors in the changing environment of the southern Punjab, Pakistan. Trans R Soc Trop Med Hyg. 2004 Jul 1;98(7):442–9.
[10]Aisyah DN, Sitompul D, Diva H, Tirmizi SN, Hakim L, Surya A, et al. The Changing Incidence of Malaria in Indonesia: A 9-Year Analysis of Surveillance Data. Adv Public Health [Internet]. 2024 Jan 1 [cited 2025 Apr 9];2024(1):2703477. Available from: https://onlinelibrary.wiley.com/doi/full/10.1155/adph/2703477
[11]Malaria Technical Unit – Common Management Unit [Internet]. [cited 2025 Apr 2]. Available from: https://www.cmu.gov.pk/dmc-directorate-of-malaria-control/

Table S2: List of Ethics Review Boards and Regulatory Authorities

**Australia**	**The Human Research Ethics Committee of the Northern Territory Department of Health (HREC)**<br>John Mathews Building (Bldg 58) Royal<br>Darwin Hospital Campus, Rock<br>PO Box: 41096, Casuarina NT 0811, Australia<br>Website: www.menzies.edu.au
**United Kingdom**	**The Oxford Tropical Research Ethics Committee (OxTREC)**<br>University of Oxford<br>Research Services, University Offices<br>Willington Square, Oxford OX1 2JD<br>Tel: +44 (0) 1865 (2) 82106<br>E-mail: oxtrec@admin.ox.ac.uk<br>Website: www.admin.ox.ac.uk/rso
**Cambodia**	**National Ethics Committee for Health Research**, Ministry of Health, Kingdom of Cambodia<br>Lot #80, Samdach Penn Nouth Blvd (289), Sangkat Boeung Kok 2, Khan Tuol Kork, Phnom Penh, Cambodia.<br>Tel: (855~12) 842 442, (855-012) 528 789, (855-012) 203 382
**Ethiopia**	**The National Research Ethics Review Board (NRERB)**, Addis Ababa, Ethiopia<br>PO Box: 2490<br>Tel: +251 114-674-353<br>E-mail: repra@ethernet.edu.et<br>Fax: +251 114-660-241<br>Website: https://www.moe.gov.et/
	**Ethiopian Food and Drug Authority (EFDA)**, Addis Ababa, Ethiopia<br>Tel: 251-11-552 41 22/552 41 23<br>E-mail: clinicaltrial@efda.gov.et<br>Fax: 251-11-552 13 92<br>PO Box: 5681<br>Website: http://www.efda.gov.et/
**Indonesia**	**Health Research Ethical Committee**<br>Medical Faculty of Universitas Sumatera Utara/ H. Adam Malik General Hospital<br>Jl. Dr. Mansyur No 5 Medan, 20155 - Indonesia<br>Tel: +62-61-8211045; 8210555<br>Fax: +62-61-8216264<br>E-mail: komisietikfkusu@yahoo.com

	**Indonesian Food and Drug Agency (BPOM)**<br>Jl, Percetakan Negara No. 23 Jakarta Pusat 10560 Indonesia<br>E-mail: infopom@indo.net.id;<br>Tel: (021) 4244691, 4209221, 4263333, 4244755, 4241781, 4244819 Fax: (02) 4245139<br>Website: www.pom.go.id
**Pakistan**	**Ethics Review Committee**, Aga Khan University,<br>Stadium Road, P.O. Box 3500 Karachi 74800, Pakistan.<br>Tele: +92 21 3493 0051 Ext: 2447/4988.<br>Email: erc.pakistan@aku.edu
	**National Institutes of Health, Health Research Institute, National Bioethics Committee (NBC),**<br>Shahrah-e-Jamhuriat, Off Constitution Avenue, Sector G-5/2, Islamabad<br>Tel: 92-51-9224325, 9216793<br>Fax: 9216774<br>E-mail: nbcpakistan@nih.org.pk<br>Website: www.nbcpakistan.org.pk
	**Drug Regulatory Authority of Pakistan**<br>Prime Minister's National Health Complex<br>Park Road, Chak Shahzad, Islamabad<br>Toll Free: 0800-03727

Text S1: Database and Data Handling Procedures

Study data were initially collected on paper Case Report Forms (CRFs), which were checked for completeness against source documents before entry into the study database. Data were entered into a REDCap database (Research Electronic Data Capture), hosted and maintained by Menzies[1,2]. Access to the database was restricted to authorised personnel via unique user logins and passwords.

Data quality assurance was maintained through a combination of manual data review, automated range and consistency checks embedded in the database, and post-entry validation scripts run routinely by the data management team. All discrepancies were resolved via a formal data query process and logged in REDCap's audit trail. Regular data quality reports were generated to monitor site performance, and source data verification was conducted in line with the study monitoring plan. At study closeout, the final database was locked, and CRFs securely stored at each study site, and scanned copies saved on a secure server at Menzies. Identifiable participant data were not captured in the study database, and participants were tracked only through unique study IDs.

---

[1] PA Harris, R Taylor, R Thielke, J Payne, N Gonzalez, JG. Conde, Research electronic data capture (REDCap) – A metadata-driven methodology and workflow process for providing translational research informatics support, J Biomed Inform. 2009 Apr;42(2):377-81.

[2] PA Harris, R Taylor, BL Minor, V Elliott, M Fernandez, L O'Neal, L McLeod, G Delacqua, F Delacqua, J Kirby, SN Duda, REDCap Consortium, The REDCap consortium: Building an international community of software partners, J Biomed Inform. 2019 May 9 [doi: 10.1016/j.jbi.2019.103208]

Text S2: DSMB charter

EFFORT

Data Safety Monitoring Board (DSMB)

Charter

Effectiveness of novel approaches to radical cure with tafenoquine and primaquine (EFFORT) - a randomized controlled trial in *P. vivax* participants

V0.3

Principal Investigator: Dr Kamala Thriemer

DSMB members:

Chair: Associate Professor Laurens Manning
Infectious Diseases Physician
Senior Lecturer, Faculty of Health and Medical Sciences, Internal Medicine,
University of Western Australia

Professor Mavuto Mukaka
Head of Statistics, Mahidol-Oxford Tropical Medicine Research Unit, Bangkok, Thailand
Associate Professor, University of Oxford, UK
Honorary Professor, Malawi University of Science and Technology
Visiting Professor, University of Malawi

Dr Andre Machado Siqueira
Pesquisador / Médico Infectologista
Instituto Nacional de Infectologia Evandro Chagas – Fiocruz
Lab. Doenças Febris Agudas

Professor Esayas Kebede Gudina
MD, PhD, Internist
Institute of Health Sciences, Jimma University
Jimma, Ethiopia

Charter version	Date	Changes	Approved By
0.1	04/06/2020		
	22/09/2020	Addition of DSMB members	Kamala Ley-Thriemer
	02/10/2020	Addition of Professor Esayas Kebede Gudina from Ethiopia	Kamala Ley-Thriemer
	20/10/2020	First DSMB meeting: no changes	DSMB
0.2	19/04/2021	Second DSMB meeting: addition of all cause death to SAEs reported immediately. Concordance of hemolytic SAE definitions in DSMB charter and SOPs	DSMB
0.3	13/07/2023	Inclusion of interim analysis	DSMB

Introduction:

This Charter is for the Data and Safety Monitoring Board (DSMB) for the study titled "**Effectiveness of novel approaches to radical cure with tafenoquine and primaquine (EFFORT) - a randomized controlled trial in *P. vivax* participants**".

The Charter is intended to be a living document. The DSMB may wish to review it at regular intervals to determine whether any changes in procedure are needed.

Responsibilities of the DMSB:

The DSMB is responsible for safeguarding the interests of study participants, assessing predominantly the safety of study procedures, and for monitoring the overall conduct of the study.

The DMSB members are independent and it is their responsibility to prevent participants being exposed to any excess risks by recommending for the trial suspension or termination early if the safety or efficacy results are sufficiently convincing.

The responsibilities of the DSMB are:

- To determine how frequently interim reviews of trial data should be undertaken.

- To conduct interim safety data reviews
- To report (following each DSMB meeting) to the sponsor and to recommend whether the trial should continue, the protocol modified, or the trial be stopped.

Data review:

The following data will be sent to the DSMB for review as they occur

- Study Specific Haematolytic Serious Adverse Event. Those are defined as follows: -.
  - Haemoglobin of < 7g/dL PLUS macroscopic haemoglobinuria (Hillmen ≥ 5)
  - Anyone requiring a blood transfusion
  - Macroscopic haemoglobinuria (Hillmen ≥ 5) PLUS a fractional drop in haemoglobin ≥ 25% from baseline
- Other study specific SAEs defined as
  - Acute anaphylaxis
  - Persistent vomiting that requires hospitalisation for IV fluids
- All cause deaths
- Any SAEs which the investigator categorized as possibly or definitely-related to the study drug.

Quarterly summarised reports stratified by site will be sent to the DSMB and will include:

- All SAE reports
- Line listing of all Adverse Events stratified by severity and study arm
- Flow diagram with the number of screened and enrolled participants, participants who have been withdrawn or lost to follow up
- Line listing of all protocol violations
- Individual participant Hb data between Day 0 and Day 14 as available
- Number of participants with fall in Hb greater than 25% from baseline within the first 21 days or reporting macroscopic haemoglobinuria

Procedures for DSMB Meetings:

Meeting Schedule

The DSMB will hold meetings as follows:

- Online meeting before the study start
- Data review by email every 3 months
- Data review by email 1 month after the last participant recruitment
- Additional online meetings as well as additional data review by email can be requested by the DSMB at their discretion

Meeting format

The first DSMB meeting will be held by teleconference call. Consecutive data review will be done via email. The DSMB can hold additional meetings / teleconferences as it requires. The first DSMB teleconference will include the study PI.

Scope of the review data

This study will generate data on participant inclusion, exclusion, clinical, biochemical, haematological and G6PD deficiency data. The DSMB can request additional clinical data on participants if needed for their review.

Study stopping rules

There are no predetermined stopping rules for this study. The DSMB has the right to recommend that the study be stopped if this is the considered view of the DSMB.

Interim analysis

An interim analysis has been added to the protocol (V3.2) In 2023. **The interim analysis was added because of the protracted start of different study sites to potentially enable analysis of three sites only.**

The interim analysis will be conducted at the availability of 6 months of follow-up data for 50% of the original sample size (i.e., 360 participants, which is 50% of 720 participants). The timing of this interim analysis is expected to be mid 2023, which is approximately 2 years after the first patient was recruited. At this point, the study will be continuing to enrol participants.

An independent Statistician will perform the interim analysis and prepare a report for the DSMB. The DSMB will then issue a recommendation to the study PI based on the following:

- The interim analysis will be conducted for the primary effectiveness outcome and the first secondary effectiveness outcome.
- The interim analysis will allow stopping for superiority (of the primary outcome) early using a conservative Haybittle-Peto boundary with early stopping to reject the null hypothesis.
- If the decision is made to stop the trial early, recruitment will cease in Indonesia and Ethiopia (noting that Cambodia has finished recruitment at the time of the interim analysis) but will continue in Pakistan.
- By stopping the trial early, the results from the three original sites (Cambodia, Indonesia and Ethiopia) will be made available without waiting for completion of recruitment and follow-up in Pakistan. Upon completion at the study site in Pakistan those results will then be combined with the three sites using a meta-analysis approach.

No other aspects of the trial will be adapted or revised based on the results of the interim analysis, and the results of the interim analysis will not be used to adjust the sample size of this trial.

Confidentiality

All DSMB discussions about this study must be kept confidential.

Independence

Members of the DSMB must be free of conflicts of interest such as likely financial gain from the results of this study. If a DSMB member has a conflict of interest, he/she should declare and withdraw from the service of the DSMB.

Quorum

There are 4 DSMB members. All members should be present for teleconferences or meetings.

Responsibilities of the Principal Investigator:

The Principal Investigator has overall responsibility for study conduct and for maintaining good communication with the DSMB.

Table S3: Protocol deviations and violations

Findings	Reporting date	Date resolved	Action taken	Type of protocol violation/deviation
Venous blood was collected instead of capillary samples on day 7 and 14 follow-up visits on 21 patients	4th July 2022	5th July 2022	Staff refresher training	Protocol Deviation
Patient <18 years was enrolled.	2nd June 2023	18th June 2023	The participant will remain in the study and to be followed up until they exit the study, no study medication will be administered in case they have a recurrence.	Protocol Violation
Sample collection was not done at an unscheduled visit.	12th July 2023	12th July 2023	PI provided comprehensive training on follow-up visits.	
The participant was randomized to the PQ7 arm, weight 40kgs, and received 4 tablets o PQ instead of 3 tablets.	8th July 2023	10th July 2023	Participant was followed up after two days and the dose was adjusted. Retraining on using dosing chart was done.	Protocol Violation
Participant had a Hb drop from 18.4 at enrolment to 8.4 on D3, the hemolysis management protocol was not followed. Subsequent Hb on D7 was 15.4g/dl, D14 was 14.3g/dl and D21 was 11.9g/dl.	13/2/2024	15/2/2024	The site team informed to always compare the baseline Hb to subsequent follow-up visits.	Protocol Violation

Text S3: Patient or public involvement

Design

Public involvement in the design of the trial was primarily achieved through ongoing engagement with National Malaria Control Programs (NMCPs). These programs consistently emphasized the need to generate evidence on the effectiveness of novel radical cure options[3], and our trial was developed in direct response to these priorities. In the absence of formal patient groups in the study settings, qualitative data on patient experiences and treatment challenges provided important insights, highlighting the urgent need for more effective radical cure options.

Conduct

In the absence of formal patient groups in the study settings, trial sites relied on alternative community engagement mechanisms. These included informal engagement in some sites and more formalized engagement through community advisory boards or comparable structures, which served to support the study teams in the ethical and culturally appropriate conduct of the trial.

Reporting

Patient and public involvement in the reporting of the trial was guided by evidence on participant preferences for receiving study results. These preferences were systematically assessed within the trial[4] and informed the design of dissemination activities. Based on these findings, dedicated patient dissemination meetings were conducted. The evaluation of these activities is ongoing and will be published separately. In parallel, stakeholder meetings were organized with policymakers and other key stakeholders at both national and international levels, with reports from these consultations to be prepared and disseminated separately.

[3] Ruwanpura VSH, Nowak S, Gerth-Guyette E, Theodora M, Dysoley L, Haile M, Peeters Grietens K, Price RN, Lynch CA, Thriemer K. Further evidence needed to change policy for the safe and effective radical cure of vivax malaria: Insights from the 2019 annual APMEN Vivax Working Group meeting. Asia Pac Policy Stud. 2021 May;8(2):208-242. doi: 10.1002/app5.314. Epub 2021 Jan 12.

[4] Bamboro SA, Jabbar FA, Bagita-Vangana M, Hasibuan N, Degaga TS, Ghanchi N, Beg MA, Tripura R, Pitaloka AP, Tego TT, Safitri W, Yulita, Cassidy-Seyoum S, Mwaura M, Mnjala H, Lee G, Dysoley L, von Seidlein L, Price RN, Unger HW, Adhikari B, Thriemer K. How do study participants want to be informed about study results: Findings from a malaria trial in Cambodia, Ethiopia, Pakistan, and Indonesia. J Clin Transl Sci. 2025 Mar 27;9(1):e83. doi: 10.1017/cts.2025.56. eCollection 2025.

Table S4: CONSORT 2025 checklist item description

Section/topic	No	CONSORT 2025 checklist item description	Reported on page no.
**Title and abstract**			
Title and structured abstract	1a	Identification as a randomised trial	1
	1b	Structured summary of the trial design, methods, results, and conclusions	4-5
**Open science**			
Trial registration	2	Name of trial registry, identifying number (with URL) and date of registration	4
Protocol and statistical analysis plan	3	Where the trial protocol and statistical analysis plan can be accessed	Included in supplement
Data sharing	4	Where and how the individual de-identified participant data (including data dictionary), statistical code and any other materials can be accessed	25
Funding and conflicts of interest	5a	Sources of funding and other support (eg, supply of drugs), and role of funders in the design, conduct, analysis and reporting of the trial	26
	5b	Financial and other conflicts of interest of the manuscript authors	25
**Introduction**			
Background and rationale	6	Scientific background and rationale	8-9
Objectives	7	Specific objectives related to benefits and harms	9
**Methods**			
Patient and public involvement	8	Details of patient or public involvement in the design, conduct and reporting of the trial	Included in supplement
Trial design	9	Description of trial design including type of trial (eg, parallel group, crossover), allocation ratio, and framework (eg, superiority, equivalence, non-inferiority, exploratory)	10
Changes to trial protocol	10	Important changes to the trial after it commenced including any outcomes or analyses that were not prespecified, with reason	NA
Trial setting	11	Settings (eg, community, hospital) and locations (eg, countries, sites) where the trial was conducted	
Eligibility criteria	12a	Eligibility criteria for participants	10
	12b	If applicable, eligibility criteria for sites and for individuals delivering the interventions (eg, surgeons, physiotherapists)	NA
Intervention and comparator	13	Intervention and comparator with sufficient details to allow replication. If relevant, where additional materials describing the intervention and comparator (eg, intervention manual) can be accessed	10 and supplement with dosing tables
Outcomes	14	Prespecified primary and secondary outcomes, including the specific measurement variable (eg, systolic blood pressure), analysis metric (eg, change from baseline, final value, time to	12-13

		event), method of aggregation (eg, median, proportion), and time point for each outcome	
Harms	15	How harms were defined and assessed (eg, systematically, non-systematically)	12-13
Sample size	16a	How sample size was determined, including all assumptions supporting the sample size calculation	13
	16b	Explanation of any interim analyses and stopping guidelines	14
Randomisation:			
Sequence generation	17a	Who generated the random allocation sequence and the method used	11
	17b	Type of randomisation and details of any restriction (eg, stratification, blocking and block size)	11
Allocation concealment mechanism	18	Mechanism used to implement the random allocation sequence (eg, central computer/telephone; sequentially numbered, opaque, sealed containers), describing any steps to conceal the sequence until interventions were assigned	11
Implementation	19	Whether the personnel who enrolled and those who assigned participants to the interventions had access to the random allocation sequence	11
Blinding	20a	Who was blinded after assignment to interventions (eg, participants, care providers, outcome assessors, data analysts)	NA
	20b	If blinded, how blinding was achieved and description of the similarity of interventions	NA
Statistical methods	21a	Statistical methods used to compare groups for primary and secondary outcomes, including harms	13
	21b	Definition of who is included in each analysis (eg, all randomised participants), and in which group	13
	21c	How missing data were handled in the analysis	SAP in supplement
	21d	Methods for any additional analyses (eg, subgroup and sensitivity analyses), distinguishing prespecified from post hoc	13-14
**Results**			
Participant flow, including flow diagram	22a	For each group, the numbers of participants who were randomly assigned, received intended intervention, and were analysed for the primary outcome	Fig 1
	22b	For each group, losses and exclusions after randomisation, together with reasons	15
Recruitment	23a	Dates defining the periods of recruitment and follow-up for outcomes of benefits and harms	14
	23b	If relevant, why the trial ended or was stopped	NA
Intervention and comparator delivery	24a	Intervention and comparator as they were actually administered (eg, where appropriate, who delivered the intervention/comparator, how participants adhered, whether they were delivered as intended (fidelity))	NA
	24b	Concomitant care received during the trial for each group	NA
Baseline data	25	A table showing baseline demographic and clinical characteristics for each group	Table 1
Numbers analysed,	26	For each primary and secondary outcome, by group:<br>● the number of participants included in the analysis	Table 2-3 and Fig 2-3

outcomes and estimation		● the number of participants with available data at the outcome time point<br>● result for each group, and the estimated effect size and its precision (such as 95% confidence interval)<br>● for binary outcomes, presentation of both absolute and relative effect size	
Harms	27	All harms or unintended events in each group	Table 3
Ancillary analyses	28	Any other analyses performed, including subgroup and sensitivity analyses, distinguishing pre-specified from post hoc	16-17
**Discussion**			
Interpretation	29	Interpretation consistent with results, balancing benefits and harms, and considering other relevant evidence	19-24
Limitations	30	Trial limitations, addressing sources of potential bias, imprecision, generalisability, and, if relevant, multiplicity of analyses	23

Table S5: CONSORT Harms 2022 integrated into CONSORT 2010 items checklist

Section/Topic	Item No	Checklist item	Reported on page No
**Title and abstract**			
	1a	Identification as a randomised trial in the title	1
	1b	Structured summary of trial design, methods, results of outcomes of benefits and harms, and conclusions (for specific guidance see CONSORT for abstracts)	4-5
**Introduction**			
Background and objectives	2a	Scientific background and explanation of rationale	8-9
	2b	Specific objectives or hypotheses for outcomes benefits and harms	9
**Methods**			
Trial design	3a	Description of trial design (such as parallel, factorial) including allocation ratio	10
	3b	Important changes to methods after trial commencement (such as eligibility criteria), with reasons	NA
Participants	4a	Eligibility criteria for participants	10
	4b	Settings and locations where the data were collected	10
Interventions	5	The interventions for each group with sufficient details to allow replication, including how and when they were actually administered	10 and supplement with dosing tables
Outcomes	6a	Completely defined pre-specified primary and secondary outcome measures for both benefits and harms, including how and when they were assessed	12-13
	6b	Any changes to trial outcomes after the trial commenced, with reasons	NA
	6c	Describe if and how non-prespecified outcomes of benefits and harms were identified, including any selection criteria, if applicable	14
Sample size	7a	How sample size was determined	13
	7b	When applicable, explanation of any interim analyses and stopping guidelines	14

Section/Topic	Item No	Checklist item	Reported on page No
Randomisation:			
Sequence generation	8a	Method used to generate the random allocation sequence	11
	8b	Type of randomisation; details of any restriction (such as blocking and block size)	11
Allocation concealment mechanism	9	Mechanism used to implement the random allocation sequence (such as sequentially numbered containers), describing any steps taken to conceal the sequence until interventions were assigned	11
Implementation	10	Who generated the random allocation sequence, who enrolled participants, and who assigned participants to interventions	11
Blinding	11a	If done, who was blinded after assignment to interventions (e.g., participants, care providers, those assessing outcomes of benefits and harms) and how	NA
	11b	If relevant, description of the similarity of interventions	NA
Statistical methods	12a	Statistical methods used to compare groups for primary and secondary outcomes of both benefits and harms	13
	12b	Methods for additional analyses, such as subgroup analyses and adjusted analyses	13-14
**Results**			
Participant flow (a diagram is strongly recommended)	13a	For each group, the numbers of participants who were randomly assigned, received intended treatment, and were analysed for outcomes of benefits and harms	Fig 1
	13b	For each group, losses and exclusions after randomisation, together with reasons	15
Recruitment	14a	Dates defining the periods of recruitment and follow-up for outcomes of benefits and harms	14
	14b	Why the trial ended or was stopped	NA
Baseline data	15	A table showing baseline demographic and clinical characteristics for each group	Table 1
Numbers analysed	16	For each group, number of participants (denominator) included in each analysis and whether the analysis was by original assigned groups and if any exclusions were made	In each table and fig
Outcomes and estimation	17a	For each primary and secondary outcome of benefits and harms, results for each group, and the estimated effect size and its precision (such as 95% confidence interval)	15 onwards and table 1-3, fig 1-3
	17a2	For outcomes omitted from the trial report (benefits and harms), provide rationale for not reporting and indicate where the data on omitted outcomes can be accessed	No outcomes were omitted
	17b	Presentation of both absolute and relative effect sizes is recommended, for outcomes of benefits and harms	19

Section/Topic	Item No	Checklist item	Reported on page No
	17c	Report zero events if no harms were observed	18-19
Ancillary analyses	18	Results of any other analyses performed, including subgroup analyses and adjusted analyses, distinguishing pre-specified from exploratory	16-17
Harms	19	All important harms or unintended effects in each group (for specific guidance see CONSORT for harms)	Table 3 and page 18
**Discussion**			
Limitations	20	Trial limitations, addressing sources of potential bias related to the approach to collecting or reporting data on harms, imprecision, and, if relevant, multiplicity or selection of analyses	23
Generalisability	21	Generalisability (external validity, applicability) of the trial findings	19-24
Interpretation	22	Interpretation consistent with results, balancing benefits and harms, and considering other relevant evidence	19-24
**Other information**			
Registration	23	Registration number and name of trial registry	Abstract and page 10
Protocol	24	Where the full trial protocol and other relevant documents can be accessed, including additional data on harms	Protocol as supplement
Funding	25	Sources of funding and other support (such as supply of drugs), role of funders	14

Table S6: CONSORT extension for abstracts

Item	Description	Reported on line number
Title	Identification of the study as randomized	65
Authors *	Contact details for the corresponding author	n/a
Trial design	Description of the trial design (e.g. parallel, cluster, non-inferiority)	65
Methods		
Participants	Eligibility criteria for participants and the settings where the data were collected	66-68
Interventions	Interventions intended for each group	71-72
Objective	Specific objective or hypothesis	62-63
Outcome	Clearly defined primary outcome for this report	75-77
Randomization	How participants were allocated to interventions	70
Blinding (masking)	Whether or not participants, care givers, and those assessing the outcomes were blinded to group assignment	65
Results		
Numbers randomized	Number of participants randomized to each group	79-80
Recruitment	Trial status	79
Numbers analysed	Number of participants analysed in each group	80
Outcome	For the primary outcome, a result for each group and the estimated effect size and its precision	80-85
Harms	Important adverse events or side effects	87-90
Conclusions	General interpretation of the results	92-95
Trial registration	Registration number and name of trial register	77
Funding	Source of funding	101-102

**this item is specific to conference abstracts*

Table S7: Site-specific adjusted male median of G6PD activity

Country	Site	100% G6PD activity (adjusted male median) U/gHb[5](IQR)	70% G6PD activity U/gHb	G6PD activity threshold used for recruitment U/gHb
Cambodia	Kravan	6.95 (6.1-7.9)	4.865	5.0
	Siem Pang	7.73 (6.6-8.3)	5.405	5.5
	Chombak	7.55 (6.5-9.6)	5.285	5.0
Ethiopia*	Arba Minch	7.58 (6.5-8.2)	5.302	5.5
Indonesia	Batubara, Sumatra	6.45 (5.9-7.4)	4.515	5.0
Pakistan	Karachi	7.25 (6.4-8.2)	5.075	5.1
	Thatta	7.55 (6.5-8.2)	5.285	5.5

G6PD activity was measured by STANDARD G6PD. Rather than using the manufacturer recommended cut-off of 6U/gHb to define G6PD normal status, the site specific adjusted male median (AMM) was calculated and patients with ≥70% activity of the AMM at time of presentation were considered eligible to be recruited into the trial. In order to determine 100% G6PD activity in each site, a total of 30 non-related adult males attending the health facility were sampled at each site before study start.

*Established for a previous study [6]

[5] Domingo GJ, Satyagraha AW, Anvikar A, Baird K, Bancone G, Bansil P, Carter N, Cheng Q, Culpepper J, Eziefula C, Fukuda M, Green J, Hwang J, Lacerda M, McGray S, Menard D, Nosten F, Nuchprayoon I, Oo NN, Bualombai P, Pumpradit W, Qian K, Recht J, Roca A, Satimai W, Sovannaroth S, Vestergaard LS, Von Seidlein L. G6PD testing in support of treatment and elimination of malaria: recommendations for evaluation of G6PD tests. Malar J. 2013 Nov 4;12:391. doi: 10.1186/1475-2875-12-391.

[6] Thriemer K, Degaga TS, Christian M, Alam MS, Rajasekhar M, Ley B, Hossain MS, Kibria MG, Tego TT, Abate DT, Weston S, Mnjala H, Rumaseb A, Satyagraha AW, Sadhewa A, Panggalo LV, Ekawati LL, Lee G, Anose RT, Kiros FG, Simpson JA, Karahalios A, Woyessa A, Baird JK, Sutanto I, Hailu A, Price RN. Primaquine radical cure in patients with Plasmodium falciparum malaria in areas co-endemic for P falciparum and Plasmodium vivax (PRIMA): a multicentre, open-label, superiority randomised controlled trial. Lancet. 2023 Dec 2;402(10417):2101-2110. doi: 10.1016/S0140-6736(23)01553-2.

Text S4: Methodological details on randomisation

Generation of the randomisation list was done by a statistician at the University of Melbourne who was not involved in this study's design, conduct or analysis. They are not an author of this manuscript nor were they involved in the study in any way. The randomisation was done using the ralloc package in Stata v16.1 using randomly permuted blocks (in size and order) in block sizes that were multiples of 3, stratified by study site in a 1:1:1 ratio across the study arms. The generated lists were emailed to an independent person at Menzies School of Health Research, who made them available to the investigators at each site in sealed opaque envelopes. This method was used as a stable internet connection at every study site could not be relied upon at the time the study was conducted. The randomisation lists were stored on a password protected server at the University of Melbourne, with restricted access. No auditing was performed of the randomisation access.

Table S8a: Dosing chart for patients in the 14-day-low-dose primaquine arm

Patient weight in kg	Number of tablets per dose (using 15mg formulation)	Number of tablets per dose (using 7.5mg formulation)	Dose (mg/kg/day)	Total Dose (mg/kg)
>35 and ≤45	0.5	1	0.17-0.21	2.33-2.94
>45 and ≤70	1	2	0.21-0.33	3.00-4.67
>70	1.5	3	<0.32	<4.50

Table S8b: Dosing chart for patients in the 7-day-high-dose primaquine arm

Patient weight in kg	Number of tablets per dose (using 15mg formulation)	Number of tablets per dose (using 7.5mg formulation)	Dose (mg/kg/day)	Total Dose (mg/kg)
>35 and ≤45	3	6	1.02-1.29	7.46-9.00
>45 and ≤70	4	8	0.86-1.33	6.00-9.33
>70	6	12	<1.27	<8.87

Table S8c: Dosing chart for patients in the Tafenoquine arm

Patient weight in kg	Number of tablets per dose (using 100mg formulation)	Total Dose (mg/kg)
>35 and ≤45	3	6.67-8.57
>45 and ≤70	3	4.3-6.6
>70	3	<4.2

Table S8d: Dosing chart for chloroquine for patients recruited in Ethiopia and Pakistan

Patient weight in kg	Number of tablets per day (using 150mg base formulation)			Total dose in mg/kg
	Day 0	Day 1	Day 2	
> 36 and ≤50	3	2	2	21.0-29.2
>50	4	4	2	<30.0

Table S8e: Dosing chart for DHA-Piperaquine for patients recruited in Indonesia

**Patient weight in kg**	**Number of tablets per dose (using formulation containing 40 mg of DHA and 320 mg piperaquine)**	**DHA (mg/kg) per dose**	**Piperaquine (mg/kg) per dose**
>31 and ≤40	2	2.0-2.5	16.0-20.6
>40 and ≤60	3	2.0-2.9	16.3-23.4
>60 and ≤80	4	2.0-2.7	16.0-21.3
>80	5	<2.5	>20

DHA - dihydroartemisinin

Table S8f: Dosing chart for artesunate-pyronaridine for patients recruited in Cambodia

Patient weight in kg	Number of Tablets per dose (using formulation containing 60mg of artesunate and 180mg of pyronaridine tetraphosphate)	Artesunate (mg/kg) per dose	Pyronaridine (mg/kg) per dose
>45 and ≤65	3	2.7-4.0	8.3 – 12.0
>65	4	>3.7	8.0 – 11.1

Text 5: Parasite genotyping and recurrence classification

Parasite genotyping and variant calling

In a post-hoc analysis, parasite genotyping was undertaken on all PCR-confirmed day 0 and day of failure clinical samples using a previously described 93-microhaplotype marker panel[7]. Briefly, genotyping was conducted using a rhAmpSeq (Integrated DNA Technologies) workflow to amplify the marker regions, followed by Illumina sequencing, generating 150bp paired end reads. The NextSeq platform was used for the Cambodian samples, MiniSeq and NextSeq for Ethiopia, and MiniSeq for Pakistan. Positive and negative controls were included for quality assurance. Variant calling was conducted using the vivaxGEN microhaplotype-calling pipeline (https://github.com/vivaxgen/MicroHaps)[3]. To reduce potential artifacts, alleles making up less than 2% of the total read pairs per marker for each sample are excluded, followed by removal of alleles with less than 5 read pairs per sample. Samples with less than 75% (70) successfully genotyped microhaplotype markers were excluded from further analysis to ensure high accuracy in relatedness estimations between infection pairs.

Recurrence classification

The relatedness between successfully genotyped pairs of day 0 and day of failure clinical samples was measured using identity-by-descent (IBD) with Dcifer software[8]. On the basis that a pair of sporozoites has 4 parental gametes, a minimum threshold of 25% IBD was applied to define pairs of day 0 and day of failure infections as homologous pairs[9]. The assumption is that homologous pairs of infections have higher probability of deriving from a single inoculation than from multiple inoculations and, thus, are more likely to be relapses than reinfections. Assessment of the IBD distributions in independent (day 0 only) clinical samples revealed that the IBD≥25% threshold should omit majority of independent infections (suspected reinfections) (Figure S3). However, in Cambodia and Indonesia, a high proportion of independent (day 0) infections have IBD≥25% to one another, and hence the risk of defining highly related reinfections as suspected relapses will be greater than in Ethiopia and Pakistan. However, it should be noted that the high baseline relatedness in Cambodia and Indonesia (Sumatra) may reflect a modest degree of inbreeding in these populations resulting from low transmission and associated out-crossing; as such, the risk of reinfection and thus misclassification error in these low endemic settings may be low.

---

[7] Kleinecke M, Sutanto E, Rumaseb A, Hoon KS, Trimarsanto H, Osborne A, Manrique P, Peters T, Hawkes D, Benavente ED, Whitton G, Siegel SV, Pearson RD, Amato R, Rai A, Nhien NTT, Nguyen HC, Assefa A, Degaga TS, Abate DT, Rahim AG, Pasaribu AP, Sutanto I, Alam MS, Pava Z, Lopera-Mesa T, Echeverry D, William T, Anstey NM, Grigg MJ, Day NP, White NJ, Kwiatkowski DP, Taylor AR, Noviyanti R, Neafsey D, Price RN, Auburn S. Microhaplotype deep sequencing assays to capture Plasmodium vivax infection lineages. Nat Commun. 2025 Aug 5;16(1):7192. doi: 10.1038/s41467-025-62357-x.

[8] Gerlovina I, Gerlovin B, Rodríguez-Barraquer I, Greenhouse B. Dcifer: an IBD-based method to calculate genetic distance between polyclonal infections. Genetics. 2022 Sep 30;222(2):iyac126. doi: 10.1093/genetics/iyac126.

[9] Taylor AR, Watson JA, Chu CS, Puaprasert K, Duanguppama J, Day NPJ, Nosten F, Neafsey DE, Buckee CO, Imwong M, White NJ. Resolving the cause of recurrent Plasmodium vivax malaria probabilistically. Nat Commun. 2019 Dec 6;10(1):5595. doi: 10.1038/s41467-019-13412-x.

Table S9: DELTA2 recommended reporting items for the sample size calculation of a randomised controlled trial with a superiority question

Recommended reporting items	Page and line numbers where item is reported
Core items	
(1) Primary outcome (and any other outcome on which the calculation is based)	Page 13
If a primary outcome is not used as the basis for the sample size calculation, state why	
(2) Statistical significance level and power	Page 14
(3) Express the target difference according to outcome type	
(a) Binary—state the target difference as an absolute or relative effect (or both), along with the intervention and control group proportions. If both an absolute and a relative difference are provided, clarify if either takes primacy in terms of the sample size calculation	Page 14
(b) Continuous—state the target mean difference on the natural scale, common standard deviation, and standardised effect size (mean difference divided by the standard deviation)	N/A
(c) Time-to-event—state the target difference as an absolute or relative difference (or both); provide the control group event proportion, planned length of follow-up, intervention and control group survival distributions, and accrual time (if assumptions regarding them are made). If both an absolute and relative difference are provided for a particular time point, clarify if either takes primacy in terms of the sample size calculation	N/A
(4) Allocation ratio	Page 14
If an unequal ratio is used, the reason for this should be stated	
(5) Sample size based on the assumptions as per above	Page 14
(a) Reference the formula/sample size calculation approach, if standard binary, continuous, or survival outcome formulas are not used. For a timeto-event outcome, the number of events required should be stated	
(b) If any adjustments (eg, allowance for loss to follow-up, multiple testing) that alter the required sample size are incorporated, they should also be specified, referenced, and justified along with the final sample size	Page 14

(c) For alternative designs, additional input should be stated and justified. For example, for a cluster randomised controlled trial (or an individually randomised controlled trial with clustering), state the average cluster size and intracluster correlation coe(cient(s). Variability in cluster size should be considered and, if necessary, the coe(cient of variation should be incorporated into the sample size calculation. Justification for the values chosen should be given	
(d) Provide details of any assessment of the sensitivity of the sample size to the inputs used	
Additional items for grant application and trial protocol	
()) Underlying basis used for specifying the target difference (an important or realistic difference)	
(7) Explain the choice of target difference—specify and reference any formal method used or relevant previous research	
Additional item for trial results paper	
(8) Reference the trial protocol	Submitted with manuscript
This set of reporting items has been developed with the conventional statistical (Neyman-Pearson) approach to a sample size calculation in mind. Some of the reporting items would differ if another approach were to be used. This checklist has been taken from table 1 in *BMJ* 2,18;3)3:k375,, as a standalone document for readers to print out or fill in electronically.	

Text S6: Statistical Analysis Plan

“Effectiveness of novel approaches to radical cure with tafenoquine and primaquine (EFFORT) - a randomized controlled trial in P. vivax participants”

EFFORT

Version number: 2.0

Date: 28th March 2023

1. Introduction

This Statistical Analysis Plan (SAP) provides a detailed and comprehensive description of the main pre-planned analyses for the clinical trial "Effectiveness of novel approaches to radical cure with tafenoquine and primaquine (EFFORT) - a randomized controlled trial in *Plasmodium* vivax participants".

The main purpose of the trial is to assess the comparative effectiveness of a short-course of high dose primaquine (total dose 7 mg/kg given unsupervised over 7 days) and tafenoquine (single dose of 300 mg) compared to the current standard low dose (total dose 3.5 mg/kg given unsupervised over 14 days) in preventing *Plasmodium vivax* relapse in G6PD normal patients. The study conduct is registered with ClinicalTrials.gov under NCT04411836.

This document describes the statistical methods for the primary and secondary outcomes of the trial as defined by the protocol.

2. Study design and objectives

2.1. Summary of trial design

This is a multicentred open label randomised controlled effectiveness study in G6PD normal (≥70% activity) patients with uncomplicated vivax malaria. Patients presenting to a participating treatment centre with uncomplicated vivax malaria and fulfilling the enrolment criteria will be randomly assigned to one of three treatment arms:

- The control arm: patients are treated with schizontocidal treatment plus low dose PQ (total dose 3.5mg/kg) unsupervised over 14 days (PQ14)
- The first intervention arm: patients are treated with schizontocidal treatment plus high dose PQ (total dose 7 mg/kg) unsupervised over 7 days (PQ7)
- The second intervention arm: patients are treated with schizontocidal treatment plus a single dose (300 mg) of Tafenoquine (TQ).

All patients will receive standard medical care for the management of uncomplicated malaria, with blood schizontocidal treatment administered as either chloroquine (total dose 25 mg base/kg) or an artemisinin combination therapy such as a three-dose regimen of dihydroartemisinin-piperaquine, or pyronaridine and artesunate (Pyramax), depending on local recommendations and known chloroquine efficacy.

To minimise observer bias, patients will be reviewed only once after enrolment during their course of treatment. A clinical review will be carried out on day 3 (±1 day) to assess whether the patient has persistent peripheral parasitaemia, ensure clinical recovery, and assess for adverse drug reactions.

After day 3, patients in the TQ arm will be reviewed on day 7 and 14 to increase safety monitoring. Patients in the PQ arms (i.e., PQ7 and PQ14) will not be visited on these days to ensure limited

interaction as not to bias adherence. Afterwards, all patients will be reviewed weekly from day 21 until day 42 (±2 days) and thereafter monthly until month 6 (±1 week). At each visit a peripheral blood film will be taken, but this will only be read immediately if the patient has symptoms suggestive of malaria.

Patients presenting with a recurrent vivax malaria episode before day 28 will be treated with rescue treatment as per national guidelines. Patients presenting with a recurrent vivax malaria episode at or after day 28 will be treated with the same treatment as they were allocated to at enrolment. Patients presenting with *falciparum malaria* during follow up will be treated per national guidelines.

2.2.Objectives

- To assess the effectiveness of a short-course of high dose primaquine (total dose 7mg/kg given unsupervised over 7 days) compared to the current standard low dose primaquine regimen (total dose 3.5mg/kg given unsupervised over 14 days).
- To assess the effectiveness of tafenoquine (single dose of 300mg) compared to the short-course high dose primaquine regimen.
- To assess the effectiveness of tafenoquine (single dose of 300mg) compared to the standard low dose primaquine regimen.
- To assess the safety of tafenoquine compared to the high and low dose primaquine regimens.
- To assess the cost-effectiveness and feasibility of high dose primaquine and tafenoquine compared to the current low dose primaquine regimen. Note that this objective is not covered in this statistical analysis plan.

2.3.Endpoints

2.3.1. Effectiveness Endpoints

2.3.1.1.Primary Effectiveness Endpoint

The primary effectiveness outcome will be the incidence risk (time to first event) of any *P. vivax* parasitaemia during the 6-month follow up period as determined by microscopy compared between the PQ7 arm and the control arm (PQ14).

2.3.1.2.Secondary Effectiveness Endpoints

The incidence risk (time to first event) of any *P. vivax* parasitaemia during the 6 months follow up period as determined by microscopy with the following comparisons:

- PQ7 and TQ

- PQ14 and TQ

The incidence risk (time to first event) of symptomatic *P. vivax* parasitaemia during the 6-month follow up period as determined by microscopy with the following comparisons:

- PQ7 and the control arm (PQ14)
- PQ7 and TQ
- PQ14 and TQ

The incidence rate (events per person-time) of symptomatic *P. vivax* parasitaemia during the 6 months follow up period as determined by microscopy with the following comparisons:

- PQ7 and PQ14
- PQ7 and TQ
- PQ14 and TQ

The incidence rate (events per person-time) of any *P. vivax* parasitaemia during the 6 months follow up period as determined by microscopy with the following comparisons:

- PQ7 and PQ14
- PQ7 and TQ
- PQ14 and TQ

2.3.2. Safety Endpoints

- The incidence risk of severe (Hb <5g/dl) or moderate (≥5g/dl and <7g/dl) anaemia within 3 days of starting treatment and/or requiring blood transfusion within the 6 month follow up period.
- The incidence risk of an acute drop in Hb of >25% to <7g/dl within 3 days of starting treatment.
- The incidence risk of an acute drop in Hb of >5g/dl within 3 days of starting treatment in all three arms and the risk of an acute drop in Hb of >5g/dl on day 7 in the TQ arm only.
- The number and proportion of patients for whom PQ was discontinued or stopped after review on day 3.
- The number and proportion of gastro-intestinal symptoms reported in each arm.
- The number and proportion of neuropsychiatric adverse events in the TQ arm.
- The number and proportion of adverse and serious adverse events in each arm within 42 days after start of treatment.

2.4.Sample size

A sample size of 720 participants (240 in each arm and allowing for 10% lost to follow-up) will have 95% power to detect a reduction in the risk of recurrence of *P. vivax* parasitaemia (over 6 months follow-up), from 36% in the PQ14 arm to 20% in the PQ7 arm with two-sided alpha of

0.025. This sample size will provide 80% power to detect a reduction in the risk of recurrence of *P. vivax* parasitaemia from 33% in the TQ arm to 20% in the PQ7 arm (secondary outcome #1). A two-sided alpha of 0.025 was chosen to penalize for the multiple testing inherent in a 3-arm trial.

The following information was used to inform the sample size:

- Previous RCTs of efficacy (i.e. supervised treatment) with a 14-day low dose treatment (control arm) of PQ found the risk of recurrence at 6 months of 20% in Ethiopia (1) and 27% in a multicenter study (2). Supervised treatment of high dose PQ given over 7 days reduces the risk of recurrence to 10% (3). Efficacy data for TQ found a risk of recurrence of 33% after 6 months (2).
- Previous RCT of effectiveness (i.e., unsupervised treatment) of low dose PQ showed an increased risk of recurrence of 36% at 6 months(1). No data are yet available on the effectiveness of high dose 7-day courses of PQ but we assume a similar (conservative) increased risk to approximately 20%. We assume that effectiveness in the TQ arm is equivalent to its efficacy as it is provided as a single dose (i.e., 33%).

Using data from the study conducted in Ethiopia (1) and the IMPROV study (3), this sample size will have 98% power to detect a change in secondary effectiveness outcome (i.e. a change in the incidence rate of any recurrent *P. vivax* from 0.25 episodes per 6 months for PQ14 to 0.09 episodes per 6 months in the PQ7) with a two-sided alpha of 0.025.

We will aim to recruit 220-250 patients at the Cambodian sites, 130-150 in Indonesia and 350 in Ethiopia, to account for the different burden of malaria and recruitment rates.

An additional site in Pakistan has been included to improve generalisability of the study. We anticipate that the additional site will recruit 240 participants, which will increase power to more than 98% to detect a reduction in the risk of recurrence of *P. vivax* parasitaemia from 36% in the PQ14 arm to 20% in the PQ7 arm with a two-sided alpha of 0.0245 and allowing for 10% loss to follow-up. This sample size will also provide more than 90% power to detect a reduction in the risk of recurrence of *P. vivax* parasitaemia from 33% in the TQ arm to 20% in the PQ7 arm (secondary outcome #1). A two-sided alpha of 0.0245 was chosen to penalise for the multiple testing inherent in a 3-arm trial and to allow stopping for efficacy early in case of an unexpected strong efficacy outcome (see Additional Analyses 12.1 below).

3. Definitions

3.1. Symptomatic patients

For the purpose of establishing the secondary effectiveness endpoints, "symptomatic patients" are defined as patients with *P. vivax* parasitaemia and either a documented fever (axillary temperature ≥37.5°C) or a history of fever within the preceding 48 hours.

3.2. Study site

A study site is defined as study location(s) under the responsibility of one local Principal investigator (PI) (e.g. one site could consist of two study hospitals in the same area under the responsibility of the same PI).

3.3.Handling of missing data and adjudication of Endpoints

3.3.1. Handling of missing data for Adverse Events

For patients with missing data on adverse events (AE) the most conservative approach will be used:

Deviation	Action
Start date of AE missing	Assume during study drug intake
End date of AE missing	No imputation
Date of start of study treatment administration missing	All AEs after randomization considered to have happened during study drug intake
Missing assessment of relationship to study treatment	Assume event to be possibly related
Missing severity assessment of AE	Assume highest severity

3.3.2. Adjudication of effectiveness outcome assessment

For missed visits during the 6 months follow up, the following rules will apply:

Deviation	Action
More than 2 consecutive months without blood film examination.	-In the survival analysis lost to follow up (i.e. censored) on day of last visit before missing observation period.<br>-In the incidence rate analysis using asymptomatic recurrences as the event, the period of missing observation will be deducted from the total period of observation.<br>-In the incidence rate analysis using symptomatic recurrences as the event, the period of missing observation will not be deducted from the total period of observation.

3.3.3. Adjudication for haemoglobin outcome

For missing haemoglobin (Hb) measurements during follow up the following rules apply:

Outcome assessment	Deviation	Action
The incidence risk of severe (Hb <5g/dl) or moderate (≥5g/dl and <7g/dl) anaemia within 6 months	2 or more consecutive visits (only visits with scheduled Hb measurement) without Hb measurement.	Lost to follow up on day of last visit before missing observation period.

Incidence risk of an acute drop in Hb of >5g/dl within 3 days of starting treatment and incidence risk of an acute drop in Hb of >25% to <7g/dl within 3 days of starting treatment	Missing Hb measurement on day 3	Patient record set to missing for this outcome.

3.4. Analyses Populations

To provide a pragmatic comparison of the different drug treatments, the principle of intention-to-treat, will be the main strategy of analysis adopted for the primary and secondary endpoints. These analyses will be conducted on all patients assigned to the treatment groups as randomized, regardless of the study treatment received.

For the analysis of safety outcomes, all patients who received any study drug are included in the safety analysis in the treatment group they actually received. This means also patients having vomited the first dose and having had their treatment discontinued will be included in this analysis.

4. Analyses

4.1. General analyses strategy

The primary analysis will use the combined data from all sites and countries together, with adjustment for site effects. In addition, treatment effects will be estimated within each site, but formal assessment will not be performed for each site, since the study is not powered for this comparison.

4.2. Pooling across sites

As this is a randomized trial, with a common protocol and data collection and review tools across all sites, this pooled approach is expected to be valid. However, evidence for heterogeneity will be visually assessed and tested before deciding whether pooling of data across sites is justified (details provided in section 4.3 below).

4.3. Assessment of heterogeneity

The different sites have different burdens of malaria, which might lead to heterogeneity of the treatment effects of the incidence risk/rate of symptomatic *P.* vivax between study sites. For each outcome, we will estimate the coefficients (standard errors) using the statistical methods described in section 4.5) and assess heterogeneity between the estimates visually using forest plots. Random-

effects model will then be fitted to estimate I^2 values (percentage of total variation across studies due to heterogeneity) to statistically assess the degree of heterogeneity.

If substantial levels of heterogeneity are detected, site specific analyses will be performed only. If no substantial levels of heterogeneity are detected, then pooled analyses will be performed.

4.4.Demographic and baseline characteristics

Details of all patients screened, those who meet the study inclusion criteria, those who are eligible and randomized, those who are eligible but not randomized, those who withdraw from the study after randomization and those who are lost to follow-up will be summarized in a CONSORT flow diagram.

The number of patients discontinuing from the study will be tabulated by reason for study discontinuation. The number (%) of patients attending scheduled follow-up visits by study day will be reported.

The baseline value is defined as the last available value before randomization.

4.4.1. Demographic characteristics

- Gender: male / female
- Median Age (years), 25th, 75th percentiles, and range
- Median Weight (kg), 25th, 75th percentiles, and range

4.4.2. Disease characteristics at baseline

Specific disease history will include the parasite and gametocytes densities (/µL) at day 0, as well as axillary temperature (°C) (quantitative and if <37.5°C), Median Pulse rate (beats/min), 25th, 75th percentiles, haemoglobin and G6PD activity level, and range, and signs and symptoms (weakness, headache, anorexia, nausea, vomiting, pain, diarrhoea, convulsion, dehydration, icterus, sweat, chills, skin disorders) at day 0.

4.5.Effectiveness analyses

4.5.1. Incidence risk

The overall primary endpoint will be the incidence risk (95% CI) of vivax recurrence within 6 months of follow-up. The incidence risks will be calculated using the Kaplan-Meier (KM) method for each trial arm as well as a comparison of the relative hazards between trial arms (Hazard Ratio (95% CI)) estimated from a Cox regression analysis for the time to the first recurrent episode with adjustment for study site. Patients who are categorized as lost to follow up or have a non vivax parasitaemia will be censored at the day of the last visit.

4.5.2. Incidence rate

Incidence rates will be calculated by dividing the number of symptomatic P. vivax episodes by the number of person-years of observation in the study population, and compared between treatment arms using Poisson regression. The start date for person-years of observation will be the day of enrolment into the study and the stop date the last visit performed (either completed study at 6 months or any last visit before lost to follow up and/or censoring). The period between start and stop dates for each patient will be calculated in days and divided by 365 to determine the person-years of observation, which will then be totalled for all participants.

4.5.3. Subgroup analysis

A priori subgroup analyses will be performed for the incidence risk and rate of symptomatic *P. vivax* parasitaemia over 6 months in the following subgroups:

- By site and region: to compare the protective effectiveness in areas with different vivax relapse periodicity
- By schizontocidal drug: to assess whether effectiveness varies with schizontocidal partner drug (CQ, DHPA, Pyramax, ACTs)

4.6. Safety analyses

The primary safety concerns regarding primaquine and tafenoquine use pertain to the risk of haemolysis and gastroenterological adverse events.

4.6.1. Risk of Haemolysis

The safety of the treatments will be compared by calculating the number and proportion of patients that experience the following outcomes by day 3:

- Severe anaemia (Hb <5 g/dl);
- Moderate anaemia (Hb ≥5g/dl and Hb <7g/dl);
- Acute drop of Hb of >5 g/dl;
- A drop in Hb of >25% to <7 g/dl.

The number of patients requiring a blood transfusion at any time in the 6-month follow-up period will also be reported.

4.6.2. Risk of adverse events

The number and proportion of patients for whom PQ was discontinued or stopped after review on day 3 will be calculated for the PQ arms (Section 2.3.2 – safety endpoint 3).

The number and proportion of gastro-intestinal symptoms reported in all arms will be presented (Section 2.3.2 – safety endpoint 4). Gastro-intestinal symptoms are defined as patients with nausea, vomiting, and/or abdominal pain. Proportions for each symptom will be calculated separately.

The number and proportion of neuropsychiatric adverse events in the TQ arm will be calculated and compared to the PQ arms (Section 2.3.2 – safety endpoint 5).

The proportion of patients with one or more adverse events and serious adverse events within 42 days of their primary treatment will be presented. Events will be divided into related and unrelated events in regard to the treatment.

5. Interim analysis

5.1. Purpose of Interim Analysis

An unplanned interim analysis will be conducted at the availability of 6 months of follow-up data for 50% of the original sample size (i.e., 360 participants, which is 50% of 720 participants). The timing of this interim analysis is expected to be in April/May 2023, which is approximately 2 years after the first patient was recruited. At this point, the study will be continuing to enrol participants. An independent Statistician will perform the interim analysis and prepare a report for the DSMB. The interim analysis was added because of the protracted start of different study sites to potentially enable analysis of three sites only. The interim analysis will be conducted for the primary effectiveness outcome (2.3.1.1/4.5.1) and the first secondary effectiveness outcome (2.3.1.2(a)/ 4.5.1). Country specific analyses will not be conducted for the interim analysis.

The interim analysis will allow stopping for superiority (of the primary outcome) early using a conservative Haybittle-Peto boundary with early stopping to reject the null hypothesis. If the decision is made to stop the trial early, recruitment will cease in Indonesia and Ethiopia (noting that Cambodia has finished recruitment at the time of the interim analysis) but will continue in Pakistan. By stopping the trial early, the results from the three original sites (Cambodia, Indonesia and Ethiopia) will be made available without waiting for completion of recruitment and follow-up in Pakistan. Upon completion at the study site in Pakistan those results will then be combined with the three sites using a meta-analysis approach. No other aspects of the trial will be adapted or revised based on the results of the interim analysis, and the results of the interim analysis will not be used to adjust the sample size of this trial.

6. References

1. Abreha T, Hwang J, Thriemer K, Tadesse Y, Girma S, Melaku Z, et al. Comparison of artemether-lumefantrine and chloroquine with and without primaquine for the treatment of Plasmodium vivax infection in Ethiopia: A randomized controlled trial. PLoS Med. 2017;14(5):e1002299.

2. Llanos-Cuentas A, Lacerda MVG, Hien TT, Velez ID, Namaik-Larp C, Chu CS, et al. Tafenoquine versus Primaquine to Prevent Relapse of Plasmodium vivax Malaria. N Engl J Med. 2019;380(3):229-41.

3. Taylor WRJ, Thriemer K, von Seidlein L, Yuentrakul P, Assawariyathipat T, Assefa A, et al. Short-course primaquine for the radical cure of Plasmodium vivax malaria: a multicentre, randomised, placebo-controlled non-inferiority trial. Lancet. 2019;394(10202):929-38.

Text S7: Details on handing of missing data

Missing observations for the primary and secondary effectiveness outcomes were due to loss to follow up. The number and timing of these are indicated in Figure 1 and by the risk tables provided below the cumulative incidence plots presented in Figures 2 and 3. Safety data as adverse events to day 3 were missing in 22% of patients in the control arm, 24% of patients in the 7-day-high-dose primaquine arm and 25% of patients in the tafenoquine arm, also due to missed visits, and this is reflected in the denominators of these associated risks which are smaller than the total number of patients recruited. We do not assume that the patients lost to follow up were missing at random, the proportion missing was similar across the arms being compared. No imputation was performed for missing outcome data in either the effectiveness or safety analyses. Almost all of the safety events that were detected on day 3 were mild (grade 1-2) in nature and thus it is highly unlikely that significant adverse events have been missed.

Text S8: Details of sub-group analysis

The pre-specified sub-group analyses according to the SAP were by schizontocidal drug and study site. In two of the four countries where patients were recruited there was only one study site (Ethiopia and Indonesia). In Pakistan there were two sites, but only 22 patients were recruited across the three study arms at the site in Khidmat-e-alam (versus 218 patients at the other site in Thatta). There were three sites in Cambodia with only three patients recruited at the site in Chambak. The other two Cambodian sites at Kravanh and Siem Pang only had 100 and 117 patients enrolled in all three treatments arms.

Similarly, for the analysis by schizontocidal drug, Indonesia (DHA-piperaquine) and Cambodia (artesunate-pyronaridine) had different drugs, but Ethiopia and Pakistan both used chloroquine. However, Ethiopia had the largest country/site specific sample size and the greatest number of (first) recurrences (71) whereas Pakistan had relatively few (13).

In view of the relatively small numbers at the smaller sites, data were collated and analysed by country for the sub-group analyses.

Text S9: Details on interim analysis

The interim analysis was conducted by an independent statistician, based at the University of Melbourne but under a different supervisor than the trial statisticians, ensuring no direct reporting lines and thus independence. The analysis results were presented in a closed session to the Data Safety Monitoring Board (DSMB) by the independent statistician, without involvement of any of the study statisticians, who decided to continue recruitment; this decision was subsequently endorsed by the Trial Steering Committee. While the stopping rules were not binding, there was broad consensus within the DSMB. No formal template was used, and the interim results were not shared with the study team. No bias adjustment was done to account for the unplanned interim analysis.

Figure S1: Interim analysis results

Cumulative incidence of the first recurrence of Plasmodium vivax parasitaemia by 7-day-high-dose primaquine (total dose 7mg/kg; PQ7), tafenoquine (300mg; TQ) and 14-day-low-dose primaquine (total dose 3.5mg/kg, PQ14). The hazard ratio for 7-day-high dose primaquine compared to 14-day-low dose primaquine: 0.66 (95% CI 0.39-1.14)

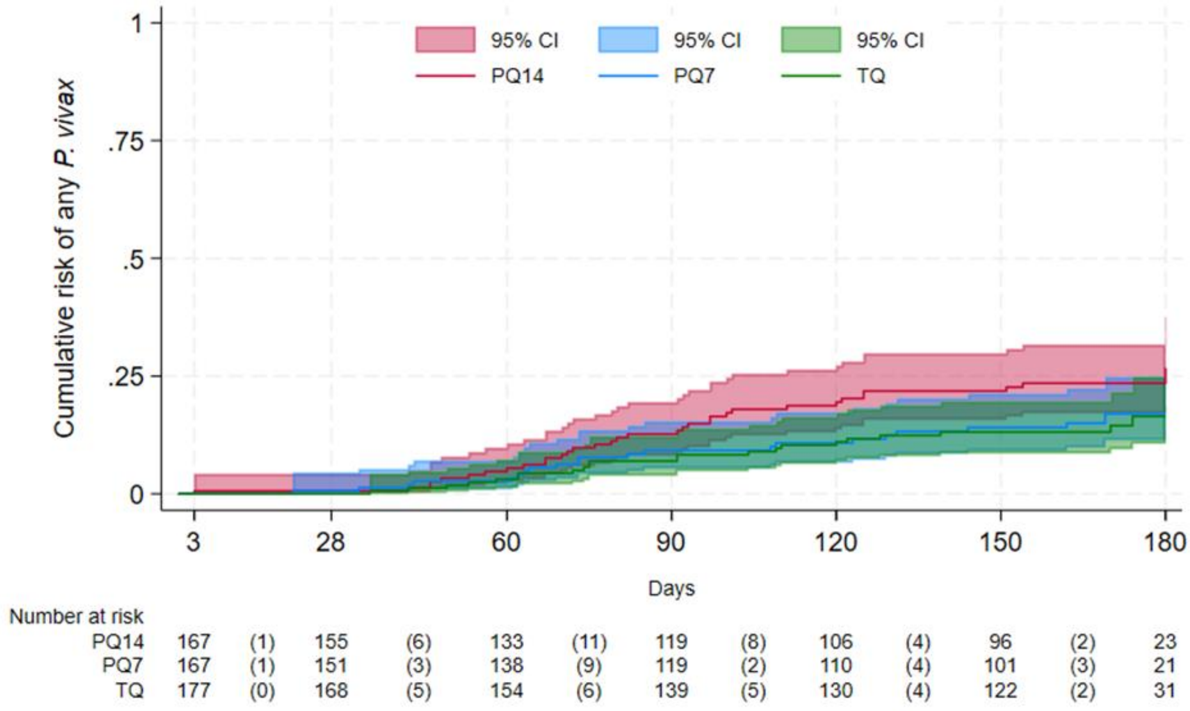

Table S10: Study sites and dates of enrolment

Study country		Start of enrolment	End of follow-up
Cambodia	Kravanh	April 2021	February 2023
	Siem Pang	May 2021	February 2023
	Chambak	July 2022	February 2023
Ethiopia	Arba Minch	January 2022	December 2023
Indonesia	Batubara, Sumatra	March 2022	December 2023
Pakistan	Karachi	May 2023	September 2024
	Thatta	April 2023	September 2024

Table S11: Baseline characteristics by study country

		7-day-high-dose primaquine*	Tafenoquine†	14-day-low-dose primaquine‡
**Cambodia**		**N= 73**	**N= 73**	**N= 74**
	Age, years	27.0 (22.0-34.0)	26.0 (21.0-35.0)	25.0 (22.0-33.0)
	Sex			
	Male	67 (91.8%)	64 (87.7%)	67 (90.5%)
	Female	6 (8.2%)	9 (12.3%)	7 (9.5%)
	Bodyweight, kg	60.0 (52.5-67.0)	57.0 (53.0-62.9)	59.4 (53.0-66.0)
	Height, metres	1.6 (1.6-1.7)	1.6 (1.6-1.7)	1.6 (1.6-1.7)
	Body mass index (BMI)	21.8 (20.3-24.1)	21.0 (19.8-22.9)	22.0 (20.0-23.5)
	P. vivax parasites/uL	5264 (2256.0-8160.0)	6784 (3968.0-15000.0)	5528 (2768.0-10560.0)
	Fever at presentation§	34 (46.6%)	46 (63.0%)	40 (54.1%)
	Body temperature, °C	37.4 (36.6-38.0)	37.8 (37.0-38.3)	37.5 (36.7-38.3)
	History of fever in previous 48 hours	73 (100.0%)	73 (100.0%)	74 (100.0%)
	Haemoglobin, g/dL	14.7 (2.0)	14.5 (1.8)	14.7 (1.5)
	G6PD activity, U/gHb	6.8 (6.1-7.7)	6.9 (6.2-8.5)	6.9 (6.0-8.1)
	G6PD activity <6 U/gHb	15 (20.5%)	12 (16.4%)	18 (24.3%)
**Ethiopia**		**N= 117**	**N= 117**	**N= 116**
	Age, years	22.0 (19.0-28.0)	24.0 (20.0-30.0)	23.0 (19.0-34.0)
	Sex			
	Male	65 (55.6%)	67 (57.3%)	65 (56.0%)
	Female	52 (44.4%)	50 (42.7%)	51 (44.0%)
	Bodyweight, kg	56.0 (51.0-61.0)	56.0 (52.0-60.0)	55.0 (51.0-61.0)
	Height, metres	1.7 (1.6-1.7)	1.6 (1.6-1.7)	1.6 (1.6-1.7)
	Body mass index (BMI)	20.2 (18.9-22.1)	20.3 (19.0-22.4)	20.3 (18.5-22.8)
	P. vivax parasites/uL	6144 (3040.0-17632.0)	6336 (1904.0-12608.0)	6368 (2456.0-13248.0)
	Fever at presentation§	30 (25.6%)	34 (29.1%)	34 (29.3%)
	Body temperature, °C	37.0 (36.7-37.5)	36.9 (36.7-37.8)	36.9 (36.8-37.8)
	History of fever in previous 48 hours	117 (100.0%)	117 (100.0%)	116 (100.0%)
	Haemoglobin, g/dL	13.8 (2.3)	14.1 (2.4)	14.2 (2.3)
	G6PD activity, U/gHb	7.0 (6.1-7.6)	6.8 (6.2-7.7)	6.9 (6.2-7.9)
	G6PD activity <6 U/gHb	22 (18.8%)	25 (21.4%)	18 (15.5%)
**Indonesia**		**N= 50**	**N= 50**	**N= 50**
	Age, years	28.5 (20.0-41.0)	30.5 (19.0-46.0)	23.0 (18.0-34.0)
	Sex			
	Male	35 (70.0%)	31 (62.0%)	34 (68.0%)
	Female	15 (30.0%)	19 (38.0%)	16 (32.0%)
	Bodyweight, kg	58.0 (50.3-69.0)	59.5 (48.4-69.3)	53.6 (49.2-60.5)
	Height, metres	1.6 (1.6-1.7)	1.6 (1.5-1.6)	1.6 (1.5-1.6)
	Body mass index (BMI)	21.9 (19.2-25.8)	23.7 (19.3-26.3)	20.6 (18.9-24.2)
	P. vivax parasites/uL	2352 (1456.0-4480.0)	1576 (736.0-4400.0)	2432 (1392.0-6600.0)

	Fever at presentation§	19 (38.0%)	18 (36.0%)	22 (44.0%)
	Body temperature, °C	36.8 (36.0-38.4)	37.0 (36.1-37.8)	37.1 (36.2-38.0)
	History of fever in previous 48 hours	50 (100.0%)	50 (100.0%)	50 (100.0%)
	Haemoglobin,g/dL	14.1 (2.0)	13.5 (2.4)	13.7 (2.1)
	G6PD activity, U/gHb	6.5 (6.1-7.2)	6.9 (6.2-7.6)	6.7 (6.1-7.9)
	G6PD activity <6 U/gHb	10 (20.0%)	6 (12.0%)	10 (20.0%)
**Pakistan**		**N= 80**	**N= 80**	**N= 80**
	Age, years	33.5 (24.0-44.5)	26.5 (21.5-41.0)	34.0 (24.0-44.0)
	Sex			
	Male	61 (76.2%)	53 (66.2%)	63 (78.8%)
	Female	19 (23.8%)	27 (33.8%)	17 (21.2%)
	Bodyweight, kg	56.0 (49.0-65.5)	55.0 (49.0-68.0)	55.2 (49.0-66.0)
	Height, metres	1.6 (1.6-1.7)	1.6 (1.5-1.7)	1.6 (1.6-1.7)
	Body mass index (BMI)	21.7 (18.6-24.4)	21.6 (19.1-24.6)	21.5 (18.2-25.3)
	P. vivax parasites/uL	1080 (480.0-2080.0)	1680 (560.0-2720.0)	1040 (424.0-2240.0)
	Fever at presentation§	7 (8.8%)	12 (15.0%)	9 (11.2%)
	Body temperature, °C	36.8 (36.3-37.1)	37.0 (36.5-37.2)	37.0 (36.5-37.2)
	History of fever in previous 48 hours	79 (98.8%)	80 (100.0%)	80 (100.0%)
	Haemoglobin, g/dL	13.4 (2.1)	12.8 (2.1)	13.4 (2.1)
	G6PD activity, U/gHb	8.6 (7.7-9.9)	8.7 (7.9-10.2)	8.6 (7.6-10.4)
	G6PD activity <6 U/gHb	2 (2.5%)	2 (2.5%)	2 (2.5%)

Data are n (%), median (interquartile range [IQR]) or mean (standard deviation [SD]) unless otherwise specified
*schizontocidal treatment of blood stage parasites plus 7mg/kg total dose of primaquine unsupervised over 7 days
†schizontocidal treatment of blood-stage parasites plus 300mg single dose of Tafenoquine
‡schizontocidal treatment of blood stage parasites plus 3.5mg/kg total dose of primaquine unsupervised over 14 days
§ axillary temperature of ≥37·5°C

Figure S2: Distribution of total primaquine doses administered in 7-day-high-dose and 14-day-low-dose primaquine arms, and total tafenoquine doses administered in the tafenoquine arm

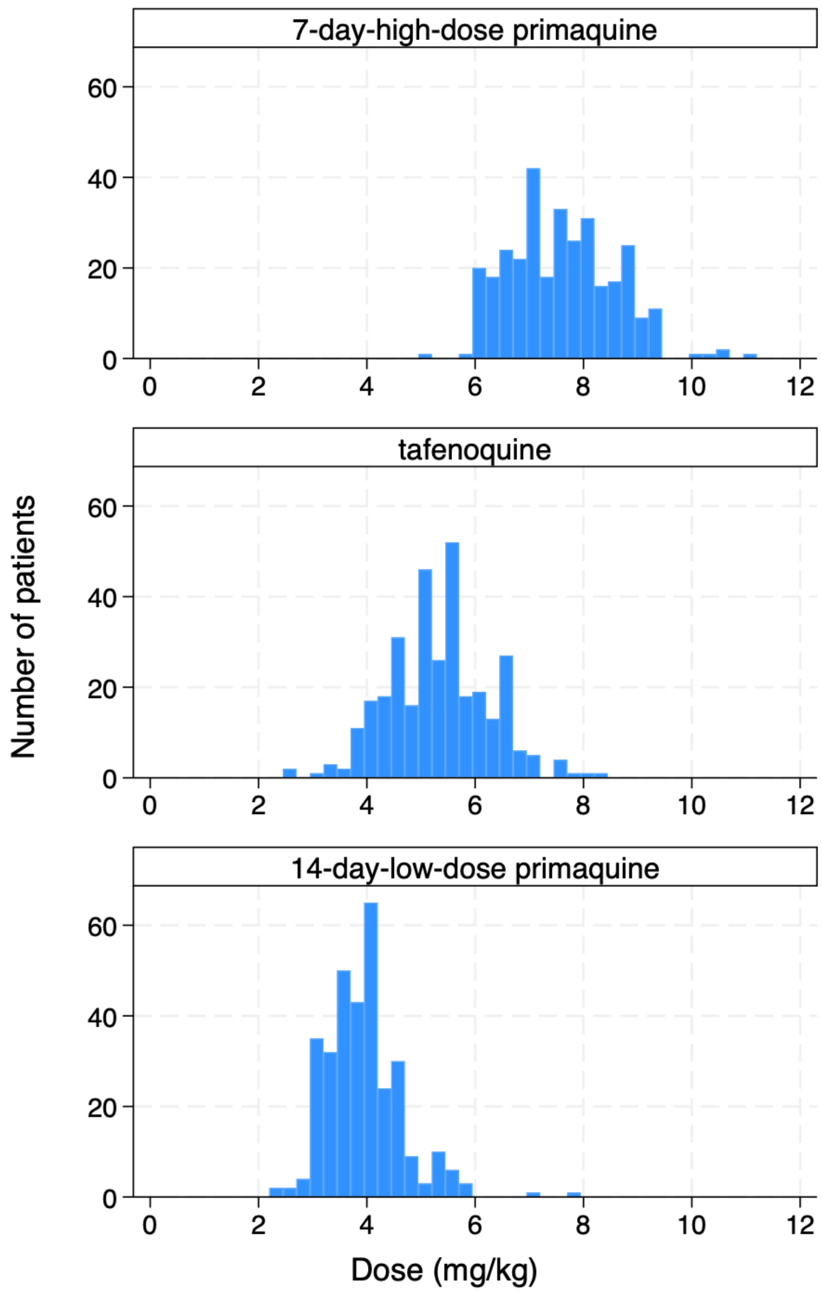

Table S12: Total primaquine doses administered in the 7-day-high-dose and 14-day-low-dose primaquine arms, and total tafenoquine dose administered in the tafenoquine arm by country

Site	mg/kg dose of primaquine in 7-day-high-dose primaquine arm	mg/kg dose of tafenoquine in tafenoquine arm	mg/kg dose of primaquine in 14-day-low-dose primaquine arm
Overall	7.6 (5.1-11.1)	5.3 (2.5-8.3)	4.0 (2.2-7.9)
Cambodia	7.5 (6.0-9.4)	5.3 (3.8-8.2)	3.8 (3.0-5.0)
Ethiopia	7.6 (6.0-9.3)	5.4 (3.3-7.1)	3.8 (2.2-5.8)
Indonesia	7.8 (5.8-11.1)	5.2 (2.7-7.7)	4.1 (3.1-5.7)
Pakistan	7.7 (5.1-10.5)	5.3 (2.5-8.3)	4.3 (3.0-7.9)

Data are presented as mean (range)

Table S13: Cumulative incidence of any *P. vivax* recurrence at 6 months by country

	Cumulative incidence (97.55% CI)			7-day-high dose primaquine vs 14-day-low-dose primaquine		Tafenoquine vs 14-day-low-dose primaquine		Tafenoquine vs 7-day-high dose primaquine	
	7-day-high dose primaquine*	Tafenoquine†	14-day-low-dose primaquine‡	HR (97.55% CI)	p value	HR (97.55% CI)	p value	HR (97.55% CI)	p value
**All sites**	13.0% (9.0-18.5)	12.6% (8.8-18.0)	18.5% (13.8-24.6)	0.66 (0.40-1.09)	0.063	0.64 (0.39-1.05)	0.041	0.96 (0.56-1.66)	0.875
**Cambodia**	17.1% (8.7-31.8)	15.6% (8.0-29.3)	22.1% (12.4-37.4)	0.77 (0.30-1.97)	0.527	0.72 (0.28-1.85)	0.432	0.94 (0.34-2.56)	0.886
**Ethiopia**	18.4% (11.5-28.5)	14.0% (8.0-23.6)	27.3% (18.9-38.5)	0.63 (0.33-1.20)	0.107	0.45 (0.22-0.93)	0.013	0.72 (0.34-1.56)	0.343
**Indonesia**	0.0% (0.0-10.7)	22.4% (9.8-46.2)	5.0% (0.5-38.5)			5.47 (0.49-60.53)	0.112		
**Pakistan**	5.6% (1.9-16.4)	4.1% (1.1-14.1)	8.1% (3.3-19.0)	0.70 (0.16-3.00)	0.584	0.51 (0.10-2.48)	0.334	0.72 (0.13-4.01)	0.666

CI, confidence interval; HR, hazard ratio
*schizontocidal treatment of blood stage parasites plus 7mg/kg total dose of primaquine unsupervised over 7 days
†schizontocidal treatment of blood-stage parasites plus 300mg single dose of tafenoquine
‡schizontocidal treatment of blood stage parasites plus 3.5mg/kg total dose of primaquine unsupervised over 14 days

Table S14: Total number of recurrences by study site

	7-day-high-dose primaquine	Tafenoquine	14-day-low-dose primaquine
**Total number of *P. vivax* recurrences**			
Cambodia – Kravanh (n=100)	5	7	8
Cambodia – Siem Pang (n=117)	6	3	7
Cambodia – Chambak (n=3)	0	3	0
Ethiopia (n=350)	27	21	41
Indonesia (n=150)	0	7	1
Pakistan – Thatta (n=218)	4	3	5
Pakistan – Khidmat-e-alam (n=22)	0	0	1
**Number of symptomatic *P. vivax* recurrences**			
Cambodia – Kravanh	5	5	6
Cambodia – Siem Pang	4	3	6
Cambodia – Chambak	0	1	0
Ethiopia	25	20	39
Indonesia	0	6	1
Pakistan – Thatta	4	3	5
Pakistan – Khidmat-e-alam	0	0	1

n = number of participants per site on day 0

Table S15: Number of patients with multiple recurrences

	7-day-high-dose primaquine	Tafenoquine	14-day-low-dose primaquine
**Number of patients with no vivax recurrences**			
Cambodia - Kravanh	29	26	26
Cambodia - Siem Pang	33	36	33
Cambodia - Chambak		1	1
Ethiopia	94	99	86
Indonesia	50	43	49
Pakistan - Thatta	68	70	68
Pakistan Khidmat-e-alam	8	7	6
**Number of patients with one vivax recurrence**			
Cambodia - Kravanh	5	5	8
Cambodia - Siem Pang	6	3	5
Cambodia - Chambak		0	0
Ethiopia	20	15	20
Indonesia	0	7	1
Pakistan - Thatta	4	3	5
Pakistan Khidmat-e-alam	0	0	1
**Number of patients with two vivax recurrences**			
Cambodia - Kravanh	0	1	0
Cambodia - Siem Pang	0	0	1
Cambodia - Chambak		0	0
Ethiopia	2	3	9
Indonesia	0	0	0
Pakistan - Thatta	0	0	0
Pakistan Khidmat-e-alam	0	0	0
**Number of patients with three vivax recurrences**			
Cambodia - Kravanh	0	0	0
Cambodia - Siem Pang	0	0	0
Cambodia - Chambak		1	0
Ethiopia	1	0	1
Indonesia	0	0	0
Pakistan - Thatta	0	0	0
Pakistan Khidmat-e-alam	0	0	0

Table S16: Summary of PCR confirmation of microscopy results of samples collected at enrolment

		7-day-high-dose primaquine*	Tafenoquine†	14-day-low-dose primaquine‡	TOTAL
**All sites**	*P. vivax* mono-infection	92% (296/320)	91% (292/320)	94% (301/320)	93% (889/960)
	P. falciparum mono-infection	0% (0/320)	1% (4/320)	1% (2/320)	1% (6/960)
	Mixed infection	4% (12/320)	5% (15/320)	2% (6/320)	3% (33/960)
	Negative	2% (8/320)	1% (4/320)	3% (9/320)	2% (21/960)_
	No PCR result	1% (4/320)	2% (5/320)	1% (2/320)	1% (11/960)
**Cambodia**	*P. vivax* mono-infection	95% (69/73)	99% (72/73)	96% (71/74)	96% (212/220)
	P. falciparum mono-infection	0% (0/73)	0% (0/73)	0% (0/74)	0% (0/220)
	Mixed infection	0% (0/73)	0% (0/73)	0% (0/74)	0% (0/220)
	Negative	1% (1/73)	0% (0/73)	3% (2/74)	1% (3/220)
	No PCR result	4% (3/73)	1% (1/73)	1% (1/74)	2% (5/220)
**Ethiopia**	*P. vivax* mono-infection	86% (101/117)	78% (91/117)	90% (104/116)	85% (296/350)
	P. falciparum mono-infection	0% (0/117)	3% (4/117)	2% (2/116)	2% (6/350)
	Mixed infection	9% (111/117)	13% (15/117)	4% (5/116)	9% (31/350)
	Negative	3% (4/117)	3% (3/117)	3% (4/116)	3% (11/350)
	No PCR result	1% (1/117)	3% (4/117)	1% (1/116)	2% (6/350)
**Indonesia**	*P. vivax* mono-infection	100% (50/50)	100% (50/50)	100% (50/50)	100% (150/150)
	P. falciparum mono-infection	0% (0/50)	0% (0/50)	0% (0/50)	0% (0/150)
	Mixed infection	0% (0/50)	0% (0/50)	0% (0/50)	0% (0/150)
	Negative	0% (0/50)	0% (0/50)	0% (0/50)	0% (0/150)
	No PCR result	0% (0/50)	0% (0/50)	0% (0/50)	0% (0/150)
**Pakistan**	*P. vivax* mono-infection	95% (76/80)	99% (79/80)	95% (76/80)	96% (231/240
	P. falciparum mono-infection	0% (0/80)	0% (0/80)	0% (0/80)	0% (0/240)
	Mixed infection	1% (1/80)	0% (0/80)	1% (1/80)	1% (2/240)
	Negative	4% (3/80)	1% (1/80)	4% (3/80)	3% (7/240)
	No PCR result	0% (0/80)	0% (0/80)	0% (0/80)	0% (0/240)

*schizontocidal treatment of blood stage parasites plus 7mg/kg total dose of primaquine unsupervised over 7 days

†schizontocidal treatment of blood-stage parasites plus 300mg single dose of tafenoquine

‡schizontocidal treatment of blood stage parasites plus 3.5mg/kg total dose of primaquine unsupervised over 14 days

Table S17: Summary of PCR confirmation of microscopy results of samples collected at first *P. vivax* recurrence

		7-day-high-dose primaquine*	Tafenoquine†	14-day-low-dose primaquine‡	TOTAL
**All sites**	*P. vivax* infection	24/34 (71%)	26/35 (74%)	35/49 (71%)	85/118 (72%)
	P. falciparum mono-infection	3/34 (9%)	0/35 (0%)	1/49 (2%)	4/118 (3%)
	Negative	1/34 (3%)	1/35 (3%)	1/49 (2%)	3/118 (3%)
	No PCR result	2/34 (6%)	4/35 (11%)	4/49 (8%)	10/118 (8%)
	Excluded as *P.vivax* infection at baseline not confirmed	4/34 (12%)	4/35 (11%)	8/49 (16%)	16/118 (14%)
**Cambodia**	*P. vivax* infection	10/10 (100%)	9/10 (90%)	12/13 (91%)	31 (94%)
	P. falciparum mono-infection	10/10 (100%)	9/10 (90%)	12/13 (92%)	31/33 (94%)
	Negative	0/10 (0%)	0/10 (0%)	0/13 (0%)	0/33 (0%)
	No PCR result	0/10 (0%)	0/10 (0%)	0/13 (0%)	0/33 (0%)
	Excluded as *P.vivax* infection at baseline not confirmed	0/10 (0%)	1/10 (10%)	0/13 (0%)	1/33 (3%)
**Ethiopia**	*P. vivax* infection	13/20 (65%)	10/15 (67%)	20/29 (69%)	43/64 (67%)
	P. falciparum mono-infection	3/20 (15%)	0/15 (0%)	1/29 (3%)	4/64 (6%)
	Negative	0/20 (0%)	1/15 (7%)	1/29 (3%)	2/64 (3%)
	No PCR result	0/20 (0%)	0/15 (0%)	1/29 (3%)	1/64 (2%)
	Excluded as *P.vivax* infection at baseline not confirmed	4/20 (20%)	4/15 (27%)	6/29 (21%)	14/64 (22%)
**Indonesia**	*P. vivax* infection	0/0 (0%)	6/7 (86%)	1/1 (100%)	7/8 (88%)
	P. falciparum mono-infection	0/0 (0%)	0/7 (0%)	0/1 (0%)	0/8 (0%)
	Negative	0/0 (0%)	0/7 (0%)	0/1 (0%)	0/8 (0%)
	No PCR result	0/0 (0%)	1/7 (14%)	0/1 (0%)	1/8 (12%)
	Excluded as *P.vivax* infection at baseline not confirmed	0/0 (0%)	6/7 (86%)	1/1 (100%)	7/8 (88%)
**Pakistan**	*P. vivax* infection	1/4 (25%)	1/3 (33%)	2/6 (33%)	4/13 (31%)
	P. falciparum mono-infection	0/4 (0%)	0/3 (0%)	0/6 (0%)	0/13 (0%)
	Negative	1/4 (25%)	0/3 (0%)	0/6 (0%)	1/13 (8%)
	No PCR result	2/4 (50%)	2/3 (67%)	3/6 (50%)	7/13 (54%)
	Excluded as *P.vivax* infection at baseline not confirmed	0/4 (0%)	0/3 (0%)	1/6 (17%)	1/13 (8%)

*schizontocidal treatment of blood stage parasites plus 7mg/kg total dose of primaquine unsupervised over 7 days
†schizontocidal treatment of blood-stage parasites plus 300mg single dose of tafenoquine
‡schizontocidal treatment of blood stage parasites plus 3.5mg/kg total dose of primaquine unsupervised over 14 days

Table S18: Sensitivity analysis excluding patients where *P. vivax* parasitaemia could not be confirmed by PCR (overall and by country)

	**Cumulative incidence of any *P. vivax* parasitemia at 6 months, (97.55% CI)**			**7-day-high-dose primaquine vs 14-day-low-dose primaquine**		**Tafenoquine vs 14-day-low-dose primaquine**		**Tafenoquine vs 7-day-high-dose primaquine**	
	**7-day-high-dose primaquine***	**Tafenoquine†**	**14-day-low-dose primaquine‡**	**HR (97.55% CI)**	**p value**	**HR (97.55% CI)**	**p value**	**HR (97.55% CI)**	**p value**
**All sites**	10.5% (6.8-16.0)	10.6% (7.0-16.0)	14.1% (9.9-20.0)	0.72 (0.40-1.30)	0.212	0.74 (0.25-1.32)	0.246	1.03 (0.55-1.93)	0.915
**Cambodia**	18.3 (9.4,33.9)	14.2 (7.0,27.8)	21.5 (11.8,37.2)	0.85 (0.33,2.24)	0.711	0.68 (0.25,1.84)	0.388	0.8 (0.28,2.25)	0.628
**Ethiopia**	14.9 (8.5,25.6)	13.0 (6.8,24.0)	20.6 (13.0,31.8)	0.70 (0.32,1.52)	0.299	0.59 (0.25,1.36)	0.153	0.84 (0.34,2.08)	0.666
**Indonesia**	0.0 (0.0,10.7)	19.7 (8.0,44.0)	5.0 (0.5,38.5)			4.63 (0.41,52.57)	0.156		
**Pakistan**	1.5 (0.2,13.5)	1.4 (0.1,12.3)	2.8 (0.6,13.2)	0.51 (0.03,8.45)	0.613	0.49 (0.03,7.72)	0.562	0.91 (0.04,21.98)	0.949

CI, confidence interval; HR, hazard ratio

*schizontocidal treatment of blood stage parasites plus 7mg/kg total dose of primaquine unsupervised over 7 days

†schizontocidal treatment of blood-stage parasites plus 300mg single dose of tafenoquine

‡schizontocidal treatment of blood stage parasites plus 3.5mg/kg total dose of primaquine unsupervised over 14 days

Figure S3: Identity-by-descent distribution amongst baseline infections

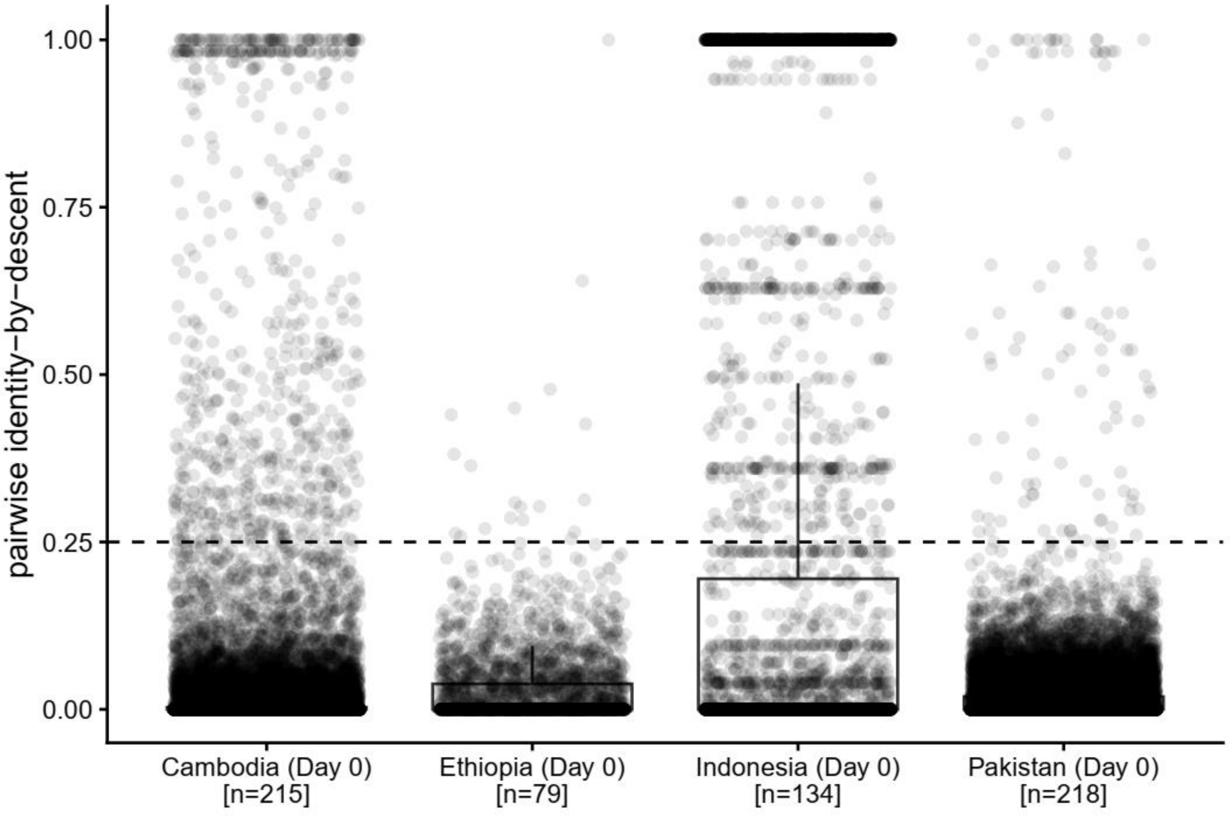

Pairwise identity-by-descent distribution (IBD) (between relatedness) is presented on independent (day 0) infections pooled across the treatment arms in each of Cambodia (n=212), Ethiopia (n=79), Indonesia (n=134) and Pakistan (n=218).

Table S19: Homologous recurrences overall and per country

		7-day-high-dose primaquine*	Tafenoquine†	14-day-low-dose primaquine‡
**All sites**	Total number of first recurrences (microscopy)	34	35	49
	Number of PCR-confirmed paired samples	24	27	34
	Number of paired samples with genetic data	24	23	31
	Number of homologous recurrences (%)	9 (38%)	11 (48%)	12 (39%)
**Cambodia**	Total number of first recurrences (microscopy)	10	10	13
	Number of PCR-confirmed paired samples	10	9	11
	Number of paired samples with genetic data	10	9	11
	Number of homologous recurrences (%)	5 (50%)	8 (89%)	7 (64%)
**Ethiopia**	Total number of first recurrences (microscopy)	20	15	29
	Number of PCR-confirmed paired samples	14	11	20
	Number of paired samples with genetic data	13	10	18
	Number of homologous recurrences (%)	4 (31%)	2 (20%)	6 (33%)
**Indonesia**	Total number of first recurrences (microscopy)	0	7	1
	Number of PCR-confirmed paired samples	0	6	1
	Number of paired samples with genetic data	0	3	1
	Number of homologous recurrences (%)	0 (0%)	0 (0%)	0 (0%)
**Pakistan**	Total number of first recurrences (microscopy)	4	3	6
	Number of PCR-confirmed paired samples	1	1	2
	Number of paired samples with genetic data	1	1	1
	Number of homologous recurrences (%)	0 (0%)	1 (100%)	0 (0%)

The microhaplotype-derived Identity-by-descent distribution (IBD) was used to classify recurrences as heterologous (suspected relapse or reinfection) and homologous (suspected relapse or recrudescence). Recurrences were classified as homologous to the enrolment case if IBD ≥0.25 and heterologous if IBD <0.25

*schizontocidal treatment of blood stage parasites plus 7mg/kg total dose of primaquine unsupervised over 7 days
† schizontocidal treatment of blood-stage parasites plus 300mg single dose of tafenoquine
‡ schizontocidal treatment of blood stage parasites plus 3.5mg/kg total dose of primaquine unsupervised over 14 days

Figure S4: Tafenoquine dose (mg/kg) in patients with and without recurrences in Tafenoquine arm

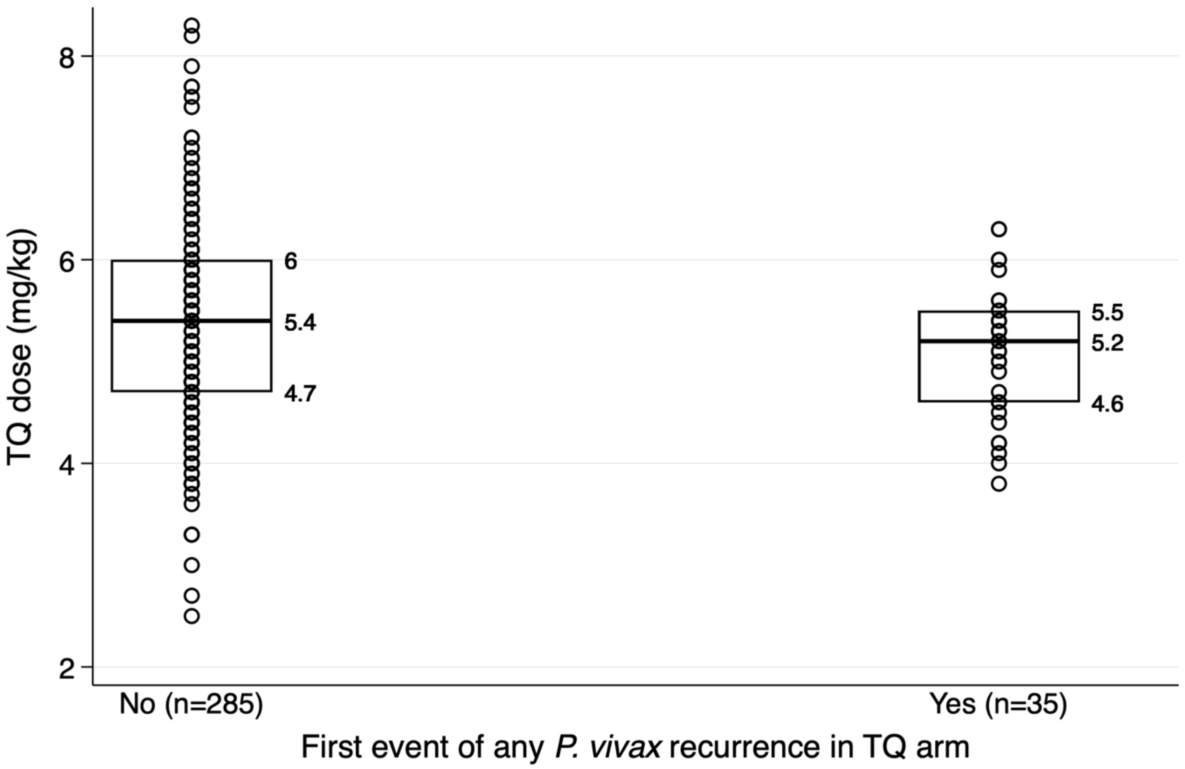

Figure S5: Tafenoquine dose (mg/kg) in patients with homologous and heterologous recurrences and no recurrences in Tafenoquine arm

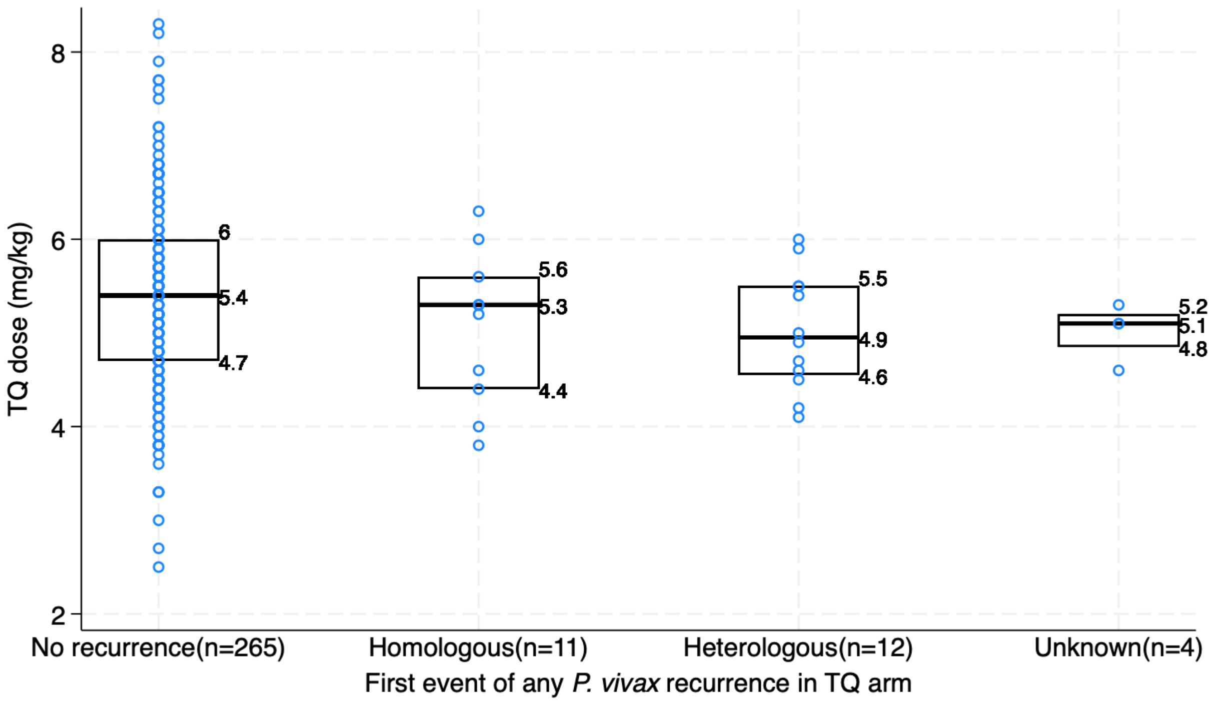

Figure S6: Tafenoquine dose (mg/kg) of patients with and without recurrences in each country in Tafenoquine arm

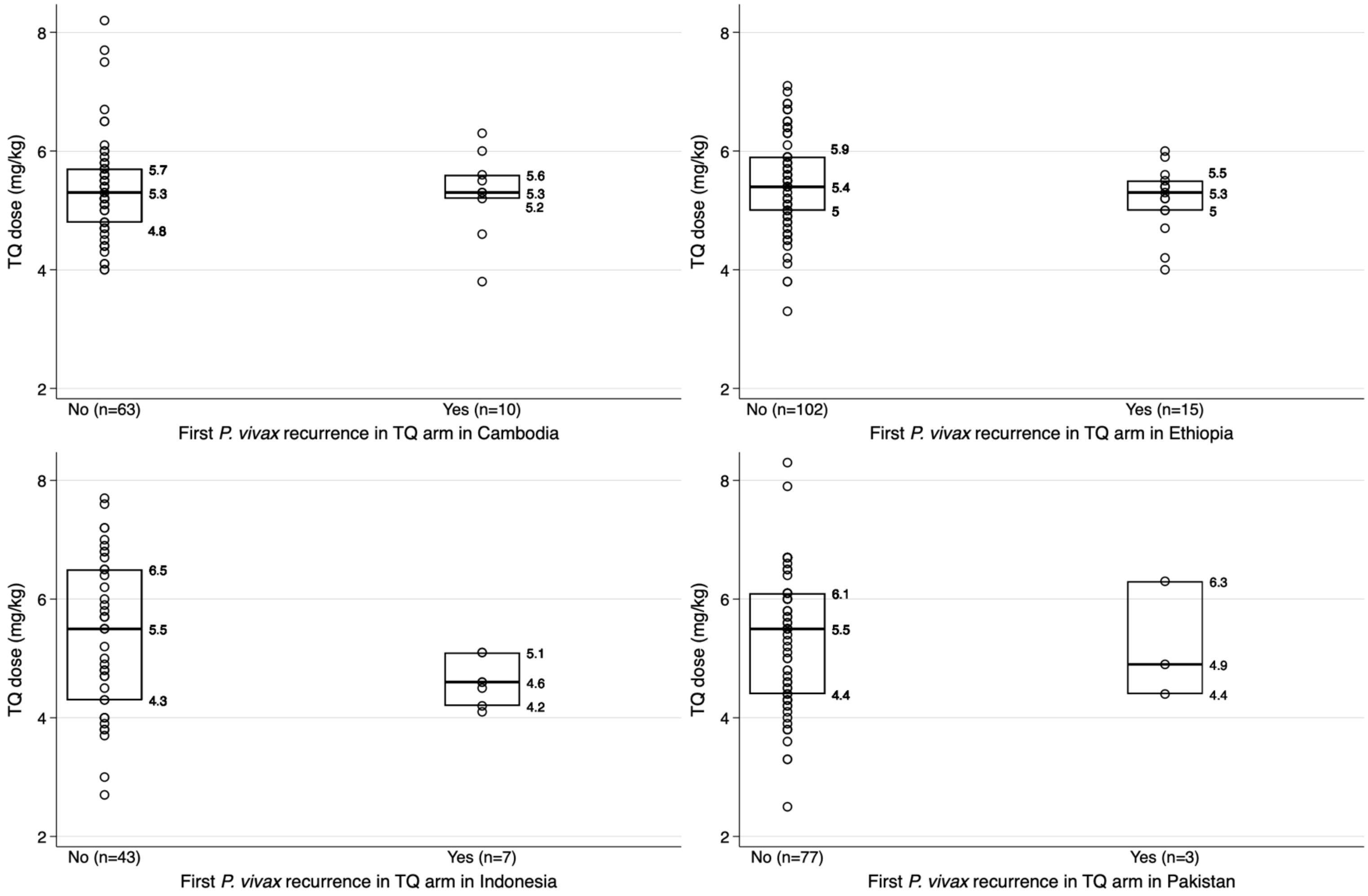

Table S20: Details of the three patients with an acute drop of haemoglobin (Hb) of >5g/dL by day 3

N#	Study arm	Country	Age (years)	Sex	G6PD activity at enrolment (U/gHb)	Hb at enrolment (g/dL)	Hb at day 3 (g/dL)	Hb at day 4 (g/dL)	Hb at day 28 (g/dL)
1	Tafenoquine	Indonesia	17	male	5.1	15.8	10.6	13.4	12.0
2	Tafenoquine	Ethiopia	28	male	7.9	18.7	13.7	Not available	16.5
3	14-day-low dose primaquine	Indonesia	18	male	5.2	19.2	11.7	13.7	15.8

Figure S7: Change in haemoglobin between Day 0 and Day 3 in patients with G6PD <6 U/gHb in the 7-day-high-dose primaquine arm, tafenoquine arm and 14-day-low-dose primaquine arm

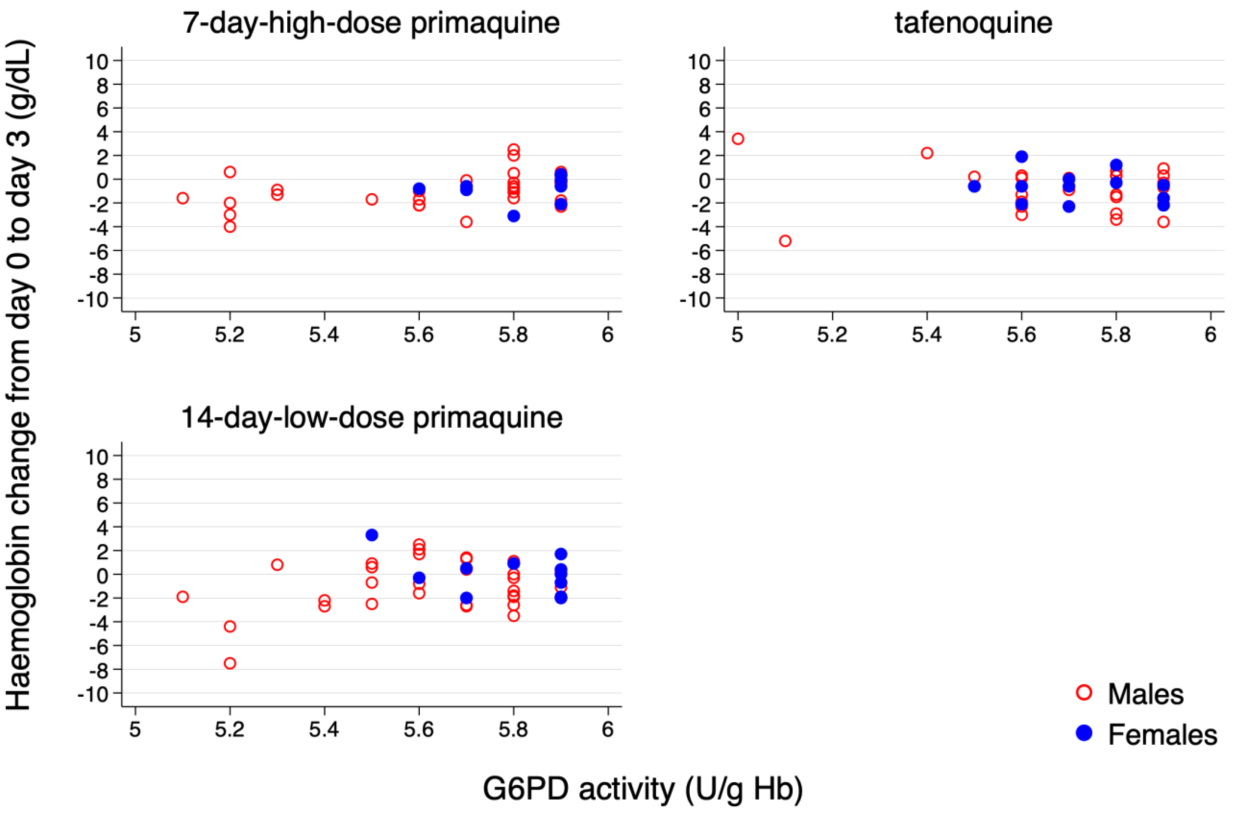

Table S21: Safety and tolerability outcomes, Cambodia

Adverse Events	7-day-high-dose primaquine* N=73	Tafenoquine† N=7	14-day-low-dose primaquine‡ N=74
**All adverse events until day 42**	**15**	**24**	**10**
Study drug related§	2/15 (13%)	0/24 (0%)	0/10 (0%)
Study drug unrelated	13/15 (87%)	24/24 (100%)	10/10 (100%)
Grade 1¶	10/15 (67%)	20/24 (83%)	6/10 (60%)
Grade 2‖	4/15 (27%)	4/24 (17%)	4/10 (40%)
Grade 3**	1/15 (7%)	0/24 (0%)	0/10 (0%)
Adverse events occurring from day 0-3	4/15 (27%)	1/24 (4%)	1/10 (10%)
Adverse events occurring from day 4-42	11/15 (73%)	23/24 (96%)	9/10 (90%)
Adverse events until day 42 grouped			
Hb drop/Anaemia	0/15 (0%)	0/24 (0%)	0/10 (0%)
Gastrointestinal	2/15 (13%)	6/24 (25%)	2/10 (20%)
Respiratory	2/15 (13%)	7/24 (29%)	1/10 (10%)
Musculoskeletal	3/15 (20%)	2/24 (8%)	0/10 (0%)
Febrile illness	2/15 (13%)	4/24 (17%)	2/10 (20%)
Neuro-psychiatric	0/15 (0%)	0/24 (0%)	0/10 (0%)
General unwell	3/15 (20%)	4/24 (17%)	1/10 (10%)
Others	3/15 (20%)	1/24 (4%)	4/10 (40%)
**Symptoms occurring since enrolment and reported on day 3**			
Any gastrointestinal symptoms††	46/57 (81%)	53/63 (84%)	50/63 (79%)
Vomiting	6/57 (11%)	10/63 (16%)	9/63 (14%)
Nausea	21/57 (37%)	29/63 (46%)	28/63 (44%)
Diarrhoea	5/57 (9%)	13/63 (21%)	6/63 (10%)
Loss of appetite	37/57 (65%)	43/63 (68%)	38/63 (60%)
Abdominal pain	26/57 (46%)	25/63 (40%)	23/63 (37%)
Headache	53/57 (93%)	60/63 (95%)	60/63 (95%)
Muscle pain	41/57 (72%)	33/63 (52%)	40/63 (63%)
Joint pain	38/57 (67%)	38/63 (60%)	43/63 (68%)
Fever	55/57 (96%)	62/63 (98%)	63/63 (100%)
Dark urine	0/57 (0%)	0/63 (0%)	0/63 (0%)
Dizziness	34/57 (60%)	41/63 (65%)	43/63 (68%)
Shortness of breath	1/57 (2%)	5/63 (8%)	2/63 (3%)
Irritability	1/57 (2%)	2/63 (3%)	3/63 (5%)
Jaundice	2/57 (4%)	0/63 (0%)	0/63 (0%)
Fatigue	40/57 (70%)	51/63 (81%)	49/63 (78%)
Malaise	18/57 (32%)	20/63 (32%)	22/63 (35%)
Chills	48/57 (84%)	62/63 (98%)	60/63 (95%)

*schizontocidal treatment of blood stage parasites + 7mg/kg total dose of primaquine unsupervised over 7 days
†schizontocidal treatment of blood-stage parasites + 300mg single dose of tafenoquine
‡ schizontocidal treatment of blood stage parasites + 3.5mg/kg total dose of primaquine unsupervised over 14 days
§ study drug related events are possibly, probably, or definitely related to tafenoquine or primaquine
¶ mild symptoms causing no or minimal interference with usual activities
|| moderate symptoms causing greater than minimal interference with usual activities
** severe symptoms causing inability to perform usual activities
†† includes at least any of the following: vomiting, diarrhoea, loss of appetite, nausea and abdominal pain

Table S22: Safety and tolerability outcomes, Ethiopia

Adverse Events	7-day-high-dose primaquine* N=117	Tafenoquine† N=117	14-day-low-dose primaquine‡ N=116
**All adverse events until day 42**	**8**	**3**	**2**
Study drug related§	8/8 (100%)	3/3 (100%)	1/2 (50%)
Study drug unrelated	0/8 (0%)	0/3 (0%)	1/2 (50%)
Grade 1¶	8/8 (100%)	3/3 (100%)	2/2 (100%)
Grade 2‖	0/8 (0%)	0/3 (0%)	0/2 (0%)
Grade 3**	0/8 (0%)	0/3 (0%)	0/2 (0%)
Adverse events occurring from day 0-3	8/8 (100%)	3/3 (100%)	1/2 (50%)
Adverse events occurring from day 4-42	0/8 (0%)	0/3 (0%)	1/2 (50%)
Adverse events until day 42 grouped			
Hb drop/Anaemia	0/8 (0%)	0/3 (0%)	0/2 (0%)
Gastrointestinal	6/8 (75%)	3/3 (100%)	1/2 (50%)
Respiratory	0/8 (0%)	0/3 (0%)	0/2 (0%)
Musculoskeletal	0/8 (0%)	0/3 (0%)	0/2 (0%)
Febrile illness	0/8 (0%)	0/3 (0%)	1/2 (50%)
Neuro-psychiatric	0/8 (0%)	0/3 (0%)	0/2 (0%)
General unwell	1/8 (12%)	0/3 (0%)	0/2 (0%)
Others	1/8 (12%)	0/3 (0%)	0/2 (0%)
**Symptoms occurring since enrolment and reported on day 3**	**106**	**105**	**108**
Any gastrointestinal symptoms††	82/106 (77%)	88/105 (84%)	84/108 (78%)
Vomiting	31/106 (29%)	34/105 (32%)	25/108 (23%)
Nausea	42/106 (40%)	44/105 (42%)	37/108 (34%)
Diarrhoea	1/106 (1%)	2/105 (2%)	1/108 (1%)
Loss of appetite	80/106 (75%)	85/105 (81%)	81/108 (75%)
Abdominal pain	2/106 (2%)	0/105 (0%)	0/108 (0%)
Headache	105/106 (99%)	105/105 (100%)	108/108 (100%)
Muscle pain	106/106 (100%)	105/105 (100%)	108/108 (100%)
Joint pain	106/106 (100%)	105/105 (100%)	108/108 (100%)
Fever	106/106 (100%)	105/105 (100%)	108/108 (100%)
Dark urine	0/106 (0%)	1/105 (1%)	0/108 (0%)
Dizziness	70/106 (66%)	66/105 (63%)	69/108 (64%)
Shortness of breath	0/106 (0%)	0/105 (0%)	0/108 (0%)
Irritability	0/106 (0%)	1/105 (1%)	0/108 (0%)
Jaundice	0/106 (0%)	0/105 (0%)	0/108 (0%)
Fatigue	106/106 (100%)	105/105 (100%)	108/108 (100%)
Malaise	104/106 (98%)	105/105 (100%)	108/108 (100%)
Chills	104/106 (98%)	105/105 (100%)	108/108 (100%)

*schizontocidal treatment of blood stage parasites + 7mg/kg total dose of primaquine unsupervised over 7 days
†schizontocidal treatment of blood-stage parasites + 300mg single dose of tafenoquine
‡ schizontocidal treatment of blood stage parasites + 3.5mg/kg total dose of primaquine unsupervised over 14 days
§ study drug related events are possibly, probably, or definitely related to tafenoquine or primaquine
¶ mild symptoms causing no or minimal interference with usual activities
|| moderate symptoms causing greater than minimal interference with usual activities
** severe symptoms causing inability to perform usual activities
†† includes at least any of the following: vomiting, diarrhoea, loss of appetite, nausea and abdominal pain

Table S23: Safety and tolerability outcomes, Indonesia

Adverse Events	7-day-high-dose Primaquine* N=50	Tafenoquine† N=50	14-day-low-dose Primaquine‡ N=50
**All adverse events until day 42**	**23**	**31**	**21**
Study drug related§	13/23 (57%)	9/31 (29%)	9/21 (43%)
Study drug unrelated	10/23 (43%)	22/31 (71%)	12/21 (57%)
Grade 1¶	23/23 (100%)	30/31 (97%)	20/21 (95%)
Grade 2‖	0/23 (0%)	1/31 (3%)	1/21 (5%)
Grade 3**	0/23 (0%)	0/31 (0%)	0/21 (0%)
Adverse events occurring from day 0-3	8/23 (35%)	3/31 (10%)	7/21 (33%)
Adverse events occurring from day 4-42	15/23 (65%)	28/31 (90%)	14/21 (67%)
Adverse events until day 42 grouped			
Hb drop/Anaemia	3/23 (13%)	3/31 (10%)	5/21 (24%)
Gastrointestinal	7/23 (30%)	4/31 (13%)	3/21 (14%)
Respiratory	4/23 (17%)	14/31 (45%)	8/21 (38%)
Musculoskeletal	1/23 (4%)	0/31 (0%)	1/21 (5%)
Febrile illness	2/23 (9%)	6/31 (19%)	0/21 (0%)
Neuro-psychiatric	0/23 (0%)	0/31 (0%)	0/21 (0%)
General unwell	5/23 (22%)	3/31 (10%)	3/21 (14%)
Others	1/23 (4%)	1/31 (3%)	1/21 (5%)
**Symptoms occurring since enrolment and reported on day 3**	**39**	**35**	**37**
Any gastrointestinal symptoms††	31/39 (79%)	28/35 (80%)	29/37 (78%)
Vomiting	15/39 (38%)	10/35 (29%)	18/37 (49%)
Nausea	28/39 (72%)	18/35 (51%)	22/37 (59%)
Diarrhoea	2/39 (5%)	1/35 (3%)	2/37 (5%)
Loss of appetite	15/39 (38%)	16/35 (46%)	14/37 (38%)
Abdominal pain	11/39 (28%)	9/35 (26%)	14/37 (38%)
Headache	31/39 (79%)	28/35 (80%)	34/37 (92%)
Muscle pain	28/39 (72%)	18/35 (51%)	16/37 (43%)
Joint pain	25/39 (64%)	20/35 (57%)	15/37 (41%)
Fever	37/39 (95%)	33/35 (94%)	35/37 (95%)
Dark urine	1/39 (3%)	2/35 (6%)	0/37 (0%)
Dizziness	19/39 (49%)	14/35 (40%)	14/37 (38%)
Shortness of breath	8/39 (21%)	3/35 (9%)	2/37 (5%)
Irritability	0/39 (0%)	0/35 (0%)	0/37 (0%)
Jaundice	0/39 (0%)	0/35 (0%)	0/37 (0%)
Fatigue	21/39 (54%)	19/35 (54%)	17/37 (46%)
Malaise	18/39 (46%)	11/35 (31%)	9/37 (24%)
Chills	36/39 (92%)	32/35 (91%)	34/37 (92%)

*schizontocidal treatment of blood stage parasites + 7mg/kg total dose of primaquine unsupervised over 7 days
†schizontocidal treatment of blood-stage parasites + 300mg single dose of tafenoquine
‡ schizontocidal treatment of blood stage parasites + 3.5mg/kg total dose of primaquine unsupervised over 14 days
§ study drug related events are possibly, probably, or definitely related to tafenoquine or primaquine
¶ mild symptoms causing no or minimal interference with usual activities
|| moderate symptoms causing greater than minimal interference with usual activities
** severe symptoms causing inability to perform usual activities
†† includes at least any of the following: vomiting, diarrhoea, loss of appetite, nausea and abdominal pain

Table S24: Safety and tolerability outcomes, Pakistan

Adverse Events	7-day-high-dose Primaquine* N=80	Tafenoquine† N=80	14-day-low-dose Primaquine‡ N=80
**All adverse events until day 42**	**10**	**14**	**5**
Study drug related§	1/10 (10%)	4/14 (29%)	3/5 (60%)
Study drug unrelated	9/10 (90%)	10/14 (71%)	2/5 (40%)
Grade 1¶	8/10 (80%)	11/14 (79%)	3/5 (60%)
Grade 2‖	2/10 (20%)	2/14 (14%)	2/5 (40%)
Grade 3**	0/10 (0%)	1/14 (7%)	0/5 (0%)
Adverse events occurring from day 0-3	6/10 (60%)	13/14 (93%)	3/5 (60%)
Adverse events occurring from day 4-42	4/10 (40%)	1/14 (7%)	2/5 (40%)
Adverse events until day 42 grouped			
Hb drop/Anaemia	0/10 (0%)	0/10 (0%)	0/5 (0%)
Gastrointestinal	7/10 (70%)	9/14 (64%)	5/5 (100%)
Respiratory	0/10 (0%)	1/14 (7%)	0/5 (0%)
Musculoskeletal	0/10 (0%)	2/14 (14%)	0/5 (0%)
Febrile illness	1/10 (10%)	0/14 (0%)	0/5 (0%)
Neuro-psychiatric	0/10 (0%)	0/10 (0%)	0/5 (0%)
General unwell	2/10 (20%)	2/14 (14%)	0/5 (0%)
Others	0/10 (0%)	0/14 (0%)	0/5 (0%)
**Symptoms occurring since enrolment and reported on day 3**	**40**	**36**	**41**
Any gastrointestinal symptoms††	24/40 (60%)	25/36 (69%)	24/41 (59%)
Vomiting	8/40 (20%)	11/36 (31%)	11/41 (27%)
Nausea	7/40 (18%)	8/36 (22%)	7/41 (17%)
Diarrhoea	1/40 (2%)	4/36 (11%)	0/41 (0%)
Loss of appetite	15/40 (38%)	8/36 (22%)	12/41 (29%)
Abdominal pain	7/40 (18%)	4/36 (11%)	8/41 (20%)
Headache	32/40 (80%)	19/36 (53%)	23/41 (56%)
Muscle pain	19/40 (48%)	14/36 (39%)	15/41 (37%)
Joint pain	12/40 (30%)	13/36 (36%)	12/41 (29%)
Fever	40/40 (100%)	36/36 (100%)	41/41 (100%)
Dark urine	1/40 (2%)	1/36 (3%)	0/41 (0%)
Dizziness	8/40 (20%)	12/36 (33%)	6/41 (15%)
Shortness of breath	2/40 (5%)	1/36 (3%)	2/41 (5%)
Irritability	0/40 (0%)	0/36 (0%)	0/41 (0%)
Jaundice	4/40 (10%)	1/36 (3%)	1/41 (2%)
Fatigue	31/40 (78%)	25/36 (69%)	29/41 (71%)
Malaise	5/40 (12%)	2/36 (6%)	3/41 (7%)
Chills	34/40 (85%)	29/36 (81%)	35/41 (85%)

*schizontocidal treatment of blood stage parasites + 7mg/kg total dose of primaquine unsupervised over 7 days
†schizontocidal treatment of blood-stage parasites + 300mg single dose of tafenoquine
‡ schizontocidal treatment of blood stage parasites + 3.5mg/kg total dose of primaquine unsupervised over 14 days
§ study drug related events are possibly, probably, or definitely related to tafenoquine or primaquine
¶ mild symptoms causing no or minimal interference with usual activities
|| moderate symptoms causing greater than minimal interference with usual activities
** severe symptoms causing inability to perform usual activities
†† includes at least any of the following: vomiting, diarrhoea, loss of appetite, nausea and abdominal pain

Table S25: Details of the two patients with grade 3 (severe) adverse events

N#	Study arm	Country	Age (years)	Sex	Event	Start date	End date	Relationship to study drug	Action	Outcome
1	7-day-high-dose primaquine	Cambodia	26	male	Vertigo	On day of enrolment (day 0)	Not recorded	Probably related	Primaquine ceased	Recovered
2	Tafenoquine	Pakistan	19	female	Severe vomiting	Day 1	Day 2	Unlikely related	Symptomatic management	Recovered

Table S26: Details of the four patients with serious adverse events (SAE)

N#	Study arm	Country	Age (years)	Sex	Event details,	Relationship to study drug	Action	Outcome
1	7-day-high-dose primaquine	Cambodia	27	male	Worsening nausea and vomiting after presenting with vomiting at enrolment, and following initial study drug intake on day 1.	Probably	Hospitalization and treatment with antiemetics for 1 day	Recovered
2	7-day-high-dose primaquine	Pakistan	46	male	Patient with known Gilbert syndrome presented with persistent nausea and vomiting on day 6.	Possibly	Admitted to hospital for 48h	Recovered
					Re-presentation on day 16 with jaundice, nausea, and vomiting.		Admission to hospital for 2 days	Recovered
3	14-day-low-dose primaquine	Pakistan	45	female	History of hypertension, Myocardial infarction at Month 4	Unrelated	NA	Fatal
4	7-day-high-dose primaquine	Indonesia	57	female	Appendicitis at day 35 visit.	Unrelated	Appendectomy	Recovered
