# Supplementary material for: Effectiveness and safety of 7-day high-dose primaquine and single-dose tafenoquine versus 14-day low-dose primaquine in patients with Plasmodium vivax malaria (EFFORT): a multicentre, open-label, randomised, controlled, superiority trial
Source: Lancet Infect Dis. 2026 Jun;26(6):614–26. doi: 10.1016/S1473-3099(25)00729-7 (PMC13215974; doi:10.1016/S1473-3099(25)00729-7)
Supplement: Supplementary appendix 2 [file mmc2.pdf]

THE LANCET
Infectious Diseases

Supplementary appendix 2

This appendix formed part of the original submission and has been peer reviewed. We post it as supplied by the authors.

Supplement to: Degaga TS, Pasaribu AP, Tripura R, et al. Effectiveness and safety of 7-day high-dose primaquine and single-dose tafenoquine versus 14-day low-dose primaquine in patients with *Plasmodium vivax* malaria (EFFORT): a multicentre, open-label, randomised, controlled, superiority trial. *Lancet Infect Dis* 2026; published online Feb 11. https://doi.org/10.1016/S1473-3099(25)00729-7.

STUDY PROTOCOL	
**TITLE:**	EFFectiveness Of novel approaches to Radical cure with Tafenoquine and primaquine (EFFORT)- A randomized controlled trial in P. vivax patients
**VERSION:**	**3.3**
**VERSION DATE:**	14th September 2023
**SPONSOR:**	Menzies School of Health Research<br>Darwin (Royal Darwin Hospital Campus)<br>John Mathews Building (JMB)<br>Building 58, Royal Darwin Hospital Campus<br><br>TEL: (08) 8946 8600

PRINCIPAL INVESTIGATOR DECLARATION		
I, the undersigned, have read and understood the protocol specified below and agree on its content. I agree to perform and conduct the study as described in the protocol and in accordance with the relevant laws/regulations and standards outlined in the Clinical Trial Agreement.		
**Name:**<br>Associate Professor<br>Kamala Thriemer	**Signature:**	**Date:**<br>26th April 2023

This document is CONFIDENTIAL and shall not be disclosed, disseminated, copied or used, without a written permission.

Synopsis

**Title**	**Effectiveness of novel approaches to radical cure with tafenoquine and primaquine - a randomized controlled trial in *P. vivax* patients**
Design	Health care facility based, randomized, controlled, open label, superiority trial with 3 arms
Intervention arms	Patients are treated with schizontocidal treatment plus high dose PQ (total dose 7 mg/kg) unsupervised over 7 days (PQ7)
	Patients are treated with schizontocidal treatment plus a single dose of Tafenoquine (TQ)
Control arm	Patients are treated with schizontocidal treatment plus low dose PQ (total dose 3.5mg/kg) unsupervised over 14 days (PQ14)
Aims	• To assess the effectiveness of a short-course of high dose primaquine (total dose 7mg/kg given unsupervised over 7 days) compared to the current standard low dose primaquine regimen (total dose 3.5mg/kg given unsupervised over 14 days).<br>• To assess the effectiveness of tafenoquine (single dose of 300mg) compared to the short-course high dose primaquine regimen.<br>• To assess the safety of tafenoquine compared to the high and low dose primaquine regimens.<br>• To assess the cost-effectiveness and feasibility of high dose primaquine and tafenoquine compared to the current low dose primaquine regimen
Primary outcome	The incidence risk (time to first event) of any *P. vivax* parasitaemia during the 6-month follow up period as determined by microscopy compared between the PQ7 arm and the control arm (PQ14).
Secondary effectiveness outcomes	• The incidence risk (time to first event) of any *P. vivax* parasitaemia during the 6 months follow up period as determined by microscopy compared between PQ7 and TQ arms<br>• The incidence risk (time to first event) of symptomatic *P. vivax* parasitaemia during the 6 months follow up period as determined by microscopy compared between TQ and the control arm (PQ14).<br>• The incidence risk (time to first event) of symptomatic *P. vivax* parasitaemia during the 6-month follow up period as determined by microscopy compared between the PQ7 arm and the control arm (PQ14).<br>• The incidence risk (time to first event) of any *P. vivax* parasitaemia during the 6 months follow up period as determined by microscopy compared between PQ7 and TQ arms<br>• The incidence risk (time to first event) of any *P. vivax* parasitaemia during the 6 months follow up period as determined by microscopy compared between PQ14 and TQ arms<br>• The incidence rate (events per person-time) of any *P. vivax* parasitaemia during the 6 months follow up period as determined by microscopy compared between the PQ7 and PQ14<br>• The incidence rate (events per person-time) of symptomatic *P. vivax* parasitaemia during the 6 months follow up period as determined by microscopy compared between the PQ7 and TQ<br>• The incidence rate (events per person-time) of symptomatic *P. vivax* parasitaemia during the 6 months follow up period as determined by microscopy compared between the PQ14 and TQ<br>• The incidence rate (events per person-time) of any *P. vivax* parasitaemia during the 6 months follow up period as determined by microscopy compared between the PQ7 and PQ14<br>• The incidence rate (events per person-time) of any *P. vivax* parasitaemia during the 6 months follow up period as determined by microscopy compared between the PQ7 and TQ<br>• The incidence rate (events per person-time) of any *P. vivax* parasitaemia during the 6 months follow up period as determined by microscopy compared between the PQ14 and TQ

Safety outcomes	• The incidence risk of severe anaemia (Hb < 5g/dl) or moderate (≥5g/dl and <7g/dl) anaemia within 3 days of starting treatment and/or requiring blood transfusion within the 6 months follow up period.<br>• The incidence risk of an acute drop in Hb of >25% to <7g/dl within 3 days of starting treatment<br>• The number and proportion of adverse events detected on day 3 that triggered discontinuation or cessation of PQ treatment.<br>• The number and proportion of gastro-intestinal adverse event in all arms<br>• The number and proportion of neuropsychiatric adverse events in the TQ arm<br>• The number and proportion of adverse and serious adverse events in each arm within 42 days after start of treatment.
Inclusion criteria	• *P. vivax* peripheral parasitaemia (mono-infection) or (for Indonesia only) mixed infection (P. vivax and P. falciparum as determined by microscopy<br>• G6PD normal status (G6PD activity ≥ 70% of the adjusted male median as determined by the Biosensor™ (SD Bioline, ROK))<br>• Fever (temperature ≥37.5ºC) or history of fever in the preceding 48 hours<br>• Age ≥18 years (≥16 years in Indonesia)<br>• Written informed consent<br>• Living in the study area and willing to be followed for six months
Exclusion criteria	• Danger signs or symptoms of severe malaria<br>• Anaemia (defined as Hb <8g/dl)<br>• Pregnant or lactating females<br>• Regular use of drugs with haemolytic potential<br>• Known hypersensitivity to any of the study drugs
Follow up schedule	Day 0, 3, 7 &14 (TQ arm only), 21, 28, 35, 42 followed by<br>Month 2, 3, 4, 5, 6
Study Sites	• Arba Minch General Hospital, Ethiopia<br>• Kravanh District Hospital, Pursat, Cambodia<br>• Siem Pang Health center, Stung Treng, Cambodia<br>• Chambak Health Center, Kampong Speu, Cambodia<br>• Primary health care in Batubara, Sumatera, Indonesia<br>• Khidmat-e-Alam Medical center, Nazimabad, Pakistan<br>• Thatta Civil Hospital, Thatta, Sindh province, Pakistan
Sample size	A sample size of 720 participants (240 in each arm and allowing for 10% lost to follow-up) will have 95% power to detect a reduction in the risk of recurrence of symptomatic *P. vivax* parasitaemia (over 6 months follow-up), from 36% in the PQ14 arm to 20% in the PQ7 arm with two-sided alpha of 0.025. This sample size will also provide 80% power to detect a reduction in the risk of recurrence of *P. vivax* parasitaemia from 33% in the TQ arm to 20% in the PQ7 arm (secondary outcome #1).
Add-on studies	A Cost-effectiveness analysis will compare the following options from the healthcare provider and societal perspectives: (i) usual care in each country (PQ14 with/ without qualitative G6PD screening), (ii) PQ7 following quantitative G6PD screening, (iii) TQ following quantitative G6PD screening.<br>The feasibility assessment will focus on two aspects: (i) the use of the Biosensor™ for G6PD measurement and (ii) the day 3 clinical review. In addition, an assessment of accurate usage of the biosensor with staff at different levels of proficiency will be conducted in Cambodia only.<br>Assessment of routine radical cure roll out in Cambodia: (i) use of quantitative Biosensor™ for vivax treatment and (ii) use of community health worker follow-up to ensure adherence to radical cure and patient safety.

List of Investigators

Menzies Team	Name	Institution
Principal Investigator	Associate Professor Kamala Thriemer	Menzies School of Health Research
Co-Investigator	Prof Ric Price	Menzies School of Health Research
Co-Investigator	Dr Benedikt Ley	Menzies School of Health Research
Co-Investigator	Dr Angela Devine	Menzies School of Health Research
Co-Investigator	Dr Rob Commons	Menzies School of Health Research
**University of Melbourne Team**		
Co-Investigator	Dr Amalia Karahalios	University of Melbourne
Co-Investigator	Prof Julie Simpson	University of Melbourne
**Cambodia Site Team**		
Site PI	Dr Lek Dysoley	Cambodian National Malaria Program
Co-Investigator	Dr Lorenz von Seidlein	Mahidol Oxford Research Unit (MORU)
Co-Investigator	Prof Arjen Dondorp	Mahidol Oxford Research Unit (MORU)
Co-Investigator	Dr Thomas Peto	Mahidol Oxford Research Unit (MORU)
Co-Investigator	Dr Rupam Tripura	Mahidol Oxford Research Unit (MORU)
Co-Investigator	Dr James Callery	Mahidol Oxford Research Unit (MORU)
Co-Investigator	Dr Huy Rekol	Cambodian National Malaria Program
**Ethiopian site team**		
Site PI	Dr Tamiru Shibiru	University College of Medicine and Health Science, Arba Minch University
Co-Investigator	Prof Asrat Hailu Mekuria	Addis Abeba University
Co-Investigator	Dr Ashenafi Asseffa	Ethiopian Public Health Institute
Co-Investigator	Dr Adugna Woyessa	Ethiopian Public Health Institute
Co-Investigator	Dr Rodas Temesgen	University College of Medicine and Health Science, Arba Minch University
**Indonesian site team**		
Site PI	Assistant Professor Ayodhia Pasaribu Pitaloka	Medical Faculty, Universitas Sumatera Utara
Pakistan site team		
Site PI	Dr M Asim Beg	Aga Khan University, Karachi, Pakistan
Co-Investigator	Dr Najia Ghanchi	Aga Khan University, Karachi, Pakistan
Co-Investigator	Dr Momin Kazi	Aga Khan University, Karachi, Pakistan
Co-Investigator	Dr Farah Qamar	Aga Khan University, Karachi, Pakistan

Amendment history

Amendment number	Approved Protocol version	Date	HREC	Other approvals	Changes
	1.0	30. April 2020	Australia:<br>NTG HREC 2020-3694		
		11 May 2020	Ethiopia:<br>Arba Minch University Local IRB 302/12		
		30 June 2020	Indonesia University Ethics: 177/KEP/USU/2020		
	1.1	23 October 2020:	Ethiopia: Ministry of Science and Higher Education National Ethics: SRA/14.1/5311/20		• *Ethiopia only:* Clarification end points, duration of sample storage
	2.0	06 August 2020	Cambodia:<br>Ox-Trec 39-20		
		12 November 2020	National Ethics Committee for Health Research: 283 NECHR		
1	2.1	28 January 2021	Amendment Approved: Australia NTG HREC<br>02 February 2021		• Addition of: EQ-5D-5L questionnaire for all sites<br>• Addition of PI signature page
2	2.2	August 2021	Amendment Approved Australian NTG HREC 12th August 2021		• Aims and outcomes in the synopsis updated to align with objectives and endpoints in the protocol.<br>• Reference added to BPOM clinical trial reporting guidelines for SAE for the Indonesia site.<br>• Appendix: Treatment tablets, change TQ tablet dose used from 150 mg to 100 mg per tablet<br>• Change Indonesia site to Primary Health care in Batubara<br>• Minor changes to ICF in line with BPOM requirements in Indonesia

					• Addition of two site in Pakistan including additional investigators
3	3.0	Feb 2022	Amendment Approved Australian NTG HREC 15th March 2022		• Additional details on qualitative work to assess quantitative G6PD testing and Day 3 visit.<br>• Addition of assessment of routine radical cure interventions in Cambodia at additional sites.<br>• Expansion of Day 3 visit feasibility outside of EFFORT study staff in Cambodia.
	3.1	June 2022	Approved Australian NTG HREC 27th June 2022		• Adjust the inclusion criteria to include minors above 16 years in Indonesia<br>• Include mixed infection in Indonesia only.<br>• Additional site in Cambodia
	3.2	26th April 2023	Approved Australian NTG HREC 5th May 2023		• Adjust the primary and secondary endpoints to align with the updated Statistical Analysis Plan<br>• Inclusion of an interim analysis at 50% sample size<br>• Clarification on TQ dosing chart
	3.3	14th September 2023	Approved Australian NTG HREC 18th September 2023		• Updating Primaquine dosing chart to include dosage for 35-<45kgs.<br>• Adding Pakistan as one of the sites where costing data for cost-effectiveness assessment will be collected.

Contents

STUDY PROTOCOL 1

TITLE: 1

VERSION: 1

3.1 1

VERSION DATE: 1

June 2022 1

SPONSOR: 1

Synopsis 2

List of Investigators 4

Amendment history 5

1. Background and Rationale 11

1.1 The importance of targeting *P. vivax* in control programs 11

1.2 Current evidence on the efficacy of high and low dose Primaquine regimens 11

1.3 Poor effectiveness of 14 days primaquine 12

1.4 Current evidence for the anti-relapse efficacy of tafenoquine 13

1.5 The safety profiles of different radical cure options 13

1.6 Cost-effectiveness of radical cure 13

1.7 Rationale of the proposed clinical trial 14

2. Study Objectives 14

3. Study sites 14

3.1 Ethiopia 14

3.2 Indonesia 15

3.3 Cambodia 15

3.4. Pakistan 16

4. Study Preparation 16

5. Study design 16

5.1 Trial Design 16

5.2 Inclusion Criteria 16

5.3 Exclusion Criteria: 17

5.4 Randomization 17

6. Treatment 17

6.1 Treatment of acute vivax parasitaemia 17

6.2 Rescue treatment 18

7. Follow up 18

8. Study procedures 18

8.1 Enrolment 18
8.2 Study visits 19
Day 0 - Screening 19
Day 0 - Enrolment 19
Day 3 19
Day 7 and 14 20
Days 21, 28, 35, 42 20
Months 2, 3, 4, 5, and 6 20
Recurrence 20
Table 1: Study activities 22
8.3 Pharmacovigilance 23
8.4 Safety Assessment 23
8.5 Procedures for serious adverse events (SAEs) 23
8.6 Management of patients with anaemia related adverse events 23
8.7 Discontinuation/ Withdrawal of Participants from Study Treatment 24
9. Laboratory procedures 24
9.1 Sample collection 24
9.2 Malaria Microscopy 24
9.3 Haemoglobin 24
9.4 G6PD deficiency testing 25
9.5 Urine β-HCG pregnancy test 25
9.6 Parasite molecular analysis 25
9.7 Drug levels 25
9.8 Host genotyping and RBC polymorphisms 26
9.9 Serology 26
10. Sample Size 26
11. Endpoints 27
11.1 Effectiveness endpoints 27
Primary Endpoint 27
Secondary Effectiveness Endpoints 27
12. Analysis 28
12.1 Interim analysis 29
13. Cost-effectiveness assessment 29
13.1 Data collection 29
13.2 Data analysis 30
14. Feasibility of biosensor and day 3 visit 30

15. Assessment of current routine roll out of Biosensor and Follow-Up through Village Malaria Workers (VMW) (Cambodia only) ... 32
15.1 Qualitative Data Collection ... 32
15.2 Quantitative Data Collection ... 34
16. Accuracy of biosensor use at varying levels of proficiency (Cambodia only) ... 35
17. Results dissemination preferences and evaluation ... 36
18. Trial Governance ... 36
19. Ethical considerations ... 36
19.1 Ethical Committee ... 36
19.2 Declaration of Helsinki ... 37
19.3 Informed consent ... 37
19.4 Withdrawing consent ... 37
19.5 Compensation ... 37
20. Quality assurance ... 37
21. Dissemination strategy and Publication Policy ... 38
21.1 Dissemination of results to trial participants ... 38
21.2 Dissemination of results to policy makers ... 38
21.3 Dissemination of results to the research community ... 38
22. Appendix: Justification for use of Pyramax and DHAP ... 39
23. Appendix: Treatment of G6PD deficient patients not enrolled ... 40
24. Appendix: Drugs with hemolytic potential ... 42
25. Appendix: Hb measurement with biosensor ... 43
26. Appendix: Warning signs of severe malaria ... 44
27. Appendix: Definition of severe malaria ... 45
28. Appendix: Definition of a serious adverse event ... 46
29. Appendix: Treatment tables ... 47
29.1 Appendix: Study Treatment for in the PQ14 arm ... 47
29.2 Appendix: Study Treatment in the PQ7 arm ... 47
29.3 Appendix: Study Treatment in the TQ arm ... 48
29.4 Appendix: Schizontocidal Treatment in Ethiopia and Pakistan ... 49
29.5 Appendix: Schizontocidal Treatment in Indonesia ... 49
29.6 Appendix: Schizontocidal Treatment in Cambodia ... 49
30. Appendix: Informed Consent Pre study survey to define AMM ... 50
31. Appendix: Informed Consent Sub-study Cambodia ... 53
32. Appendix: Informed consent clinical trialEFFectiveness Of novel approaches to Radical cure with Tafenoquine and primaquine (EFFORT) ... 62
33. Appendix: Informed consent feasibility study ... 68

34. Appendix: Informed Consent Forms for Cambodia KIIs/FGDs 71

35. Appendix: Informed consent forms for Observation of Quantitative G6PD testing or Village Malaria Worker Follow-Up 74

74

36. References 77

1. Background and Rationale

1.1 The importance of targeting *P. vivax* in control programs

In 2014, the governments of 18 malaria endemic countries in the Asia Pacific region committed to eliminating malaria by 2030 [1]. Achieving this ambitious milestone will require innovative and broad ranging malaria control strategies. Over the last decades, the global incidence of malaria has decreased substantially, but this decline has been far greater for *P. falciparum* than for *P. vivax* [2-4]. *P. vivax* has generally received less public health and research attention than *P. falciparum*. However there is now compelling evidence that vivax malaria is associated with significant morbidity and mortality [5-8], and policy makers recognise the importance of its control and ultimate elimination. *P. vivax* is harder to eliminate than *P. falciparum*, due to important biological differences [9]. Unlike *P. falciparum*, *P. vivax* can form dormant liver stages (hypnozoites) that can reactivate weeks to months after an acute infection (relapse). Recurrent vivax infections are associated with febrile illness and a cumulative risk of severe anaemia, morbidity and mortality [10]. Primaquine (PQ) is the only widely available antimalarial drug that can eliminate the hypnozoite stages of *P. vivax* parasite and thereby prevent relapses. More recently Tafenoquine (TQ), an 8-aminoquinoline drug with a longer half-life than PQ, was registered with the US Food and Drug Administration (FDA) and the Australian Therapeutic Goods Administration (TGA) as an alternative to PQ, however it has not yet been introduced in malaria endemic countries.

The combination of blood stage treatment (schizontocidal treatment) and a liver stage (hypnozoidical) treatment (PQ or TQ) is referred to as "radical cure". While safe in the majority of patients, PQ can induce haemolysis in patients with glucose – 6 – phosphate dehydrogenase (G6PD) deficiency [11]. The World Health Organization (WHO) recommends routine testing of G6PD deficiency prior to PQ administration, however, this is rarely available in poorly resourced communities. To mitigate this risk, many countries administer a low dose course of PQ (total dose 3.5mg/kg) extended over 14 days. When supervised this low dose regimen has an efficacy at 6 months greater than 70% in some but not all locations [12, 13], however, in reality, adherence to such a prolonged course of treatment is poor and this can result in low effectiveness [14]. Novel approaches to radical cure that are safe and effective are thus critical for the successful control and elimination of *P. vivax.*

1.2 Current evidence on the efficacy of high and low dose Primaquine regimens

Most *P. vivax* clinical trials follow patients for only 4 to 6 weeks and usually only until the first recurrence [15]. Clinical trials of this duration can define anti-relapse efficacy in equatorial areas with short relapse periodicity but underestimate the benefits of radical cure which can prevent multiple episodes of malaria. Furthermore the frequency and risk of *P. vivax* relapse varies considerably, and in some locations late relapses can occur many months after the initial infection [16]. Whilst the early anti-relapse efficacy of a (supervised) 14 day regimen of PQ at day 42 can exceed 90% [17], the longer term

benefit of PQ, through the prevention of multiple relapses, is poorly defined. Low dose regimens with a total dose of 3.5mg/kg are currently recommended in most endemic countries, although the WHO recommends a high dose regimen in areas with a high risk of relapse. In practice a high dose PQ (total dose of 7mg/kg) regimen is only part of national policy and practice in Malaysia and China (0.75mg/kg/day over 8 days) [18].

A systematic review in 2012, identified 18 studies in which schizontocidal treatment plus PQ was compared to a schizontocidal treatment alone [12]. In studies with a total dose of PQ ≤2.5 mg/kg, the risk of recurrence was similar to treatment without PQ. In twelve studies administering a PQ dose between 2.5 and 5.0 mg/kg low dose was significantly better than control regimens (OR=0.14), and in the two studies administering a dose greater than 5.0 mg/kg the odds of recurrence decreased to 0.03. This meta-analysis was limited by comparison of studies with differing durations of follow up and pooling of data from differing endpoints.

Only five published studies have compared low versus high dose PQ directly [19-23]. Although there was no difference in outcomes between different PQ doses, only two studies [19, 21] followed patients for more than 28 days, and most studies had small numbers [19, 22] limiting their power to detect a difference.

The recently completed IMPROV trial investigated whether a high dose PQ regimen (total dose 7mg/kg) administered over 7 days was non-inferior to the same total dose given as a 14 days regimen. A total of 2,388 patients were enrolled and followed for 12 months in sites in Ethiopia, Indonesia, Vietnam and Afghanistan [24, 25]. There was no significant difference in the risk of *P. vivax* recurrence between the 7 and the 14day high dose regimens and at 1 year the overall risk of recurrence was 13% following high dose PQ (7 mg/kg). At the Ethiopian IMPROV study site the risk of recurrence at 6 months was almost half compared to a low dose regimen conducted 2 years previously (10% vs 17%) [25, 26].

1.3 Poor effectiveness of 14 days primaquine

Whilst efficacy is important, effectiveness, which takes into account adherence and the difficulties of delivering treatment outside of carefully controlled trials, is the key measure needed to inform malaria elimination strategies. In resource poor settings, it is challenging to ensure good patient adherence to a 14-day treatment regimen for an acute febrile illness. This is particularly important for *P. vivax* radical cure, since efficacy is related to the total dose of PQ administered [12, 27]. A recent prospective analysis of PQ effectiveness in 48,000 patients with *P. vivax* presenting to a district hospital in Papua Indonesia, demonstrated that an unsupervised 14 day PQ regimen was only 10% effective in preventing recurrent parasitaemia within one year [14]. These observations are supported by several studies assessing adherence of 14 day PQ [28, 29] and comparing supervised versus unsupervised treatment delivery [26, 30, 31]. In a recent study in Ethiopia patients treated with unsupervised PQ were at significantly greater risk of recurrent *P. vivax* parasitaemia within 6 months of follow-up than those in which treatment was semi-supervised [26].

1.4 Current evidence for the anti-relapse efficacy of tafenoquine

Tafenoquine (TQ), is also an 8-aminoquinoline drug, but is eliminated much slower than PQ (14-28 days versus 4-6 hours). TQ is administered as a single 300mg dose in combination with a schizonticidal drug, providing considerable advantage over the 14-day course of PQ in terms of adherence. The recent published DETECTIVE TQ efficacy trial, demonstrated that 30.9% of patients had *P. vivax* recurrence at 6 months compared to 24% in the low dose PQ arm [13]. However, there were major differences in efficacy between the study sites with PQ showing slightly better efficacy than TQ in studies conducted in South East Asia.

1.5 The safety profiles of different radical cure options

In most endemic countries high dose PQ (total dose 7mg/kg) is not recommended routinely, in part because of safety concerns, and because low dose PQ is regarded as providing adequate cure. In the IMPROV study 935 of the 2388 patients enrolled were treated with a high dose of PQ (0.5 to 1mg/kg day) [25]. Only four haemolytic events occurred, one in a patient who was G6PD deficient and erroneously enrolled into the study requiring a blood transfusion; the other three patients recovered quickly on stopping trial medication. Complementary data from a Thai study demonstrated that heterozygous females with intermediate G6PD activity were at greater risk from haemolysis following a treatment with 1mg/kg PQ per day than patients who received 0.5mg/kg/day, however this was only clinically relevant in 2 patients both of whom manifested symptoms at day seven [32]. These studies suggest that rare events with a high dose of PQ can be avoided by screening patients with a quantitative G6PD assay and excluding those with an enzyme activity below 70%, but further data is required to better assess the associated risks.

TQ has been licensed for use in G6PD normal patients, defined as having a G6PD enzyme activity greater than 70%. Recent comparative studies have shown that reductions in haemoglobin concentration occur in G6PD normal patients treated with TQ or PQ and are likely the result of the underlying parasitaemia [13, 33]. Overall, declines in haemoglobin of more than 3 g per decilitre or at least 30% of baseline levels were uncommon and didn't required clinical intervention [33].

1.6 Cost-effectiveness of radical cure

Household cost data have been collected from these pivotal clinical trials , providing information for nine countries, including Ethiopia and Indonesia [34]. The collection of appropriate cost data alongside studies of efficacy and effectiveness provide parameters for cost-effectiveness models, which are critical for informing policy makers to adapt and improve national treatment guidelines. A model evaluating the cost-effectiveness of G6PD screening has been developed and parameterized for the Thai-Myanmar border. This study found that screening patients for G6PD deficiency and supervising administration of a 14-day PQ regimen therapy was cost-effective when compared to prescribing no radical cure or prescribing radical cure without screening [34]. The model has been adapted to evaluate the cost-

effectiveness of further strategies for radical cure in Afghanistan, Ethiopia, Indonesia and Vietnam[35]. Poor patient adherence to primaquine had a large impact on the results[34], indicating that TQ following a point of care quantitative G6PD test, could be a cost-effective option.

1.7 Rationale of the proposed clinical trial

There have been three major advances in the tools available to tackle *P. vivax* relapses recently: single dose tafenoquine [13, 33], short course high dose primaquine [25] and a novel quantitative point of care G6PD test [36, 37]. Whilst single dose radical cure with TQ represents a major advance, there are concerns that the pivotal Phase 3 clinical trials were designed for non-inferiority to the low dose PQ regimen. There is increasing evidence that in many locations low dose PQ is inferior to a high dose PQ regimen. Now that G6PD diagnosis can be assured and short-course high-dose PQ can be administered safely, there is an urgent need to compare the safety and effectiveness of these alternative treatment strategies. We propose a multicentred open label randomised controlled effectiveness study to compare of these three key radical cure options, their safety, cost effectiveness and feasibility.

2. Study Objectives

- To assess the effectiveness of a short-course of high dose primaquine (total dose 7mg/kg given unsupervised over 7 days) compared to the current standard low dose primaquine regimen (total dose 3.5mg/kg given unsupervised over 14 days).
- To assess the effectiveness of tafenoquine (single dose of 300mg) compared to the short-course high dose primaquine regimen.
- To assess the effectiveness of tafenoquine (single dose of 300mg) compared to the standard low dose primaquine regimen.
- To assess the safety of tafenoquine compared to the high and low dose primaquine regimens.
- To assess the cost-effectiveness and feasibility of high dose primaquine and tafenoquine compared to the current low dose primaquine regimen

3. Study sites

The study will be a multicentre study carried out in study sites in Ethiopia, Cambodia, Indonesia, and Pakistan.

3.1 Ethiopia

The Ethiopian study will be undertaken in Arba Minch General Hospital. The hospital receives patients from two health centres located in Arba Minch town, but also from other health centres in the vicinity with a catchment population of more than 160,000 and has a clinical research facility, with a capacity to admit up to 24 patients, and clinical laboratory unit. All staff are experienced in clinical research and

GCP certified. The centre is co-managed by Addis Ababa University, a collaboration with Menzies is in place since more than 5 years and the site has been part of other largescale trials (e.g. the IMPROV trial) managed by the Menzies investigators. Malaria transmission occurs year-round with a peak between December and February. G6PD deficient variants are mostly A-.

3.2 Indonesia

The Indonesian study will be conducted in Primary Health Care in Batubara. The health centre treats inpatient and outpatients with approx. 50-70 patient visits per day serving a catchment population of nearly 30,000 people. Screening and enrolment will be extended to all health facilities in Batubara, but the primary site will remain Primary Health Care. The study team is experienced in delivering high quality GCP compliant clinical trials and the team has been part of ongoing collaborations with Menzies since more than 5 years. Malaria transmission occurs year-round with a peak between September to February. G6PD variants are generally very heterogenous in Indonesia, the most common G6PD deficient variant is the Mahidol variant.

3.3 Cambodia

The Cambodian study will be conducted in two sites: Kravanh District Hospital, Pursat Province and Siem Pang Health center, Stung Treng Province. The catchment populations of the facilities are more than 50,000 people and 24,000 respectively. An additional study site will be opened at Chambak Health Center, Chambak Commune, Phnum Sruoch District, Kampong Speu Province, Cambodia. Kampong Speu province. The site had the highest *p. vivax* cases in 2021 1099/ 3959 (28%) in Cambodia with most of the cases in Chambak Commune with a population of 12,834. (MIS CNM, Cambodia)

The site staff is experienced in conducting clinical trials and will be managed through the Mahidol-Oxford Research Unit, which has a long-standing relationship with the Menzies team. Malaria transmission occurs year-round with a peak between May and October. The most common G6PD deficient variant is the Viangchan variant.

For the feasibility assessment, two additional sites will be added outside of the trial context (14. Feasibility of biosensor and day 3 visit). These two sites have also been added for the assessment of the routine use of the Biosensor G6PD test and village malaria worker follow-up. These two sites will be added in consultation with the National Center for Parasitology, Entomology and Malaria Control (CNM) based on vivax burden and other research projects operating. Additional sites may be added as appropriate.

3.4. Pakistan

The Pakistan study will be conducted at two sites: Karachi urban site will be Khidmat-e-Alam Medical center, Nazimabad is within 10 Km of Aga Khan University hospital. It is located in densely populated central distract of Karachi city with estimated population of 2,971,626. It is a small health care facility visited by the local population. The second site will be Thatta Civil Hospital, Thatta, Sindh province located approximately 100 Km east of Karachi. The total area of the district is 8,570 sq. km with an approximate population of 979,817. Malaria transmission occurs year-round with a peak between April and October. The most common G6PD deficient variant is the Mediterranean variant.

4. Study Preparation

Only patients with a G6PD activity ≥ 70% of the adjusted male median (AMM) as determined by the Biosensor™ (SD Bioline, ROK) will be eligible for enrolment into the trial [38]. In order to determine 100% G6PD activity in each site, a total of 30 non-related adult males attending the health facility will be sampled. These 30 individuals will only be included in the pre-study to calculate the local AMM if they are negative for malaria as confirmed by microscopy, no history of fever in the receding 48 hours, no history of malaria, major injury, surgery or blood transfusion within the last 6 months. Written informed consent will be requested (Appendix: Informed Consent Pre study survey to define AMM). A fingerpick sample (total volume 10µl) will be collected and G6PD activity measured in duplicate using the Biosensor. No follow up of those patients will be required.

5. Study design

5.1 Trial Design

The study is designed as a prospective parallel group randomised controlled superiority effectiveness trial of patients with uncomplicated *P. vivax* malaria.

5.2 Inclusion Criteria

- *P. vivax* peripheral parasitaemia (mono-infection) or (for Indonesia) mixed infection (P. vivax and P. falciparum as determined by microscopy
- G6PD normal status (G6PD activity ≥ 70% of the AMM [38] as determined by Biosensor
- Fever (temperature ≥37.5ºC) or history of fever in the preceding 48 hours
- Age ≥18 years (≥16 for Indonesia)
- Written informed consent
- Living in the study area and willing to be followed for six months.

5.3 Exclusion Criteria:

- Danger signs or symptoms of severe malaria [39]
- Presence of anaemia (defined as Hb <8g/dl)
- Pregnant (as determined by Urine β-HCG pregnancy test) or lactating females
- Known allergy or sensitivity to any of the study drugs
- Regular use of drugs with haemolytic potential (See Appendix: Drugs of Haemolytic Potential)

5.4 Randomization

Eligible patients who have provided written informed consent will be enrolled into the treatment arms in a ratio of 1:1:1. Randomisation will be stratified by country, using randomly permuted blocks of varying size. Sealed opaque envelopes will be prepared by an independent statistician before the study starts, to accommodate for intermittent internet connection on site. Individual envelopes will be opened only after eligibility for the study is confirmed.

6. Treatment

6.1 Treatment of acute vivax parasitaemia

Patient will receive treatment according to the treatment arm they are randomized to.

- **The control arm:** patients are treated with schizontocidal treatment plus low dose PQ (total dose 3.5mg/kg) unsupervised over 14 days (PQ14)
- **The first intervention arm:** patients are treated with schizontocidal treatment plus high dose PQ (total dose 7 mg/kg) unsupervised over 7 days (PQ7)
- **The second intervention arm:** patients are treated with schizontocidal treatment plus a single dose of Tafenoquine (TQ).

Schizontocidal treatment will be different depending on study site and current guidelines in each country: In Ethiopia schizontocidal treatment will be chloroquine as per guidelines (see: Appendix: Schizontocidal Treatment in Ethiopia), in Indonesia DHA-piperaquine as per guidelines (see: Appendix: Schizontocidal Treatment in Indonesia) which is also used to treat mixed infections which will be eligible for enrolment in Indonesia.. In Cambodia the treatment guidelines currently recommend the use of artesunate-mefloquine, however this is expected to be replaced by pyronaridine-artesunate (Pyramax) and will therefore be used in this study (see: Appendix: Schizontocidal Treatment in Cambodia). A justification for the use of those drugs can be found in Appendix: Justification for use of Pyramax and DHAP.

Treatment will be started on the day of enrolment and the first dose will be given under supervision. Patients will receive the remaining tablets for a full course of treatment to be taken at home including

instructions on how to complete the treatment and when to come back to the study facility. Detailed dosing charts for study treatment are in Appendix: Treatment tables.27 Participant are encouraged to remain in the recruitment centre after their first dose for approx. 30 min. If they vomit the first dose while still at the recruitment centre, a repeat dose should be administered. For patients who vomit the repeat dose, second-line treatment will be provided, and the patient will continue with routine follow up. No replacement doses will be provided for treatment taken at home.

6.2 Rescue treatment

Patients who fail to respond adequately to the treatment (persistent parasitaemia with asexual stages on day 3 visit or clinical deterioration with vomiting and/or signs of severity) will be given rescue treatment according to national guidelines. Patients who develop severe malaria will be admitted to hospital and treated with the recommended parenteral drug (intravenous or intramuscular artesunate or intramuscular artemether) according to national guidelines.

7. Follow up

In order to minimise observer bias patients will be reviewed only once after enrolment during their course of treatment.

A clinical review will be carried out on day 3 (±1 day) to assess whether the patients has persistent peripheral parasitaemia, ensure clinical recovery, and assess for adverse drug reactions.

After day 3 patients in the TQ arm will be reviewed on day 7 and 14 to increase safety monitoring. Patients in the PQ arms will not be visited on these days to ensure limited interaction as not to bias adherence. Afterwards all patients will be reviewed weekly from day 21 until day 42 (±2 days) and thereafter monthly until month 6 (±1 week). At each visit a peripheral blood film will be taken, but this will only be read immediately if the patient has symptoms suggestive of malaria.

Patients presenting with a recurrent vivax malaria episode before day 28 will be treated with rescue treatment as per national guidelines. Patients presenting with a recurrent vivax malaria episode at or after day 28 will be treated with the same treatment as they were allocated to at enrolment (see details: *Recurrence*). Patients presenting with falciparum malaria in the course of the follow up will be treated per national guidelines.

8. Study procedures

The study procedures are summarised in Table 1.

8.1 Enrolment

Patients attending one of the study's health centres will be screened for enrolment into the trial. If patients are eligible and willing to participate in the study, they will be asked to sign a written informed consent form for enrolment. A register will be kept of all patients who are screened for study eligibility and when the patient is not enrolled the reasons for exclusion will be documented.

8.2 Study visits

Day 0 - Screening

For screening purposes, a finger prick blood sample will be collected and used for malaria slide, Hb measurement and G6PD testing using the Biosensor. Women between the ages of 18 to 55 willing to participate will be asked to take a urinary pregnancy test. Participants with G6PD activities ≤70% will be informed of their status and will be provided with information on G6PD deficiency and its consequences in everyday life and consecutive malaria infections.

These patients will be referred either to local medical staff or will be treated within the research centre. Recommendations and guidance on how to treat these patients can be found in Appendix: Treatment of G6PD deficient patients not enrolled.

Day 0 - Enrolment

Eligible patients who provided written informed consent will be enrolled in the trial. Following enrolment, a brief questionnaire will be completed including demographic information, medical history, treatment history, history of recent blood transfusions, and a brief physical examination will be performed. 7.5ml of venous blood will be collected in all consenting participants, and 450 µl capillary blood will be collected in those who decline venous blood collection. Blood will be used for subsequent host and parasite analyses (see: Laboratory procedures). All participants will receive their first treatment dose under supervision and consecutive dosed to take at home. They will also receive instructions on how to complete the treatment and when to come back to the study facility.

Day 3

Participants will be reviewed on day 3. At this clinical review the clinic staff will determine whether the patient is tolerating the medication or whether treatment should be curtailed early. Participants will be screened for any evidence of haemoglobinuria (dark urine), gastrointestinal intolerance or adverse symptoms. A capillary blood sample will be taken for Hb /G6PD measurement and drug level measurement. If the patient has signs of significant or impending haemolysis, then his/her primaquine medication will be ceased and he/she will be followed daily until haematological recovery is assured. A detailed SOP for acute haemolysis will outline how the study team should manage suspected haematological events (sign/symptom recognition, investigations, stopping study medications and blood transfusion). In the patients who are tolerating their medication, the clinician will remind the patient to complete a full course of treatment.

Day 7 and 14

Only participants in the TQ arm will be reviewed on day 7 and 14. Participants will be screened for any evidence of haemoglobinuria (dark urine), gastrointestinal intolerance or other adverse symptoms. A capillary blood sample will be taken for drug level measurement.

Days 21, 28, 35, 42

All participants will be reviewed again on day 21 and weekly thereafter, or on any day when they have further symptoms, until day 42. A symptom questionnaire and a brief physical exam will be performed, and the patient will be assessed for any adverse events. A fingerprick sample will be collected for blood film examination. The blood film will be read immediately in all patient presenting with a symptomatic illness, otherwise the blood film will be examined later at a reference centre. In all arms a capillary sample (450µl) will be collected for Hb / G6PD measurement on day 21 and 42. In the TQ arm additional capillary samples (450µl) will be collected on day 21, 28, 35 and 42 to determine drug levels. The remaining capillary blood will be stored in a microtainer.

Months 2, 3, 4, 5, and 6

During all monthly visits a symptom questionnaire and a brief physical exam will be performed. A fingerprick sample will be collected for blood film examination. The blood film will be read immediately in all patient presenting with a symptomatic illness, otherwise the blood film will be examined later at a reference centre. In addition, a capillary sample (450µl) will be collected for Hb & G6PD measurement. The remaining capillary blood will be stored in a microtainer.

Recurrence

All participants with fever or symptoms indicative of malaria will be asked to return. A peripheral blood film will be taken and read immediately. Patients with parasites in their peripheral blood film will be asked to provide a 7.5ml of venous blood sample and for those declining venous blood collections a 450 µl capillary blood will be collected. Blood will be used for subsequent host and parasite analyses as detailed in section Laboratory procedures for details.

All patients representing with vivax malaria before day 28 will be treated with rescue treatment and continue with their follow up as scheduled. Patients presenting with falciparum malaria (alone or mixed with *P. vivax*) in Cambodia, Ethiopia, and Pakistan at any time during the follow up will be treated according to guidelines and continue their follow up as scheduled. In Indonesia this only applies to falciparum mono-infections.

Patients presenting with vivax malaria (or in Indonesia with mixed infection) at or after day 28 will be treated with the PQ or TQ regimen that they were allocated at enrolment. Before initiation of study treatment, patients will be re-screened for G6PD, Hb and for pregnancy as at enrolment. Patients meeting the initial inclusion criteria will receive treatment and will then move to a “recurrence follow up

schedule" which includes a day 3 and day 21 visit (D_R3, and D_R21) for all participants, in addition to day 7 and day 14 (D_R7 and D_R14) visits for the TQ participants only. Then they move back to their original follow up schedule. Patients will only be allowed to have 3 recurrences during their 6 months follow up and will then be treated with high dose PQ as rescue treatment.

Table 1: Study activities

Study activities	D0	D3	D7*	D14*	D21	D28	D35	D42	M2	M3	M4	M5	M6	D_R	D_R3	D_R7*	D_R14*	D_R21
Screening, enrolment & randomisation	x																	
Treatment instructions given	x													x				
Medical exam	x	x	x*	x*	x	x	x	x	x	x	x	x	x	x	x	x*	x*	x
Venous sample (Vacutainer™ ≤ 7.5mLs)	x													x				
Parasite molecular analysis	x													x				
Host genotyping / RBC polymorphisms	x													x				
Serology	x													x				
Capillary sample (Microtainer™ ≤ 450 µl)		x	x**	x**	x	x	x	x	x	x	x	x	x		x	x**	x**	x
Malaria microscopy	x	x			x	x	x	x	x	x	x	x	x	x	x			x
Hb/G6PD measurement (Biosensor)	x	x			x[1]	x		x[1]	x	x[1]	x[1]	x[1]	x	x	x			x
Drug levels		x	x*	x*	x*	x*	x*	x*							x	x*	x*	
Adverse events assessment		x	x*	x*	x	x	x	x							x	x*	x*	x
Costing questionnaire	x	x			x													
EQ-5D-5L questionnaire	x				x								x	x				x
Questionnaire result dissemination										x								

*Only in TQ arm

**Capillary sample for drug levels

x[1] Only if G6PD Biosensor used for measurement of Hb.

8.3 Pharmacovigilance

With TQ roll out in the future, a strong pharmacovigilance will be critical. We will therefore ensure close interaction with health facilities within the study area to ensure study participants who experience adverse events will be transferred for unscheduled visits to the study team. We will also share standardized reporting templates with staff from health facilities in the study area.

8.4 Safety Assessment

Adverse events should be recorded for up to 42 days after the last day of administration of study drug. All related AEs that result in a participant's withdrawal from the study, or are present at the end of the study, should be followed up until a satisfactory resolution occurs. It will be left to the Investigator's clinical judgment whether or not an AE is of sufficient severity to require stopping the participant's treatment. A participant may also voluntarily withdraw from treatment due to what he or she perceives as an intolerable AE. If either of these occurs, the participant must undergo an end of study assessment and be given appropriate care under medical supervision until symptoms cease or the condition becomes stable.

The study will focus particularly on expected adverse event such as haemolysis, GI symptoms and neuropsychiatric events.

8.5 Procedures for serious adverse events (SAEs)

All participants with SAEs (see: Appendix: Definition of a serious adverse event) will be reviewed with a standard questionnaire. Patients suspected of severe malaria or sepsis will be treated according to local hospital guidelines.

All patients with an SAE will be assessed to ascertain malaria and haematological status. Relevant investigations will be ordered according to the attending physician. All results of clinical investigations will be collated on an SAE report form.

All SAEs will be reported by the site investigator to the study coordinator within one working day of awareness who will, in turn, notify the DSMB and PI within 48 hours of their notification of the event. For the Indonesian study sites reporting will be done within 24 hours as stipulated in section VII of the BPOM clinical trial reporting guidelines. The site PI will be responsible for notifying the local Ethical Committees as appropriate. All information received for a case will be detailed on a full SAE report form.

8.6 Management of patients with anaemia related adverse events

Malaria causes parasite induced haemolysis that can be exacerbated by either PQ or TQ. All patients will be monitored for acute recovery from their initial episode of malaria, any adverse drug reactions, and subsequent recurrence of malaria. In patients suspected of acute haemolysis, the clinical judgement of the attending clinician and patient safety should always come first, regardless of the trial protocol. A detailed SOP will be developed to guide study staff on relevant procedures.

8.7 Discontinuation/ Withdrawal of Participants from Study Treatment

Each participant has the right to withdraw from the study at any time. If the participant withdraws consent or is lost to follow up for 4 or more weekly visits, the patient will be excluded/withdrawn. It will be left to the Investigator's clinical judgment whether an AE is of sufficient severity to require stopping the participant's treatment. If the participant is withdrawn due to an adverse event, the investigator will arrange for follow-up visits or telephone calls until the adverse event has resolved, and conditions stabilised.

9. Laboratory procedures

9.1 Sample collection

Capillary samples in Microtainer™ vials will be collected by finger prick on scheduled and unscheduled visits. At each visit no more than 450 µl will be collected. RBC pellets and plasma will be separated by centrifugation and stored -80°C and -20°C, respectively.

About 7.5 ml of EDTA-anticoagulated venous blood samples will be collected on D0 and day of recurrence. The sample will be white blood cell (WBC)-depleted and both red blood cell (RBC) pellets and plasma stored at -80°C and -20°C, respectively, for further laboratory analyses as below. The total volume of blood collected will be 12.5ml-20ml, a volume well within the WHO guidelines for clinical trials.

9.2 Malaria Microscopy

Slides for microscopy will be collected on all scheduled and unscheduled visits. Slides will be read immediately only if patients are symptomatic. Thick and thin films will be stained with Giemsa and read according to standard procedures by two independent readers [40]. Blood smears with discordant results (as defined by the Obare calculator) will be re-examined by a third, independent microscopist, and parasite density will be calculated by averaging the two closest counts. Slide reading will be monitored through an external quality control system. All participants positive for malaria will be informed about the diagnosis and referred for medical review and treatment according to local guidelines.

9.3 Haemoglobin

Haemoglobin (Hb) will be measured in all patients at enrolment and on day 3. Subsequently, Hb will be measured on day 21, 42, and during monthly visits and whenever clinically warranted. Hb measurement will be done using the Biosensor (see: G6PD deficiency testing). A summary of accuracy of the biosensor Hb measurement can be found in Appendix: Hb measurement with biosensor.

9.4 G6PD deficiency testing

The adjusted male median (AMM) will be defined as 100% G6PD activity in each site and will be determined before the start of the study using Biosensor readings from 30 male adults (see: Study Preparation).

In the trial, G6PD activity will be measured quantitatively using the Biosensor on day 0 for screening and again on day 3, 21, 42, and during monthly visits. Whenever a Hb measurement is done using the biosensor a G6PD results is produced and should be recorded.

9.5 Urine β-HCG pregnancy test

A urine β-HCG pregnancy test will be conducted on all women aged 18-55 (or 16-55 in Indonesia) who are screened for the study. Anyone testing positive will be excluded from the study.

9.6 Parasite molecular analysis

Sub-patent infections (PCR positive, but blood film negative) are common and can result in anaemia, increased risk of recurrent parasitaemia, and ongoing transmission. Parasite DNA will be extracted from RBC pellets for PCR analysis. PCR will be undertaken for speciation and quantitation of sub-patent infections and genotyping. DNA from samples PCR positive for malaria will undergo further molecular analysis using PCR, microsatellite markers, SNP typing or sequencing, to quantify molecular markers of drug resistance, population genetic structural analysis and geographical markers of infection, and to analyse hrp2/hrp3-deletion [41, 42]. These samples will be stored at -20ºC prior to processing. DNA/RNA extraction will be undertaken on the blood samples using the QIAamp blood midi or mini kits (Qiagen) as appropriate in accordance with the manufacturer's instructions. Where feasible whole genome sequencing of the parasite will be undertaken on the WBC depleted venous samples which meet the criteria for successful sequencing (currently a minimum of 100 ng DNA of which up to 90% should be of parasite origin). Assessment of the total DNA/RNA quantity and percentage of human DNA/RNA will be undertaken using the Qubit HS assay (Invitrogen) and quantitative real-time PCR, respectively. Whole genome sequencing will be undertaken using the Illumina Hi-Seq and/or Mi-Seq platform. All of the procedures will be undertaken at centres with proven competency to undertake these analyses.

Drug levels

Blood collected on day 3 will be used to measure the blood concentrations of TQ, PQ and its metabolites in order to correlate treatment outcome with drug concentration within treatment arms, and to assess potential drug-drug interactions by comparing blood concentrations within arms by study site as a proxy for schizontocidal treatment.
In the TQ arm, additional blood will be collected on day 7, 14, 21, 28, 35 and 42 to provide data towards a better understanding of the PK properties.
Drug concentrations will be measured at the Mahidol Oxford Research Unit, Bangkok, Thailand, or an institution with similar capacity.

9.8 Host genotyping and RBC polymorphisms

Human DNA will be extracted using QiAMP kits (Qiagen) and assessed using molecular techniques for known variants of the G6PD gene (Xq28) and the Duffy antigen/chemokine receptor (DARC). CYP2D6 polymorphisms associated with poor treatment outcome to primaquine and other red cell polymorphisms and haemoglobinopathies related to malaria susceptibility will also be assessed [44].

9.9 Serology

Several malarial antigens have been shown to elicit long-lasting antibody responses following exposure to Plasmodium. The concentration of these antibodies in the peripheral blood may therefore reflect transmission intensity in a particular area [45]. Antibody concentrations against a number of relevant antigens will be measured by indirect ELISA and Illumina™ beads, or other methods as appropriate. Respective assessments will be conducted at a reference centre with a proven track record for these procedures. Lateral flow-based serology assays for *P. falciparum* and *P. vivax* may be included as they become available.

10. Sample Size

A sample size of 720 participants (240 in each arm and allowing for 10% lost to follow-up) will have 95% power to detect a reduction in the risk of recurrence of *P. vivax* parasitaemia (over 6 months follow-up), from 36% in the PQ14 arm to 20% in the PQ7 arm with two-sided alpha of 0.025. This sample size will provide 80% power to detect a reduction in the risk of recurrence of *P. vivax* parasitaemia from 33% in the TQ arm to 20% in the PQ7 arm (secondary outcome #1). A two-sided alpha of 0.025 was chosen to penalize for the multiple testing inherent in a 3-arm trial.
The following information was used to inform the sample size:

- Previous RCTs of efficacy (i.e. supervised treatment) with a 14-day low dose treatment (control arm) of PQ found the risk of recurrence at 6 months of 20% in Ethiopia [26] and 27% in a multicenter

study [13]. Supervised treatment of high dose PQ given over 7 days reduces the risk of recurrence to 10% [46]. Efficacy data for TQ found a risk of recurrence of 33% after 6 months [13].

- Previous RCT of effectiveness (i.e. unsupervised treatment) of low dose PQ showed an increased risk of recurrence of 36% at 6 months [26]. No data are yet available on the effectiveness of high dose 7-day courses of PQ but we assume a similar (conservative) increased risk to approximately 20%. We assume that effectiveness in the TQ arm is equivalent to its efficacy as it is provided as a single dose (i.e. 33%).

Using data from the study conducted in Ethiopia [26] and the IMPROV study [25], this sample size will have 98% power to detect a change in secondary effectiveness outcome (i.e. a change in the incidence rate of any recurrent *P. vivax* from 0.25 episodes per 6 months for PQ14 to 0.09 episodes per 6 months in the PQ7) with a two-sided alpha of 0.025.

We will aim to recruit 220-250 patients at the Cambodian sites, 130-150 in Indonesia and 350 in Ethiopia, taking into account the different burden of disease and recruitment rates. We have now added recruitment for an additional 240 patients in Pakistan, this will allow to accommodate potential lower recruitment than expected in Indonesia and Cambodia. However with the additional of those sites we expect to exceed the original sample size calculation and, thus, have increased power.

11. Endpoints

11.1 Effectiveness endpoints

Primary Endpoint

The primary effectiveness outcome will the incidence risk (time to first event) of any P. vivax parasitaemia during the 6-month follow up period as determined by microscopy compared between the PQ7 arm and the control arm (PQ14).

Secondary Effectiveness Endpoints

- The incidence risk (time to first event) of any *P. vivax* parasitaemia during the 6 months follow up period as determined by microscopy compared between PQ7 and TQ arms.
- The incidence risk (time to first event) of any *P. vivax* parasitaemia during the 6 months follow up period as determined by microscopy compared between PQ14 and TQ arms.
- The incidence risk (time to first event) of any *P. vivax* parasitaemia during the 6-month follow up period as determined by microscopy compared between the PQ7 arm and the control arm (PQ14).

- The incidence risk (time to first event) of symptomatic *P. vivax* parasitaemia during the 6 months follow up period as determined by microscopy compared between PQ7 and TQ arms.
- The incidence risk (time to first event) of symptomatic *P. vivax* parasitaemia during the 6 months follow up period as determined by microscopy compared between PQ14 and TQ arms.
- The incidence rate (events per person-time) of symptomatic *P. vivax* parasitaemia during the 6 months follow up period as determined by microscopy compared between the PQ7 and PQ14.
- The incidence rate (events per person-time) of symptomatic *P. vivax* parasitaemia during the 6 months follow up period as determined by microscopy compared between the PQ7 and TQ.
- The incidence rate (events per person-time) of symptomatic *P. vivax* parasitaemia during the 6 months follow up period as determined by microscopy compared between the PQ14 and TQ .
- The incidence rate (events per person-time) of any *P. vivax* parasitaemia during the 6 months follow up period as determined by microscopy compared between the PQ7 and PQ14.
- The incidence rate (events per person-time) of any *P. vivax* parasitaemia during the 6 months follow up period as determined by microscopy compared between the PQ7 and TQ.
- The incidence rate (events per person-time) of any P. vivax parasitaemia during the 6 months follow up period as determined by microscopy compared between the PQ14 and TQ.
- The incidence risk of severe anaemia (Hb < 5g/dl) or moderate (≥5g/dl and <7g/dl) anaemia within 3 days of starting treatment and/or requiring blood transfusion within the 6 months follow up period.
- The incidence risk of an acute drop in Hb of >25% to <7g/dl within 3 days of starting treatment
- The number and proportion of adverse events detected on day 3 that triggered discontinuation or cessation of PQ treatment.
- The number and proportion of gastro-intestinal adverse event in all arms
- The number and proportion of neuropsychiatric adverse events in the TQ arm
- The number and proportion of adverse and serious adverse events in each arm within 42 days after start of treatment.

12. Analysis

Assessing patients over multiple episodes will allow analysis of both time to first recurrence (incidence risk) as well as the incidence rate of recurrences. Statistical analyses will follow a predefined statistical plan similar to our recently completed major *P. vivax* clinical trial (24) and earlier work in Ethiopia (26). To provide a pragmatic comparison of the different drug treatments, the study design will apply an intention-to-treat analysis that will be applied to both the primary and secondary outcomes. These analyses will be conducted on all patients assigned to the treatment groups as randomised, regardless of the study treatment received and the degree of adherence. For the analysis of safety outcomes, all patients

treated with any study drug will be included in the safety analysis in the treatment group that they actually received.

Incidence risks will be calculated using the Kaplan-Meier (KM) method for each trial arm as well as a comparison of the relative hazards between trial arms (Hazard Ratio (95% CI) estimated from a Cox regression analysis for the time to the first recurrent episode). Patients who are categorised as lost to follow up will be censored at the day of the last visit.

Incidence rates will be calculated by dividing the number of symptomatic *P. vivax* episodes by the number of person-years of observation (PYO) in the study population and compared between treatment arms using Poisson regression. The start date for PYO will be the day of enrolment into the study and the stop date the last visit performed (either completed study at 6 months or any last visit before loss to follow up). The period between start and stop dates for each patient will be calculated in days and divided by 365 to determine PYO, which will then be totalled for all patients.

As per guidelines (41), the extent and pattern of missing data will be reported, and a sensitivity analysis performed to investigate the impact of different missing data assumptions on the results conducted.

12.1 Interim analysis

An interim analysis will be conducted at the availability of 6 months of follow-up data for 50% of the original sample size (i.e., 360 participants, which is 50% of 720 participants). The timing of this interim analysis is expected to be in April/May 2023, which is approximately 2 years after the first patient was recruited. At this point, the study will be continuing to enrol participants. An independent Statistician will perform the interim analysis and prepare a report for the DSMB. The interim analysis was added because of the protracted start of different study sites to potentially enable analysis of three sites only. The interim analysis will be conducted for the primary effectiveness outcome and the first secondary effectiveness outcome. Country specific analyses will not be conducted for the interim analysis.

The interim analysis will allow stopping for superiority (of the primary outcome) early using a conservative Haybittle-Peto boundary with early stopping to reject the null hypothesis. If the decision is made to stop the trial early, recruitment will cease in Indonesia and Ethiopia (noting that Cambodia has finished recruitment at the time of the interim analysis) but will continue in Pakistan. By stopping the trial early, the results from the three original sites (Cambodia, Indonesia and Ethiopia) will be made available without waiting for completion of recruitment and follow-up in Pakistan. Upon completion at the study site in Pakistan those results will then be combined with the three sites using a meta-analysis approach. No other aspects of the trial will be adapted or revised based on the results of the interim analysis, and the results of the interim analysis will not be used to adjust the sample size of this trial.

13. Cost-effectiveness assessment

13.1 Data collection

Cost data will be collected during the trial enrolment and follow up periods. For Ethiopia and Indonesia, the recently completed IMPROV trial [24] has collected most of the relevant healthcare provider cost data [47]. These will be supplemented by costs specific to implementing screening via Biosensor, TQ, a day 3 visits and severe adverse events. The healthcare provider costs collected in Cambodia and Pakitan will include all non-trial malaria activities. The household costs will be collected through a short questionnaire at enrolment, day 3 and day 21. This includes costs associated with the initial visit, any treatment-seeking behaviour before the initial visit up and productivity losses, expenditure for consultation, medication, investigations or admissions, travel costs (other than those for research purposes). EQ-5D-5L surveys will be self-administered to all patients at day 0, 21 and the 6 month follow up visit. For those with a recurrence, surveys will be done at days 0 and 21.

Data analysis

The cost data will be used in conjunction with the outcomes and patient characteristics in a cost-effectiveness analysis utilising a previously developed decision tree model [34, 35]. EQ-5D-5L data will be analysed using country-specific valuations when possible [48, 49], and quality-adjusted life-years will be calculated. This model will compare the following options from the healthcare provider and societal perspectives: (i) usual care in each country (PQ14 without G6PD screening), (ii) PQ7 following quantitative G6PD screening, (iii) TQ following quantitative G6PD screening.

14. Feasibility of biosensor and day 3 visit

The feasibility assessment will be focused on two aspects: (i) the use of the Biosensor for G6PD measurement and (ii) the day 3 review. The implementation of the Biosensor will be a crucial part for the roll out of TQ. A visit on day 3 for safety review and to increase adherence in the PQ7 schedule might be a feasible option to mitigate the risk of haemolysis particularly if the treatment schedule would be used with less stringent exclusion criteria on G6PD testing at a lower activity threshold (e.g. in combination with an RDT at a 30% activity threshold).

The data collected through this sub-study will allow identifying a range of potential issues that various users will have with the diagnostic technology and will point to possible uptake scenarios, potential pitfalls and barriers to utilization and access, as well as offer insights into how to improve technology design and implementation in routine case management. These will encompass aspects such as training, workflow, staffing, deployment, technology characteristics and usability. This information will also allow the assessment of potential implications for health equity, such as generating access to radical cure, linkage to care, cutting diagnostic delay, reducing loss to follow up, cost concerns and accessibility.

Before the start of the study all study personnel will undergo the manufacturer's standard training that accompanies the installation of the Biosensor as well as currently developed further training material by PATH and FIND and complemented by a more in-depth training on materials specifically developed for this study and purpose.

During enrolment of the first 10 patients, all study personnel who perform G6PD testing will be observed by a trainer, and his/her completion of a series of pre-defined key steps will be rated as successful or not using a standardized questionnaire. The same assessment will be conducted in regular intervals throughout the entire study period and will be accompanied by a brief semi-quantitative survey on knowledge of G6PD deficiency and use of the Biosensor. Further the perceived ease of use and acceptability of the test will be assessed using standardized questionnaires with a scored rating of answers.

In follow-up meetings, we will conduct interviews and focus group discussions (FGDs) with laboratory technicians, nurses and doctors involved in the study on their experiences with and perspectives on the diagnostic technology itself, the perceived ease of use for each of the key steps required to conduct the test, its application in routine practice, and suggestions for improvements. We will also conduct observations of patient/healthcare worker interactions and of use of the biosensor.
In addition, we will interview participants with a broader range of backgrounds including, decision makers, or other actors such as technicians, other health staff, policy makers and implementers and community members who (may) interact with the test but are not directly involved in the trial.

We will also discuss opinions about and experiences with the day 3 review. The aim is to better understand the perceived usefulness of this visit, whether this would be helpful and feasible to implement in a routine setting and if this could increase safe and effective delivery of PQ and how this could be implemented. Interviews will also aim to explore perception of risk of haemolysis among different stakeholder and how this might influence implementation and policy making. Where appropriate, the interviews will also include questions about the training materials used for the Biosensor training, to evaluate their appropriateness and collect suggestions for improvement.

Additionally, observations of the trial sites of testing and treatment procedures will allow researchers to contextualise answers from these interviews. The two informed consent forms for EFFORT clinical trial personnel can be found in Appendix: Informed consent feasibility study and in Appendix 34.

15. Assessment of current routine roll out of Biosensor and Follow-Up through Village Malaria Workers (VMW) (Cambodia only)

Presently, in Cambodia the treatment guidelines for vivax malaria include 14-day low dose PQ with quantitative G6PD testing (biosensor) and this has been rolled out in some provinces. Training on use of the biosensor has been completed for referral, provincial, and district hospital staff as well as a health centre staff in locations with one or more vivax patients per month. Patients should also be followed up by VMWs in person visits on Day 3, 7, and 14.

In this sub-study we aim to evaluate how successful this roll-out has been so far and where there is need for improvement, ultimately informing the National Malaria Control Program.

15.1 Qualitative Data Collection

Key Informant Interviews (KIIs) or FGDs with patients, high risk populations, community health workers and officials, health facility staff, provincial and district level malaria officials, appropriate National Center for Parasitology, Entomology and Malaria Control (CNM) and Ministry of Health officials, and study staff will be conducted. Discussion guides will be developed and then subsequently finalized after a pilot round. KIIs and FGDs will be recorded on two devices (in case one malfunctions), in addition to handwritten notes. KIIs and FGDs will be conducted in the local language by the study team, then recording transcribed and translated.

This sub-project will investigate what challenge, if any, the rollout has faced and how quantitative G6PD testing has fit within the routine work of health facility staff at varying levels of the health system; it will also map out the diagnostic algorithm of vivax patients from diagnosis, referral, G6PD testing to follow-up from different diagnosis starting points. Reception and attitude with regards to quantitative G6PD testing will also be explored from patients and healthcare workers.

This sub-project also seeks to answer questions regarding the implementation of VMW follow-up focusing on its feasibility and attitudes, perceptions, and opinions of VMWs, health centre staff, and patients. High-risk populations, including forest-goers who are usually male between the ages of 15 and 45, will also be part of the target population. Among high-risk populations, understanding of the importance of follow-up will be explored as well as what they are told about radical cure from healthcare workers and how that affects their behaviours. Among patients, their role in follow-up and treatment outcomes will additionally be focused on. Key informant interviews will also be set up with

VMWs and patients who respectively did not conduct or receive follow-up or if FGDs are not logistically feasible. (Informed Consent Forms can be found in Appendix 34).

Direct observation of G6PD testing procedures and workflow will also be conducted in study site district hospitals and health centres. Consent will be obtained from patients prior to any observation of patient-healthcare worker interactions, as well as general health facility workflow. Observations will be non-participant and overt; however, if mistakes are being made in the interpretation of test results and hence appropriate treatment prescription, the observer may offer suggestions to correct the interpretations and treatment.

Observation of patient-physician interactions will continue until no new insights are garnered from the observations or after 15 observations—whichever comes first. Observation may be conducted by two members of the study team, one of which speaks the local language, Khmer. Observations will take place at additional sites operating routinely and at Kravanh district hospital or Siem Pang health centre depending on case load. Observation of patient-physician interactions at each site will continue until no new insights are garnered from the observations or after 15 observations—whichever comes first. There would be a total of 45 observations. Total number of observations will, however, depend on the number of cases presenting to the health facilities.

Direct non-participant overt observation of VMW follow-up will occur in health centre study site catchment areas. Observation will take place from VMW preparations to conduct the follow-up visit through the visit itself. Interaction between the VMW and patient will be observed, paying close attention to body language and discussion between the VMW and patient. If mistakes are being made in the response to any side effects or adverse events, the observer will note down such events and make suggestions. A translator may present during observation. Prior to observing follow-up visits, conversations/informational interviews will be conducting with the VMW. A total of 10 observations will be completed (ideally, 5 in a high burden catchment area and 5 in a low burden catchment area). Final number of observations will be dependent on the number of vivax malaria cases in those catchment areas. Consent for observation will be obtained from VMWs and patients at the time of the patient's diagnosis.

Observations will be recorded on paper using a note-taking grid for which general themes are pre-determined and adjusted after initial observations. Pictures will also be taken to supplement notetaking. Consent for photographs will be obtained as part of consent for observation. Any dissemination of pictures will conceal any personal information. (Informed Consent Forms can be found in Appendix 35).

15.2 Quantitative Data Collection

To complement the qualitative data, quantitative data will be gathered from the Cambodian National Malaria Information System (MIS).

To assess the appropriate use of the Biosensor, data from the Cambodia's MIS will also be gathered and analysed. Quantitative G6PD testing rates among *Plasmodium vivax* patients, rates at which health workers properly interpret G6PD testing results, as well as the rates at which appropriate radical treatment is provided according to G6PD status will obtained for analysis.

Additionally, follow-up completion rates will be collected over the course of a 12-month period (January 2022 – December 2022) allowing for assessment during both high malaria transmission season and low transmission season. Completion of follow-up will be determined based on the newly introduced malaria information system (MIS) app, and crossed check with paper records where possible. Malaria case registries will also be analysed to determine the reoccurrence of vivax malaria with and without follow-up completed based on data recorded in the MIS app, crosschecked with paper forms where possible. Recurrence will be assessed until 6 months after initial diagnosis. If unique patient identifiers are not available, establishment of recurrence will be done based on patient name, age, and village, as recorded in the malaria registries.

A data sharing agreement will be drafted and signed by the Principal Investigator, Cambodia Ministry of Health, and the Cambodian Ministry of Health and the National Center for Parasitology, Entomology, and Malaria Control (CNM) with regards to data obtained from Cambodia's national malaria surveillance system.

Definition of outcomes of interest:

The proportion of *Plasmodium vivax* patients provided quantitative G6PD testing will be calculated by dividing the number of *P.v.* patients having received G6PD testing by the number of *P.v.* patients that were eligible for G6PD testing (*i.*e., those who could receive radical cure).

The **proportion of *Plasmodium vivax* patients for which G6PD status was correctly determined** will be calculated by dividing the number of P.v. patients whose G6PD status was correctly determined based on G6PD test results by the total number of P.v patient who received a G6PD test status.

The proportion of *Plasmodium vivax* patients provided appropriate treatment after quantitative G6PD testing will be calculated by dividing the number of patients having been administered the appropriate treatment based on G6PD test result by the number of patients having received quantitative G6PD testing during the same time period.

The proportion of *Plasmodium vivax* patients with completed follow-up will be determined by dividing the number of *Plasmodium vivax* patients for which follow-up was completed according to MIS reporting by the number of *Plasmodium vivax* patients diagnosed during the same time period.

If data quality from the MIS system allows:
The recurrence rate of *Plasmodium vivax* in patients having received in person follow-up will be obtained by dividing the number of *P.v.* patients having completed follow-up and having had a subsequent diagnosis of *P.v.* by the total number of *P.v.* patients having received all follow-up visits.

The recurrence rate of *Plasmodium vivax* in patients having not received in person follow-up will be determined by dividing the number of *P.v.* patients having not completed follow-up and having had a subsequent diagnosis of *P.v.* by the total number of *P.v.* patients having not received all follow-up visits.

16. Accuracy of biosensor use at varying levels of proficiency (Cambodia only)

The aim of this sub-study is to assess whether village malaria works in rural Cambodia can accurately detect and exclude G6PD deficient individuals from 8-aminoquinoline regimens used in the radical cure of *P. vivax* infections. For this purpose, village health worker will undergo standardized training and will subsequently test a total of 250 healthy non-related adult males. In detail:

- 125 healthy volunteers will be tested in the laboratory by a village health worker and a lab technician simultaneously to ensure that the sample is not altered between both tests
- The same procedure will be repeated on another 125 healthy volunteers in a field setting where a village health worker and a lab technician simultaneously test the samples from the same volunteer

Detailed SOPs will be developed including guidance on duplicate sample preparation, the use of separate machines for the purpose of blinding the health worker and the technician to the results of each other and the use of spectrophotometry on obtained samples as quality control measure (gold standard).
All results through the biosensor will be correlated to spectrophotometry to assess whether correlation differs between 1) village health workers and lab technicians and 2) between the lab and field settings.

Written informed consent will be requested from the volunteers (Appendix: Informed Consent Sub-study Cambodia). A fingerpick sample (total volume 10µl) will be collected and G6PD activity measured in duplicate using the Biosensor and spectrophotometry. No follow up of those volunteers will be required.

17. Results dissemination preferences and evaluation

At the month 3 visit (±1 month) a short questionnaire will assess patients' preferences for study result dissemination. Patients will be asked whether they would be interested to be informed about the results of the trial at study end and if they want to be contacted by the study team once results will be available. For patients who express interest, further questions will aim to understand their preferences of dissemination.

This data will ultimately inform appropriate strategies to disseminate research findings to trial participants at each of the study sites (see: Dissemination of results to trial participants).

Following the dissemination, participants will be invited to participate in an evaluation. A mixed methods approach will be used including qualitative interviews as well as quantitative questionnaires focused around knowledge and understanding of trial results as well as satisfaction with the dissemination methods.

18. Trial Governance

The trial will have an independent Data Safety Monitoring Board (DSMB) consisting of 3-4 independent members with expertise in drug safety, clinical trials, malaria and statistics. All SAEs deemed related to the study drug will be sent to the DSMB within 48 hours of occurrence. They will meet by teleconference regularly to review the data to ensure the safety, rights and wellbeing of all trial participants.
A Trial Steering Committee (TSC) was formed before the submission of the protocol to advise on study design. The TSC will continue in an advisory role throughout the duration of the trial and during the analysis stage. The TSC consists of 3 independent members, each of the country investigators, one Menzies investigator with extensive clinical trials experience and the principal investigator.

19. Ethical considerations

19.1 Ethical Committee

This study will be submitted for formal review and approval to the relevant national ethics boards and the Institutional Review Board of the Menzies School of Health Research (HREC) and Oxford Tropical

Research Ethics Committee (OxTREC). No participant will be enrolled, or samples processed before written approval from these bodies is obtained.

19.2 Declaration of Helsinki

The study will be carried out according to the principles stated in the Declaration of Helsinki (Ethical Principles for Medical Research Involving Human Subjects) as amended in 2008, all applicable regulations and according to established international scientific standards.
Any substantial amendments to the protocol or the Informed Consent Form will also be submitted for approval to the same ECs and competent authorities and will be implemented only after approval has been obtained.

19.3 Informed consent

The information and consent forms will be translated to local language and back translated to English to ensure adequate translation. Written informed consent will be obtained from all participants. Information provided during the consenting process will include description of the sample collection procedure, aim of the study, details on the data collected, potential benefits and risks, and assurance of confidentiality for all information and results generated by the study. Information and consent form will be read out to all participants not fully literate; illiterate participants willing to participate will provide consent by a fingerprint in the presence of a witness. The information and consent forms can be found in Appendix: Informed consent clinical trial.

19.4 Withdrawing consent

All participants will be thoroughly informed about their right to withdraw consent at any time without having to provide a reason for withdrawal or having to fear negative consequences.

19.5 Compensation

Patients enrolled into the trial will not be paid to join the study. They will however be reimbursed for costs of transportation to and from the clinic for each follow up visit.

For healthy volunteers enrolled into the pre-study (see: Study Preparation) or the Cambodian sub-study (see: Feasibility of biosensor at varying levels of proficiency (Cambodia only)) as well as for health care workers in the feasibility study (see: Feasibility assessment of Biosensor and Day 3 within the study) no compensation will be provided.

20. Quality assurance

Quality assurance and monitoring will be conducted in regular intervals. Procedural and reagent controls will be provided throughout the study period. Performance of laboratory technicians is assured by on-site training and site visits. Site visits are foreseen by the investigators on a regular basis, as well as weekly e-mail or Skype communication with local study staff.
Stringed quality control for the biosensor is important as the reading determines safe inclusion into the trial. The machine contains an internal quality check (IQC) program that is run every time the machine is switched on. Whenever a machine does not pass internal IQC, an error message is generated, unless the error can be addressed immediately (i.e. low battery) the machine will be replaced until the error has been addressed and the machine passes the IQC.
The device also comes with a control chip which will be applied at least on a weekly basis. The control result is qualitative and does not allow to identify a specific error. Whenever the machine does not pass a test run with a control chip, the machine will be replaced.
As per manufacturer recommendation we plan to run control samples every time a new test kit Lot number is used. If the recommendations should change the respective procedures will be adapted.

21. Dissemination strategy and Publication Policy

21.1 Dissemination of results to trial participants

During the trial participants will be asked about their preferences for study result dissemination (see: Results dissemination preferences and evaluation). Results will inform development of appropriate dissemination strategies across all study sites.

21.2 Dissemination of results to policy makers

To achieve the transfer of knowledge into policy, locally relevant research communications will be conducted as appropriate through workshops and policy briefs under the leadership of local collaborators and through the Vivax Working Group of the Asia Pacific Malaria Elimination Network (APMEN). This established forum brings together 20 members of National Malaria Control Programs across the Asia-Pacific, with research partners, and the World Health Organisation (WHO). This forum encourages frank discussion of the findings and the implications for malaria control policies regionally that will facilitate promotion of the study findings and translation into policy where appropriate.

21.3 Dissemination of results to the research community

Research results will be communicated through open access high impact publications, and through international meetings to ensure wider knowledge transfer to other vivax endemic countries. All Investigators will be involved in reviewing drafts of the manuscripts, abstracts, press releases, and any

other publications arising from the study. Authorship will be determined in accordance with the ICMJE guidelines and other contributors will be acknowledged.

22. Appendix: Justification for use of Pyramax and DHAP

TQ has been licensed in through the US FDA in July 2018 and subsequently though the TGA in Australia for the use or radical cure of vivax malaria ideally overlapping with therapy for acute *P. vivax* infection. The original approved prescribing information (PI) allowed for use of TQ in combination with other antimalarials. Based yet unpublished results from the INSPECTOR trial (NCT02802501) in Indonesia, GSK has changed the PI to allow use with CQ only [50]. This is currently under review by the FDA.

Indonesia: Although TQ was non inferior to low dose PQ (total dose 3.5mg/kg) in the DETECTIVE study, in the GATHER pooled analysis it was shown to be inferior (not "non-inferior") [13, 51]. The INSPECTOR study was the first to explore the use of TQ with an ACT, and was undertaken in soldiers returning from Papua Province, where there is a very high risk of *P. vivax* relapse. Preliminary analysis of the INSPECTOR trial shows low efficacy of TQ in these subjects. Importantly even in the INSPECTOR study dihydro-artemisinin piperaquine (DHAP) + TQ retained some efficacy and there were no safety concerns.

The epidemiology of *P. vivax* in Sumatra appears to be different than in Papua and a low dose PQ regimen was shown to have very good efficacy (<7% risk of recurrence at 12 months) [52]. IMPROV has also demonstrated that high dose PQ in in combination with DHAP in Sumatera had excellent efficacy (<10% risk of recurrence at 12 months) [46]. Hence whilst GSK now caution that the poor efficacy of TQ may be due to lack of activity in combination with DHAP, a more likely explanation is that the dose is inadequate in populations with high risk of relapse. We expect higher TQ efficacy in Sumatera.

To ensure all study patients receive adequate radical cure, all patients recruited into EFFORT will only be allowed to have 3 recurrences during their 6 months follow up and will then treated with high dose PQ as rescue treatment. A similar approach was taken in the recent IMPROV study in Indonesia, which included a PQ placebo arm [24].

Cambodia: Pyronaridine-artesunate (Pyramax) is a newly introduced artemisinin-based combination treatment which is expected to be the first line treatment for uncomplicated malaria in Cambodia soon. From the perspective of the national malaria control programme the most relevant schizontocidal drug to partner with 4 aminoquinolines is therefore Pyramax. Studies assessing potential pharmacokinetic interactions between Pyronaridine-artesunate and PQ found the drugs were well tolerated. Adding PQ did not result in any clinically relevant pharmacokinetic alterations to pyronaridine, artesunate, or dihydroartemisinin exposures. PQ maximum plasma drug concentrations and total exposures during

coadministration with Pyramax were higher than when PQ was given alone [53]. The National Malaria Control Program has expressed clearly the need for data from this study to inform policy. The study will address these research priorities and provide the most relevant information from study participants in Cambodia receive Pyramax as schizontocidal drug.

In order to ensure the safety of participants in the TQ arm we have included additional visit in this arm to check on the well-being of the patients. As TQ is administered as a single dose such a visit does not influence the adherence and hence the effectiveness of the TQ regimen.

23. Appendix: Treatment of G6PD deficient patients not enrolled

Malaria patient with G6PD activities <70% of the AMM will not be eligible to be enrolled into the study. Those patients will be either transferred to routine care or treated by study staff depending on the capacity of the study personnel and local arrangements. Health care staff might be confused as how to treat those patients adequately given that the biosensor is not part of routine care yet and in places were testing is done routinely a qualitative test at a 30% cut off is normally used to define G6PD deficiency. This doesn't provide guidance on how to treat patients with intermediate G6PD results between 30 and 70% of the AMM. Based on the discussion with country investigators and consulting current national guidelines we therefore suggest the following guidelines for treatment of those patients. However, we would like to emphasize that this is intended as a guidance and in no means part of the study or binding for the treating physician.

	**Ethiopia**	**Cambodia**	**Indonesia**	**Pakistan**
P. vivax (or mixed infection in Indonesia) and G6PD activity ≥70%AMM + plus all other inclusion/exclusion criteria fulfilled	Enrolled in study	Enrolled in study	Enrolled in study	Enrolled in study
P. vivax (*or mixed infection* in Indonesia) and G6PD activity ≥70%AMM but not eligible to enroll	PQ14 with close follow up	PQ14	PQ14	PQ14

P. vivax (*or mixed infection* in Indonesia) and G6PD activity 30-70%AMM	PQ14 with close follow up	PQ14 only for adult males/ No radical cure for females	8 weekly PQ	8 weekly PQ
P. vivax (or mixed infection in Indonesia) and G6PD activity <30%AMM	No radical cure	No radical cure	8 weekly PQ	8 weekly PQ

24. Appendix: Drugs with hemolytic potential

Drugs with known hemolytic properties should be avoided in study participants (see exclusion criteria). Below is a non-exhaustive list to provide guidance.

**Mechanism**	**Drugs**
**Drug-induced immune hemolytic anemia**	Beta lactamase inhibitors, cefotetan (Cefotan), ceftriaxone, fludarabine, intravenous immunoglobulin, methyldopa, nonsteroidal anti-inflammatory drugs, penicillin, piperacillin
**Drug-induced thrombotic micro-angiopathic anemia**	3,4-methylenedioxymethamphetamine (Ecstasy), bupropion (Wellbutrin), chemotherapy, clopidogrel (Plavix), cocaine, cyclosporine (Sandimmune), ibuprofen, interferon, mefloquine, metronidazole (Flagyl), nitrofurantoin, quetiapine (Seroquel), quinine, simvastatin (Zocor), tacrolimus (Prograf), trimethoprim/sulfamethoxazole.
**Oxidation**	Dapsone, nitrofurantoin, phenazopyridine, recreational nitrates, ribavirin, rifampin
Based on **Phillips J**, **Henderson AC**. Hemolytic Anemia: Evaluation and Differential Diagnosis. **Am Fam Physician.** 2018 Sep 15;98(6):354-361.	

25. Appendix: Hb measurement with biosensor

The G6PD STANDARD Biosensor (SDBiosensor, South Korea) also provides an Hb reading. It will therefore be used in this study for Hb measurement during follow up visits. Its accuracy in measuring Hb has been evaluated in two studies:

i) A total of 108 venous blood samples were collected in EDTA vacutainers in rural Bangladesh, immediately stored at 4°C and shipped to a reference centre in the capital Dhaka for testing [36]. Mean delay between sample collection and testing was 23.3 hours (range: 20.8 to 26.5). Hb reading was compared to the result of a complete blood count (CBC). BSG and CBC showed a close correlation ($R2=0.792$, $p<0.001$), mean difference between both readings was 0.4g/dL (range -2.8 to 1.6).

ii) In second study in an Africa American cohort 210 anticoagulated venous and 42 anticoagulated capillary samples were compared to paired results from a Hemocue 201 (Sweden) [37]. Correlation between results from venous samples was good ($R2=0.87$), but lower when comparing results from capillary samples ($R2=0.640$). The study also included Hb measurements from frozen, anticoagulated venous samples, where correlation was $R2=0.750$.

26. Appendix: Warning signs of severe malaria

- Not able to drink/eat
- Vomiting excessively
- Recent history of convulsions
- Altered mental state
- Prostration unable to sit/stand up

27. Appendix: Definition of severe malaria

One or more of the following clinical or laboratory features classifies the patient as suffering from severe malaria (adapted from the 2010 WHO guidelines):

- Impaired consciousness or unrousable coma
- Prostration (i.e., generalized weakness so that the patient is unable walk or sit up without assistance)
- Failure to feed
- Convulsions
- Deep breathing, respiratory distress (acidotic breathing)
- Circulatory collapse or shock, systolic blood pressure < 80 mmHg in adults and <70 mmHg in children
- Clinical jaundice
- Observed or history of haemoglobinuria
- Abnormal spontaneous bleeding
- Reliable history of anuria or oliguria over the last 24 hours

If measured or done:

- Hypoglycaemia <2.2 mmol/L (or <40 mg/dl)
- Metabolic acidosis (plasma bicarbonate < 15 mmol/l)
- Hyperlactataemia (plasma lactate > 4 mmol/L)
- Renal impairment (serum creatinine > 265 µmol/l)
- Pulmonary oedema on X-ray

28. Appendix: Definition of a serious adverse event

A serious adverse event (SAE) in human drug trials is defined as any untoward medical occurrence that at any dose

- results in death,
- is life-threatening,
- requires inpatient hospitalization or causes prolongation of existing hospitalization,
- results in persistent or significant disability/incapacity,
- congenital anomaly/birth defect, or
- requires intervention to prevent permanent impairment or damage

29. Appendix: Treatment tables

29.1 Appendix: Study Treatment for in the PQ14 arm

Primaquine (PQ) (each tablet contains 15 mg PQ) will be given daily for 14 days with food (total target dose 3.5mg/kg):

Patient weight in Kg	Number of tablets per dose (using 15mg formulation)	Number of tablets per dose (using 7.5mg formulation)	Dose (mg/kg)	Total Dose (mg/kg)
35-45	0.5	1	0.17-0.21	2.39-3.09
>45-70	1	2	0.33-0.21	4.67-3.00
>70	1.5	3	<0.32	<4.44

29.1 Appendix: Study Treatment in the PQ7 arm

Primaquine (PQ) (each tablet contains 15 mg PQ) will be given daily for 7 days with food (total target dose 7mg/kg):

Patient weight in Kg	Number of tablets per dose (Using 15mg formulation)	Number of tablets per dose (using 7.5mg formulation)	Dose (mg/kg)	Total Dose (mg/kg)
35-45	3	6	1.02-1.29	7.16-9.00
>45-70	4	8	1.33-0.86	9.33-6.00
>70	6	12	<1.27	<8.87

29.2 Appendix: Study Treatment in the TQ arm

Tafenoquine (TQ) (each tablet contains 100 mg TQ) will be given as single dose with food (total target dose 5 mg/kg):

Number of tablets per dose	Total Dose (mg/kg)
3	***All participants will receive 3 tablets***

29.3 Appendix: Schizontocidal Treatment in Ethiopia and Pakistan

Chloroquine. Each tablet contains 150mg base and will be given once daily over three days (total target dose 25mg/kg):

Patient weight in Kg	Number of Tablets per day			Total dose in mg/kg
	Day 0	Day 1	Day 2	
36-50	3	2	2	21.0-29.2
>50	4	4	2	<30.0

29.4 Appendix: Schizontocidal Treatment in Indonesia

DHA-Piperaquine (DHAP): Each tablet contains 40 mg of DHA and 320 mg piperaquine and will be given once daily for three days:

Patient weight in Kg	No of tablets per dose	DHA (mg/kg) per dose	Piperaquine (mg/kg) per dose
31 to 40	2	2.0-2.5	16.0-20.6
>40 to 60	3	2.0-2.9	16.3-23.4
>60-80	4	2.0-2.7	16.0-21.3
>80	5	<2.5	>20

29.5 Appendix: Schizontocidal Treatment in Cambodia

Pyramax: Each tablet contains 180 mg of pyronaridine tetraphosphate and 60 mg of artesunate and will be given once a day for three days:

Patient weight in Kg	No of Tablets per dose	Pyronaridine (mg/kg) per dose	Artesunate (mg/kg) per dose
45 - 65 kg	3	8.3 – 12.0	2.7-4.0
>65 kg	4	8.0 – 11.1	>3.7

30. Appendix: Informed Consent Pre study survey to define AMM

EFFectiveness Of novel approaches to Radical cure with Tafenoquine and primaquine (EFFORT)

PRE-STUDY SURVEY TO DETERMINE AMM

INFORMATION SHEET FOR ADULT PARTICIPANTS

"This Is for You to Keep"

You can say NO

Principal Investigator: Dr Kamala Thriemer

Site Investigator: <Name of each participating Site PI>

Sponsor: Menzies School of Health Research

Why we speak to you

Malaria can be a serious disease if it is not treated quickly and effectively and some forms of malaria can re-occur if not treated correctly. There are two different drugs that can make sure that a specific form of malaria is fully cured (vivax malaria). However, they can cause severe side effects in the blood of some people. These people have a special form of blood (glucose-6-phosphate dehydrogenase deficiency). They should, therefore, not receive this treatment.

We have a new tool to test for glucose-6-phosphate dehydrogenase. But before using the test, we need to understand what the normal distribution of glucose-6-phosphate dehydrogenase in your community is. For this reason, we would like to collect some blood from you to and measure the activity of glucose-6-phosphate dehydrogenase in your blood.

Who is doing the study?

The study is being conducted by the Menzies School of Health Research and we are collaborating with other research centres in Asia and Africa.

What will happen to you if you join the study?

We will collect one fingerprick blood sample from you. We will only do one test on your blood to measure the activity of glucose-6-phosphate dehydrogenase.

Compensation

We cannot give you anything for your participation.

Why should I be part of this?

It is important for you and your community to understand how many people have this special form of blood. This will help us to understand who can receive primaquine treatment for malaria and who shouldn't receive it.

Confidentiality

All information that you provide will be kept secret. Your study records may be reviewed by those working on this study (e.g. research team, study monitors). The study sponsor, the auditor(s), regulatory authorities and ethics committee may also review your medical records.

Participation is voluntary

If you do not wish to participate in this study, it will NOT affect your right to receive standard health care administered at this clinic. Any time you can withdraw from the study and still receive the treatment as you would normally.

Contact Researchers

If you have any questions about this study, you may contact -------------------- or the study doctors in this clinic. In case of an emergency, you should return to this clinic or if it is after hours, present yourself to the ----------- Hospital Emergency Room and inform the doctor that you have been a participant in this study.

Who can I contact if I am not treated as I should be?

This proposal has been reviewed and approved by the following committees: Human Research Ethics Committee of the Northern Territory Department of Health and Menzies School of Health Research, Oxford Tropical Research Ethics Committee (OxTREC) and ----TBA. They are committees whose task it is to make sure that research participants are protected from harm. If you have a complaint about the study, these should be addressed to -------, the Chairperson of the -------- (local) Ethics Committee, -----. Telephone Number: -----------.

EFFectiveness Of novel approaches to Radical cure with Tafenoquine and primaquine (EFFORT)

CONSENT FORM FOR ADULT PARTICIPANTS PRE-STUDY SURVEY AMM

"This Is for You to Keep"

You can say NO

Principal Investigator: Dr Kamala Thriemer
Site Investigator:
Sponsor: Menzies School of Health Research

I, having understood all information contained in the Participant Information Sheet and this Informed Consent Form, hereby sign to give consent to participate in the study. By signing or affixing my fingerprint, I confirm the following:

- I have fully understood the above information.
- I was able to ask question that I had about the study, and all my questions have been answered.
- I have the right to withdraw at any time and this will not affect my medical care in any way.
- I voluntarily consent and offer to take part in this study.

______________________	______________________	____________
Name of participant	Signature of participant	Date (dd/dm/yy)
______________________	______________________	____________
Name of Informer / Consent Receiver	Signature	Date (dd/mm/yy)

In case the research participant is illiterate or unable to sign, fingerprint is to be stamped below:

____

Fingerprint of research participant

______________________	______________________	____________
Name of witness	Signature of witness	Date (dd/mm/yy)
______________________	______________________	____________
Name of Informer / Consent Receiver	Signature	Date (dd/mm/yy)

31. Appendix: Informed Consent Sub-study Cambodia

EFFectiveness Of novel approaches to Radical cure with Tafenoquine and primaquine (EFFORT)<br>BIOSENSOR SUBSTUDY (CAMBODIA)<br>INFORMATION SHEET FOR ADULT PARTICIPANTS<br>**“This Is for You to Keep”**<br>**You can say NO**

Principal Investigator: Dr Kamala Thriemer

Site Investigator: <Name of each participating Site PI>

Sponsor: Menzies School of Health Research

Why we speak to you

Malaria can be a serious disease if it is not treated quickly and effectively and some forms of malaria can re-occur if not treated correctly. There are two different drugs that can make sure that a specific form of malaria is fully cured (vivax malaria). However, they can cause severe side effects in the blood of some people. These people have a special form of blood (glucose-6-phosphate dehydrogenase deficiency). They should, therefore, not receive this treatment.

We have a new tool to test for glucose-6-phosphate dehydrogenase. But before using the test in routine care, we need to understand how well it works when health care workers at different levels use it. For this reason, we would like to collect some blood from you to and two different people will measure the activity of glucose-6-phosphate dehydrogenase in your blood.

Who is doing the study?

The study is being conducted by the Menzies School of Health Research and we are collaborating with other research centres in Asia and Africa.

What will happen to you if you join the study?

We will collect one fingerprick blood sample from you. We will only do one test on your blood to measure the activity of glucose-6-phosphate dehydrogenase.

Compensation

We cannot give you anything for your participation.

Why should I be part of this?

It is important to understand how well the test works when health care workers at different levels apply it.

Confidentiality

All information that you provide will be kept secret. Your study records may be reviewed by those working on this study (e.g. research team, study monitors). The study sponsor, the auditor(s), regulatory authorities and ethics committee may also review your medical records.

Participation is voluntary

If you do not wish to participate in this study, it will NOT affect your right to receive standard health care administered at this clinic. Any time you can withdraw from the study and still receive the treatment as you would normally.

Contact Researchers

If you have any questions about this study, you may contact ------------------- or the study doctors in this clinic. In case of an emergency, you should return to this clinic or if it is after hours, present yourself to the ---------- Hospital Emergency Room and inform the doctor that you have been a participant in this study.

Who can I contact if I am not treated as I should be?

This proposal has been reviewed and approved by the following committees: Human Research Ethics Committee of the Northern Territory Department of Health and Menzies School of Health Research, Oxford Tropical Research Ethics Committee (OxTREC) and ----TBA. They are committees whose task it is to make sure that research participants are protected from harm. If you have a complaint about the study, these should be addressed to -------, the Chairperson of the -------- (local) Ethics Committee, -----. Telephone Number: -----------.

EFFectiveness Of novel approaches to Radical cure with Tafenoquine and primaquine (EFFORT)

BIOSENSOR SUBSTUDY (CAMBODIA)

CONSENT FORM FOR ADULT PARTICIPANTS

"This Is for You to Keep"

You can say NO

Principal Investigator: Dr Kamala Thriemer

Site Investigator:

Sponsor: Menzies School of Health Research

I, having understood all information contained in the Participant Information Sheet and this Informed Consent Form, hereby sign to give consent to participate in the study. By signing or affixing my fingerprint, I confirm the following:

- I have fully understood the above information.
- I was able to ask question that I had about the study, and all my questions have been answered.
- I have the right to withdraw at any time and this will not affect my medical care in any way.
- I voluntarily consent and offer to take part in this study.

______________________	______________________	____________
Name of participant	Signature of participant	Date (dd/dm/yy)
______________________	______________________	____________
Name of Informer / Consent Receiver	Signature	Date (dd/mm/yy)

In case the research participant is *<u>illiterate or unable to sign</u>**, fingerprint is to be stamped below:*

____

Fingerprint of research participant

______________________	______________________	____________
Name of witness	Signature of witness	Date (dd/mm/yy)
______________________	______________________	____________
Name of Informer / Consent Receiver	Signature	Date (dd/mm/yy)

EFFectiveness Of novel approaches to Radical cure with Tafenoquine and primaquine (EFFORT)

INFORMATION SHEET FOR PARTICIPANTS < 18 YEARS (INDONESIA)

“This Is for You to Keep”

You can say NO

Note: If you are a parent or guardian of a child below 18 years old, please read “you” as “your child”.

Principal Investigator: Dr Kamala Thriemer

Site Investigator: <Name of each participating Site PI>

Sponsor: Menzies School of Health Research

Introduction

Malaria can be a serious disease if we don’t treat it quickly and effectively. There are two main types of malaria in your area: vivax malaria and falciparum malaria. Vivax malaria can hide in the liver and go back to the blood to cause repeated illness; this is called a relapse. Patients who have vivax parasites in their blood are also treated for the parasites in the liver to prevent later relapses. There is one medicine, called primaquine, which can be taken to kill the vivax malaria parasite in the liver and will reduce your risk of relapses, but currently it must be taken over 2 weeks. Researchers have found that it is also works well given over 1 week only. There is also a new treatment called tafenoquine which is a single dose.

We are conducting a study in this clinic to find out which of those treatment options works best.

You are being asked to participate in this study since you have sought treatment for vivax malaria. If you agree to participate in this study, you will be allocated by lottery to three different groups. One group will receive the usual treatment for vivax malaria over 14 days. The other group will receive a shorter treatment for 7 days and the third group will receive the new treatment. We will then ask you to come back 3 days after you started the treatment to check how you are doing.

Later we will ask you to come back weekly for a few weeks and then monthly until 6 months after the start. For you who will get Tafenoquine drug, there will be additional visit on the 7th and 14th day.

Who is doing the study?

The study is being conducted by Menzies School of Health Research together with other research centres in Asia and Africa.

What will happen to you if you join the study?

If you agree to be in the study the following procedures will happen:

1. You will be asked about your symptoms and have an examination by a doctor. For female patients, we would like you to give us a urine sample to check if you are pregnant. If you are pregnant, you cannot participate in the study.

2. We will prick your finger to check for malaria parasites, anaemia, and glucose-6-phosphate dehydrogenase (G6PD) activity. G6PD is an important factor in your blood that helps dealing with the drugs you receive. The results from the G6PD test will help to determine whether you can be enrolled in the study.

3. We would also like to take a total of about 7.5mL of blood from your arm to do the following tests:

- To examine the malaria parasite
- To look at your blood and DNA (your inherited blueprint) to see if you have any variations in the red blood molecules related to malaria and anti-malarial drugs such as G6PD
- To test how well medicines can kill the malaria parasite infecting you
- To check whether the medicine can cause any side effects
- To follow the level of the medicine in your blood
- How your body is fighting malaria

4. You will be given antimalarial tablets and asked to wait one hour to make sure you don't vomit. The rest of the treatment you should take at home.

5. Afterward should come back to the clinic for a check up on specific days (day 3, (7), (14), 21, 28, 35 and 42 and months 2,3,4,5 and 6 after treatment). At each visit, we will ask questions to find out whether you are getting better, we will also prick your finger to see whether the malaria parasites in your blood are decreasing and to check on the red cells in your blood (called haemoglobin); this to find out whether your malaria returns and to check whether your treatment has been successful.
If at these visits you have symptoms or a fever, we will read the blood film immediately to see if you have malaria in your blood. If you are well and have no symptoms, we will still check your red cells (haemoglobin), but the blood film will only be read later at the main laboratory. You won't receive malaria treatment if you don't have any symptoms. If your symptoms or fever returns you should come as soon as possible to the clinic for a blood test so we can check your blood slide immediately and treat you if you have malaria.

If your vivax malaria returns and you have symptoms, we will treat you with the same treatment and we will collect blood from your arm again to do the same tests than on the first day.

Storing blood samples

We will store some of your blood because we cannot do all the tests straightaway in this facility. If you have glucose-6-phosphate deficiency or other problems with your red cells, we will examine your genes which are responsible for these changes. Other tests on the stored blood will include examining in more detail your malaria parasites, the amount of drug in your blood, and the reaction of our body to the malaria parasites. These tests will be done in specialist laboratories overseas. The samples that are sent overseas will be anonymously labelled so that no-one can trace them back to you.

After these tests are done, we would like to continue to store your blood in case we want to do further tests or new tests on the blood that will help us understand better about malaria and glucose-6-phosphate deficiency. If we wish to do more tests, we will give the details to the ethics committee, and they will decide if these tests can be done.

What are the risks and benefits in joining this study?

Benefits

The potential benefit of being part of this study is to give you a good treatment against vivax relapses and to help find the best treatment for everybody around the world.

You will receive the results of your glucose-6-phosphate test, which can be used in the future should you need malaria treatment again.

Risks of Primaquine and Tafenoquine

The main potential risks are side-effects from the primaquine and tafenoquine. Its side-effects include nausea and abdominal pain which can be reduced by taking the medicine with food. Both drugs commonly cause weak (G6PD deficient) red cells to break apart. If you have glucose-6-phosphate deficiency, you will not be enrolled in this study. We will follow you up closely to check your red cell count so if your red cells do break apart, we can treat you quickly. This may also mean you will need a blood transfusion.

Risk of Phlebotomy

There is minor risk associated with blood withdrawal. You may feel a slight sting where the needle is inserted. Some people may get a slight bruise, which may last several days. Occasionally, people feel a little bit dizzy or faint.

Compensation

You will not be paid to join this study and you will not be charged any money for your malaria treatment or when you are examined on your follow up visits. You will be reimbursed for costs of transportation to and from the clinic for each follow up visit.

Research Related Injuries

The study is insured. We will provide and pay for any immediate medical care for study related injuries, such as a bad reaction to the drug, and refer you to any specialist treatment that may be required. The study cannot compensate you or pay for life-long or long-term care for study related injuries or for any long-term ill effects to your health.

If your doctors decide your health is placed at risk by being in this study, or you do not follow the study schedule, then they can withdraw you from the study. You are also free to leave the study at any time.

Confidentiality

All information that you provide will be kept secret. Your study records may be reviewed by those working on this study (e.g., research team, study monitors). The study sponsor, the auditor(s), regulatory authorities, and ethics committee may also review your medical records.

Participation is voluntary

If you do not wish to participate in this study, it will NOT affect your right to receive standard health care administered at this clinic. You can withdraw yourself from the study at any time without giving any reason and still receive the treatment as you would normally.

Contact Researchers

If you have any questions about this study, you may contact dr. Ayodhia Pitaloka Pasaribu, MKed(Ped), SpA(K), Ph.D(CTM) di +62 8126024392 or the study doctors in this clinic. In case of an emergency you should return to this clinic and inform the doctor that you have been a participant in this study.

Who can I contact if I am not treated as I should be?

This proposal has been reviewed and approved by the following committees: Human Research Ethics Committee of the Northern Territory Department of Health and Menzies School of Health Research, Oxford Tropical Research Ethics Committee (OxTREC) and *Komite Etik Penelitian Kesehatan Universitas Sumatera Utara*.

They are committees whose task it is to make sure that all research participants are protected from harm. If you have a complaint about the study, these should be addressed to Prof. Gontar Alamsyah Siregar, SpPD, the Chairperson of the *Komite Etik Penelitian Kesehatan Universitas Sumatera Utara*. Telephone Number: +62 618211045.

Follow Up Visit for Recurrence Infection

If you have fever or malaria symptoms, you should return to clinical site for follow up examination. Investigator will take blood for further laboratory examination. If you are confirmed to get recurrence malaria, you will be given drugs as you got previously.

EFFectiveness Of novel approaches to Radical cure with Tafenoquine and primaquine (EFFORT)

ASSENT FORM FOR PARTICIPANTS < 18 years (INDONESIA)

“This Is for You to Keep”

You can say NO

Principal Investigator: Dr Kamala Thriemer

Site Investigator: <Name of each participating Site PI>

Sponsor: Menzies School of Health Research

I, having understood all information contained in the Participant Information Sheet and this Informed Consent Form, hereby sign to give consent to participate in the study. By signing or affixing my fingerprint, I confirm the following:

- I have fully understood the above information.
- I was able to ask question that I had about the study, and all my questions have been answered.
- I have the right to withdraw at any time and this will not affect my medical care in any way.
- I voluntarily consent and offer to take part in this study.

________________________	________________________	_____________
Name of participant	Signature of participant	Date (dd/dm/yy)

________________________	________________________	_____________
Name of Informer / Consent Receiver	Signature	Date (dd/mm/yy)

In case the research participant is illiterate or unable to sign, fingerprint is to be stamped below:

____

Fingerprint of research participant

________________________	________________________	_____________
Name of witness	Signature of witness	Date (dd/mm/yy)

________________________	________________________	_____________
Name of Informer / Consent Receiver	Signature	Date (dd/mm/yy)

Additional Informed Consent

I consent to the testing of my genes for G6PD and/or other red cells abnormalities

☐ Yes ☐ No

I consent to the long-term storage of my blood samples for tests not in the protocol. I understand that permission will be sought from the ethics committee for these extra tests.

☐ Yes ☐ No

I consent to being interviewed during the study about my experiences

☐ Yes ☐ No

I consent to being contacted after the end of the study to learn about the results of the trial

☐ Yes ☐ No

Name of participant	Signature of participant	Date (dd/dm/yy)

Name of Informer / Consent Receiver	Signature	Date (dd/mm/yy)

In case the research participant is illiterate or unable to sign, fingerprint is to be stamped below:

Fingerprint of research participant

Name of witness	Signature of witness	Date (dd/mm/yy)

32. Appendix: Informed consent clinical trialEFFectiveness Of novel approaches to Radical cure with Tafenoquine and primaquine (EFFORT)

INFORMATION SHEET FOR ADULT PARTICIPANTS

“This Is for You to Keep”

You can say NO

Principal Investigator: Dr Kamala Thriemer

Site Investigator: <Name of each participating Site PI>

Sponsor: Menzies School of Health Research

Introduction

Malaria can be a serious disease if we don’t treat it quickly and effectively. There are two main types of malaria in your area: vivax malaria and falciparum malaria. Vivax malaria can hide in the liver and go back to the blood to cause repeated illness; this is called a relapse. Patients who have vivax parasites in their blood are also treated for the parasites in the liver to prevent later relapses. There is one medicine, called primaquine, which can be taken to kill the vivax malaria parasite in the liver and will reduce your risk of relapses, but currently it has to be taken over 2 weeks. Researchers have found that it is also works well given over 1 week only. There is also a new treatment called tafenoquine which is a single dose.

We are conducting a study in this clinic to find out which of those treatment options works best.

You are being asked to participate in this study since you have sought treatment for vivax malaria. If you agree to participate in this study, you will be allocated by lottery to three different groups. One group will receive the usual treatment for vivax malaria over 14 days. The other group will receive a shorter treatment for 7 days and the third group will receive the new treatment. We will than ask you to come back 3 days after you started the treatment to check how you are going. Later we will ask you to come back weekly for a few weeks and then monthly until 6 months after the start. For you who will get Tafenoquine drug, there will be additional visit on day 7th and 14th.

Who is doing the study?

The study is being conducted by Menzies School of Health Research and we are collaborating with other research centres in Asia and Africa.

What will happen to you if you join the study?

If you agree to be in the study the following procedures will happen:

6. You will be asked about your symptoms and have an examination by a doctor. For female patients, we would like you to give us a urine sample to check if you are pregnant. If you are pregnant, you cannot participate in the study.

7. We will prick your finger to check for malaria parasites, anaemia and glucose-6-phosphate dehydrogenase (G6PD) activity. G6PD is an important factor in your blood that helps dealing with the

drugs you receive. The results from the G6PD test will help to determine whether you can be enrolled in the study.

8. We would also like to take a total of about 7.5mL of blood from your arm to do the following tests:

- To examine the malaria parasite
- To look at your blood and DNA (your inherited blueprint) to see if you have any variations in the red blood molecules related to malaria and anti-malarial drugs such as G6PD
- To test how well medicines can kill the malaria parasite infecting you
- To check whether the medicine can cause any side effects
- To follow the level of the medicine in your blood
- How your body is fighting malaria

9. You will be given antimalarial tablets and asked to wait one hour to make sure you don't vomit. The rest of the treatment you should take at home.

10. Afterward should come back to the clinic for a check up on specific days (day 3, (7, 14) 21, 28, 35 and 42 and months 2,3,4,5 and 6 after treatment). At each visit, we will ask questions to find out whether you are getting better, we will also prick your finger to see whether the malaria parasites in your blood are decreasing and to check on the red cells in your blood (called haemoglobin); this in order to find out whether your malaria returns and to check whether your treatment has been successful. If at these visits you have symptoms or a fever, we will read the blood film immediately to see if you have malaria in your blood. If you are well and have no symptoms, we will still check your red cells (haemoglobin), but the blood film will only be read later at the main laboratory. You won't receive malaria treatment if you don't have any symptoms. If your symptoms or fever returns you should come as soon as possible to the clinic for a blood test so we can check your blood slide immediately and treat you if you have malaria.

If your vivax malaria returns and you have symptoms, we will treat you with the same treatment and we will collect blood from your arm again to do the same tests than on the first day.

Storing blood samples

We will store some of your blood because we cannot do all the tests straightaway in this facility. If you have glucose-6-phosphate deficiency or other problems with your red cells, we will examine your genes which are responsible for these changes. Other tests on the stored blood will include examining in more detail your malaria parasites, the amount of drug in your blood, and the reaction of our body to the malaria parasites. These tests will be done in specialist laboratories overseas. The samples that are sent overseas will be anonymously labelled so that no-one can trace them back to you.

After these tests are done, we would like to continue to store your blood in case we want to do further tests or new tests on the blood that will help us understand better about malaria and glucose-6-phosphate deficiency. If we wish to do more tests, we will give the details to the ethics committee and they will decide if these tests can be done.

What are the risks and benefits in joining this study?

Benefits

The potential benefit of being part of this study is to give you a good treatment against vivax relapses and to help find the best treatment for everybody around the world.

You will receive the results of your glucose-6-phosphate test, which can be used in the future should you need malaria treatment again.

Risks of Primaquine and Tafenoquine

The main potential risks are side-effects from the primaquine and tafenoquine. Its side-effects include nausea and abdominal pain which can be reduced by taking the medicine with food. Both drugs commonly cause weak (G6PD deficient) red cells to break apart. If you have glucose-6-phosphate deficiency, you will not be enrolled in this study. We will follow you up closely to check your red cell count so if your red cells do break apart, we can treat you quickly. This may also mean you will need a blood transfusion.

Risk of Phlebotomy

There is minor risk associated with blood withdrawal. You may feel a slight sting where the needle is inserted. Some people may get a slight bruise, which may last several days. Occasionally, people feel a little bit dizzy or faint.

Compensation

You will not be paid to join this study and you will not be charged any money for your malaria treatment or when you are examined on your follow up visits. You will be reimbursed for costs of transportation to and from the clinic for each follow up visit.

Research Related Injuries

The study is insured. We will provide and pay for any immediate medical care for study related injuries, such as a bad reaction to the drug, and refer you to any specialist treatment that may be required. The study cannot compensate you or pay for life-long or long-term care for study related injuries or for any long-term ill effects to your health.

If your doctors decide your health is placed at risk by being in this study, or you do not follow the study schedule, then they can withdraw you from the study. You are also free to leave the study at any time.

Confidentiality

All information that you provide will be kept secret. Your study records may be reviewed by those working on this study (e.g. research team, study monitors). The study sponsor, the auditor(s), regulatory authorities and ethics committee may also review your medical records.

Participation is voluntary

If you do not wish to participate in this study, it will NOT affect your right to receive standard health care administered at this clinic. You can withdraw yourself from the study at any time without giving any particular reason and still receive the treatment as you would normally.

Contact Researchers

If you have any questions about this study, you may contact ------------------- or the study doctors in this clinic. In case of an emergency you should return to this clinic or if it is after hours present yourself to

the ----------- Hospital Emergency Room and inform the doctor that you have been a participant in this study.

Who can I contact if I am not treated as I should be?

This proposal has been reviewed and approved by the following committees: Human Research Ethics Committee of the Northern Territory Department of Health and Menzies School of Health Research, Oxford Tropical Research Ethics Committee (OxTREC) and ----TBA. They are committees whose task it is to make sure that all research participants are protected from harm. If you have a complaint about the study, these should be addressed to -------, the Chairperson of the -------- (local) Ethics Committee, -----. Telephone Number: -----------.

Follow Up Visit for Recurrence Infection

If you have a fever or malaria symptoms, you should return to the clinical site for follow up examination. We will take blood for further laboratory examination. If you are confirmed to have recurrent malaria, you will be given drugs as you got previously.

EFFectiveness Of novel approaches to Radical cure with Tafenoquine and primaquine (EFFORT)

CONSENT FORM FOR ADULT PARTICIPANTS

"This Is for You to Keep"

You can say NO

Principal Investigator: Dr Kamala Thriemer

Site Investigator: <Name of each participating Site PI>

Sponsor: Menzies School of Health Research

I, having understood all information contained in the Participant Information Sheet and this Informed Consent Form, hereby sign to give consent to participate in the study. By signing or affixing my fingerprint, I confirm the following:

- I have fully understood the above information.
- I was able to ask question that I had about the study, and all my questions have been answered.
- I have the right to withdraw at any time and this will not affect my medical care in any way.
- I voluntarily consent and offer to take part in this study.

____________________	____________________	____________
Name of participant	Signature of participant	Date (dd/dm/yy)

____________________	____________________	____________
Name of Informer / Consent Receiver	Signature	Date (dd/mm/yy)

In case the research participant is *illiterate or unable to sign*, *fingerprint is to be stamped below:*

____

Fingerprint of research participant

____________________	____________________	____________
Name of witness	Signature of witness	Date (dd/mm/yy)

____________________	____________________	____________
Name of Informer / Consent Receiver	Signature	Date (dd/mm/yy)

Additional Informed Consent

I consent to the testing of my genes for G6PD and/or other red cells abnormalities

☐ Yes ☐ No

I consent to the long-term storage of my blood samples for tests not in the protocol. I understand that permission will be sought from the ethics committee for these extra tests.

☐ Yes ☐ No

I consent to being interviewed during the study about my experiences

☐ Yes ☐ No

I consent to being contacted after the end of the study to learn about the results of the trial

☐ Yes ☐ No

________________________	________________________	_____________
Name of participant	Signature of participant	Date (dd/dm/yy)
________________________	________________________	_____________
Name of Informer / Consent Receiver	Signature	Date (dd/mm/yy)

In case the research participant is <u>illiterate or unable to sign</u>, fingerprint is to be stamped below:

____

Fingerprint of research participant

________________________	________________________	______________
Name of witness	Signature of witness	Date (dd/mm/yy)
________________________	________________________	_____________
Name of Informer / Consent Receiver	Signature	Date (dd/mm/yy)

33. Appendix: Informed consent feasibility study

EFFectiveness Of novel approaches to Radical cure with Tafenoquine and primaquine (EFFORT)

Participant Information Sheet Feasibility study

"This Is for You To Keep"

You can say NO

Introduction

Malaria can be a serious disease if it is not treated quickly and effectively and some forms of malaria can re-occur if not treated correctly. Primaquine and Tafenoquine are drugs that can make sure that a specific form of malaria is fully cured (vivax malaria). However, they can cause severe side effects in the blood of some people. These people have a special form of blood called Glucose 6 Phosphate Dehydrogenase (G6PD) deficiency. Some new tests have become available that allow knowing if people have this special form of blood or not. This then helps deciding how best to treat them so that they can be fully cured of vivax malaria.

Reason for selection

You are study staff involved in the study. We believe you can provide us with useful thoughts about how easy it is to use the test and how useful a day 3 review is and how well it fits in your setting.

Procedures

In the context of the study, we will first provide you with a training on how to use a test called the STANDARD™ G6PD Biosensor. This training will include some explanations first and then you will be given the chance to practice the test a few times. We will then let you conduct the test with a volunteer and observe how you perform the different testing steps and record our observations on a questionnaire. After that, we will ask a few questions about how easy it was for you to use the test, and what your opinions are about how useful this test would be in your daily clinical work.

In follow up meetings we will ask you about experiences with and perspectives on the test. We will also hear your opinions and experiences with the day 3 review.

The expected duration of the training is 2-3 hours, then the observation of conducting the test might take 30 minutes, and finally the duration of the interview is 30 to 40 minutes. The additional follow up meetings might take about the same time.

If you do not wish to answer any of the questions, you may say so. We wish to interview you in your office, but this can place anywhere convenient to you, and no one else will be present during the interview (if you so wish).

All information recorded is considered confidential, and no one else aside from the researchers will have access to the records. The records will be kept at a safe place.

Risks and discomforts

There is a chance that you may feel uncomfortable talking about some of the topics. However, we do not wish for this to happen, and you may refuse to answer any question, if you feel it is too personal or if talking about it makes you uncomfortable.

Benefits

There will be no direct benefit to you, but your participation is likely to help us learn more about how easy it is to conduct the test and the day 3 review and how useful it is in your setting.

Incentives

You will not be provided any incentive to take part in the research.

Privacy, anonymity and confidentiality

The information that we collect from this research project will be kept confidential. Information about you that will be collected from the study will be stored in a file which will not have your name on it, but a number assigned to it. Which number belongs to which name will be kept under lock and key and will not be accessible to anyone except the researchers, research sponsors, study monitor, the auditor(s), regulatory authorities, ethic committee and members of the study team.

Right not to participate and withdraw

You do not have to take part in this research if you do not wish to do so, and this will not affect your work in the study in any way. You will still have all the benefits that you would otherwise have.

You may stop participating in the interview at any time that you wish to, without giving any reason and losing any of your rights as a staff member or otherwise.

Answering your questions

We will happily provide you further information about the study, now or at a later time point. You may communicate with the principal investigators of the study or her/his designated person at the contact address given below. We will try and answer all your questions on any test performed in the course of this study, study objectives and study procedures.

Contact persons

You are free to ask questions at any time. If later on you have additional questions about this study or in case of any injury or illness, you should contact the Principal Investigator.

EFFectiveness Of novel approaches to Radical cure with Tafenoquine and primaquine (EFFORT)

CONSENT FORM FOR FEASIBILITY STUDY

"This Is for You to Keep"

You can say NO

I have read this consent form, or someone explained it to me. My questions have been answered.

I freely agree to be in the study and agree that my interview will be audio-taped.

______________________	______________________	____________
Name of participant	Signature of participant	Date (dd/dm/yy)
______________________	______________________	____________
Name of Informer / Consent Receiver	Signature	Date (dd/mm/yy)

34. Appendix: Informed Consent Forms for Cambodia KIIs/FGDs

EFFectiveness Of novel approaches to Radical cure with Tafenoquine and primaquine (EFFORT) –Assessment of Routine & Novel Strategies
INFORMATION SHEET FOR ADULT PARTICIPANTS IN FGDs AND KIIs
“This Is for You To Keep”
You can say NO

Principal Investigator: Dr Kamala Thriemer
Site Investigator: Dr Lek Dysoley
Sponsor: Menzies School of Health Research

Why we speak to you
Malaria can be a serious disease if it is not treated quickly and effectively, and some forms of malaria can re-occur if not treated correctly (vivax malaria). Therefore, special interventions are needed for the control and elimination of vivax malaria. As a result, the national malaria control program has implemented quantitative G6PD testing before treatment as well as Village Malaria Worker (VMW) follow-up to monitor side-effects and ensure that patients take their medicine. Research is also being conducted to determine the feasibility of vivax malaria patient clinic visit. To make sure these interventions make sense and are useful we need to understand the perceptions, attitudes, opinions, and experiences of policymakers, malaria health workers, patients, and high-risk populations. So, you have been selected to participate in this interview/discussion because you are either a malaria policy maker, healthcare professional working in malaria, or have been previously diagnosed and treated for malaria or are considered high-risk for malaria infection. You were also chosen because you meet the eligibility criteria of being over 18 years of age and being willing and able to consent to participate in these discussions.

Who is doing the study?
The study is being conducted by the Menzies School of Health Research and we are collaborating with other research centres in Asia.

What will happen to you if you join the study?
You will participate in an interview/discussion potentially covering one or more of several topics, including the rollout/use of quantitative G6PD testing, village malaria worker vivax malaria patient follow-up, introduction of a day 3 clinical review visit, and haemolytic risk and risk management. We expect that the interview/discussion will last approximately an hour and a half. We ask that you do not leave the discussion before its end, and we thank you in advance for your patience and participation. Discussions will be recorded, and all information recorded is considered confidential, and no one else aside from the researchers will have access to the records. The records will be kept at a safe place.

Benefits
Your participation will help us understand how these national policies are working and what might need to improve to make sure malaria does not affect you and others. It will also inform consideration of future changes to policies.

Risks
There are no risks associated with participating in this interview/discussion. You do not have to answer questions you do not feel comfortable answering and can stop at any time. Participation or refusal to participate will not have any negative effects. In the sharing of results from our interviews and discussion anonymity of participants is guaranteed and all personal information is kept confidential.

Compensation

Compensation for travel costs will be provided as will per diems for health workers and officials per government guidelines. A token of appreciation will also be provided to patients and high-risk populations.

Confidentiality
All information that you provide will be kept confidentially. Your responses may be reviewed by those working on this study (e.g., research team, study monitors).

Participation is voluntary
If you do not wish to participate in this study, it will NOT affect your right to receive standard health care. If you withdraw from the study, there will be no negative consequences.

Contact Researchers
If you have any questions about this study, you may contact the study's principal investigator, Rupam Triputa at _______________, or the interviewer __________________ at ___________________________.

Who can I contact if I am not treated as I should be?
This proposal has been reviewed and approved by the following committees: Human Research Ethics Committee of the Northern Territory Department of Health and Menzies School of Health Research, Oxford Tropical Research Ethics Committee (OxTREC) and Cambodia's National Ethics Committee for Human Research. They are committees whose task it is to make sure that research participants are protected from harm. If you have a complaint about the study, these should be addressed to -------, the Chairperson of the -------- (local) Ethics Committee, -----. Telephone Number: -----------.

Data protection
Menzies School of Health Research is the data controller with respect to your personal data, and as such will determine how your personal data is used in the study. Menzies School of Health Research will process your personal data for the purpose of the research outlined above. Research is a task that is performed in the public interest.

**EFFectiveness Of novel approaches to Radical cure with Tafenoquine and primaquine (EFFORT) –
Assessment of Routine & Novel Strategies**

Principal Investigator: Dr Kamala Thriemer
Site Investigator: Dr Lek Dysoley
Sponsor: Menzies School of Health Research

I, having understood all information contained in the Participant Information Sheet and this Informed Consent Form, hereby sign to give consent to participate in the study. By signing or affixing my fingerprint, I confirm the following:

- I have fully understood the above information.
- I was able to ask question that I had about the study, and all my questions have been answered.
- I have the right to withdraw at any time and this will not affect my medical care in any way.
- I voluntarily consent and offer to take part in this study.

________________________	________________________	_____________
Name of participant	Signature of participant	Date (dd/dm/yy)
________________________	________________________	_____________
Name of Informer / Consent Receiver	Signature	Date (dd/mm/yy)

In case the research participant is illiterate or unable to sign, fingerprint is to be stamped below:

____

Fingerprint of research participant

________________________	________________________	_____________
Name of witness	Signature of witness	Date (dd/mm/yy)
____________________	________________________	_____________
Name of Informer / Consent Receiver	Signature	Date (dd/mm/yy)

35. Appendix: Informed consent forms for Observation of Quantitative G6PD testing or Village Malaria Worker Follow-Up

EFFectiveness Of novel approaches to Radical cure with Tafenoquine and primaquine (EFFORT) –
Assessment of Routine & Novel Strategies

INFORMATION SHEET FOR ADULT PARTICIPANTS IN DIRECT OBSERVATION
"This Is for You To Keep"
You can say NO

Principal Investigator: Dr Kamala Thriemer
Site Investigator: Dr Lek Dysoley
Sponsor: Menzies School of Health Research

Why we speak to you
Malaria can be a serious disease if it is not treated quickly and effectively, and some forms of malaria can re-occur if not treated correctly (vivax malaria). Therefore, special interventions are needed for the control and elimination of vivax malaria. As a result, the national malaria control program has implemented quantitative G6PD testing before treatment as well as Village Malaria Worker (VMW) follow-up to monitor side-effects and ensure that patients take their medicine. To make sure these interventions make sense and are useful we need to a complete picture of their implementation. So, your setting and your work have been selected to be observed because you are either a healthcare professional working in malaria or have been diagnosed with vivax malaria. You were also chosen because you meet the eligibility criteria of being over 18 years of age and being willing and able to consent to being observed.

Who is doing the study?
The study is being conducted by the Menzies School of Health Research and we are collaborating with other research centres in Asia.

What will happen to you if you join the study?
Nothing different from what would routinely occur during vivax malaria diagnosis and treatment or during routine VMW follow-up would occur if you were to join the study. A researcher would be present during your visit or during VMW follow-up to obtain more information about how G6PD testing and VMW follow-up work routinely. An additional person (translator) may be present during observation. Pictures may be taken to document certain occurrences.

Benefits
Your participation will help us understand how these national policies are working and what might need to improve to make sure malaria does not affect you and others.

Risks
There are no risks associated with participating in this direct observation. Participation or refusal to participate will not have any negative effects on the clinical care you receive. Anonymity is assured in the sharing of results from our observation of participants is guaranteed and all personal information is kept confidential. Any dissemination of pictures will conceal any personal information.

Compensation
Allowing us to observe your work in your setting is entirely voluntary and there are no monetary and non-monetary incentives for this opportunity.

Confidentiality

All information that you provide will be kept confidentially. Notes from observations may be reviewed by those working on this study (e.g., research team, study monitors). Any dissemination of pictures will conceil any personal information and pictures will be stored securely.

Participation is voluntary

If you do not wish to participate in this study, it will NOT affect your right to receive standard health care. If you withdraw from the study, there will be no negative consequences.

Contact Researchers

If you have any questions about this study, you may contact the study's principal investigator, Rupam Triputa at _______________, or the interviewer ___________________ at ____________________________.

Who can I contact if I am not treated as I should be?

This proposal has been reviewed and approved by the following committees: Human Research Ethics Committee of the Northern Territory Department of Health and Menzies School of Health Research, Oxford Tropical Research Ethics Committee (OxTREC) and Cambodia's National Ethics Committee for Human Research. They are committees whose task it is to make sure that research participants are protected from harm. If you have a complaint about the study, these should be addressed to -------, the Chairperson of the -------- (local) Ethics Committee, -----. Telephone Number: -----------.

Data protection

Menzies School of Health Research is the data controller with respect to your personal data, and as such will determine how your personal data is used in the study. Menzies School of Health Research will process your personal data for the purpose of the research outlined above. Research is a task that is performed in the public interest.

EFFectiveness Of novel approaches to Radical cure with Tafenoquine and primaquine (EFFORT) –
Assessment of Routine & Novel Strategies

Principal Investigator: Dr Kamala Thriemer
Site Investigator: Dr Lek Dysoley
Sponsor: Menzies School of Health Research

I, having understood all information contained in the Participant Information Sheet and this Informed Consent Form, hereby sign to give consent to participate in the study. By signing or affixing my fingerprint, I confirm the following:

- I have fully understood the above information.
- I was able to ask question that I had about the study, and all my questions have been answered.
- I have the right to withdraw at any time and this will not affect my medical care in any way.
- I voluntarily consent and offer to take part in this study.

______________________	______________________	____________
Name of participant	Signature of participant	Date (dd/dm/yy)

______________________	______________________	____________
Name of Informer / Consent Receiver	Signature	Date (dd/mm/yy)

In case the research participant is illiterate or unable to sign, fingerprint is to be stamped below:

____

Fingerprint of research participant

______________________	______________________	____________
Name of witness	Signature of witness	Date (dd/mm/yy)

______________________	______________________	____________
Name of Informer / Consent Receiver	Signature	Date (dd/mm/yy)

36. References

1. **Chairman's Statement of the 9th East Asia Summit** [http://dfat.gov.au/international-relations/regional-architecture/eas/Pages/chairmans-statement-of-the-9th-east-asia-summit.aspx]
2. Coura JR, Suarez-Mutis M, Ladeia-Andrade S: **A new challenge for malaria control in Brazil: asymptomatic Plasmodium infection--a review.** *Mem Inst Oswaldo Cruz* 2006, **101:**229-237.
3. Sattabongkot J, Tsuboi T, Zollner GE, Sirichaisinthop J, Cui L: **Plasmodium vivax transmission: chances for control?** *Trends Parasitol* 2004, **20:**192-198.
4. White MT, Yeung S, Patouillard E, Cibulskis R: **Costs and Cost-Effectiveness of *Plasmodium vivax* Control.** *Am J Trop Med Hyg* 2016, **95:**52-61.
5. Price RN, Tjitra E, Guerra CA, Yeung S, White NJ, Anstey NM: **Vivax malaria: neglected and not benign.** *Am J Trop Med Hyg* 2007, **77:**79-87.
6. Douglas NM, Pontororing GJ, Lampah DA, Yeo TW, Kenangalem E, Poespoprodjo JR, Ralph AP, Bangs MJ, Sugiarto P, Anstey NM, Price RN: **Mortality attributable to *Plasmodium vivax* malaria: a clinical audit from Papua, Indonesia.** *BMC Med* 2014, **12:**217.
7. Poespoprodjo JR, Fobia W, Kenangalem E, Lampah DA, Hasanuddin A, Warikar N, Sugiarto P, Tjitra E, Anstey NM, Price RN: **Vivax malaria: a major cause of morbidity in early infancy.** *Clin Infect Dis* 2009, **48:**1704-1712.
8. Naing C, Whittaker MA, Nyunt Wai V, Mak JW: **Is Plasmodium vivax malaria a severe malaria?: a systematic review and meta-analysis.** *PLoS Negl Trop Dis* 2014, **8:**e3071.
9. Mueller I, Galinski MR, Baird JK, Carlton JM, Kochar DK, Alonso PL, del Portillo HA: **Key gaps in the knowledge of Plasmodium vivax, a neglected human malaria parasite.** *Lancet Infect Dis* 2009, **9:**555-566.
10. Price RN, Douglas NM, Anstey NM: **New developments in Plasmodium vivax malaria: severe disease and the rise of chloroquine resistance.** *Curr Opin Infect Dis* 2009, **22:**430-435.
11. Ashley EA, Recht J, White NJ: **Primaquine: the risks and the benefits.** *Malar J* 2014, **13:**418.
12. John GK, Douglas NM, von Seidlein L, Nosten F, Baird JK, White NJ, Price RN: **Primaquine radical cure of *Plasmodium vivax*: a critical review of the literature.** *Malar J* 2012, **11:**280.
13. Llanos-Cuentas A, Lacerda MVG, Hien TT, Velez ID, Namaik-Larp C, Chu CS, Villegas MF, Val F, Monteiro WM, Brito MAM, et al: **Tafenoquine versus Primaquine to Prevent Relapse of *Plasmodium vivax* Malaria.** *N Engl J Med* 2019, **380:**229-241.
14. Douglas NM, Poespoprodjo JR, Patriani D, Malloy MJ, Kenangalem E, Sugiarto P, Simpson JA, Soenarto Y, Anstey NM, Price RN: **Unsupervised primaquine for the treatment of *Plasmodium vivax* malaria relapses in southern Papua: A hospital-based cohort study.** *PLoS Med* 2017, **14:**e1002379.
15. Commons RJ, Thriemer K, Humphreys G, Suay I, Sibley CH, Guerin PJ, Price RN: **The Vivax Surveyor: Online mapping database for *Plasmodium vivax* clinical trials.** *Int J Parasitol Drugs Drug Resist* 2017, **7:**181-190.
16. White NJ: **Determinants of relapse periodicity in Plasmodium vivax malaria.** *Malar J* 2011, **10**.
17. Commons RJ, Simpson JA, Thriemer K, Humphreys GS, Abreha T, Alemu SG, Anez A, Anstey NM, Awab GR, Baird JK, et al: **The effect of chloroquine dose and primaquine on Plasmodium vivax recurrence: a WorldWide Antimalarial Resistance Network systematic review and individual patient pooled meta-analysis.** *Lancet Infect Dis* 2018.
18. Thriemer K, Ley B, Bobogare A, Dysoley L, Alam MS, Pasaribu AP, Sattabongkot J, Jambert E, Domingo GJ, Commons R, et al: **Challenges for achieving safe and effective radical cure of *Plasmodium vivax*: a round table discussion of the APMEN Vivax Working Group.** *Malar J* 2017, **16:**141.
19. Saint-Yves IF: **Comparison of treatment schedules for Plasmodium vivax infections in the Solomon Islands.** *P N G Med J* 1977, **20:**62-65.
20. Krudsood S, Tangpukdee N, Wilairatana P, Phophak N, Baird JK, Brittenham GM, Looareesuwan S: **High-dose primaquine regimens against relapse of Plasmodium vivax malaria.** *Am J Trop Med Hyg* 2008, **78:**736-740.

21. Rajgor DD, Gogtay NJ, Kadam VS, Kocharekar MM, Parulekar MS, Dalvi SS, Vaidya AB, Kshirsagar NA: **Antirelapse Efficacy of Various Primaquine Regimens for Plasmodium vivax.** *Malar Res Treat* 2014, **2014:**347018.
22. Saravu K, Tellapragada C, Kulavalli S, Xavier W, Umakanth S, Brahmarouphu G, Srinivas NK, Channabasavaiah JP, Bava A, Saadi AV, et al: **A pilot randomized controlled trial to compare the effectiveness of two 14-day primaquine regimens for the radical cure of vivax malaria in South India.** *Malar J* 2018, **17:**321.
23. Pukrittayakamee S, Imwong M, Chotivanich K, Singhasivanon P, Day NP, White NJ: **A comparison of two short-course primaquine regimens for the treatment and radical cure of Plasmodium vivax malaria in Thailand.** *Am J Trop Med Hyg* 2010, **82:**542-547.
24. **Improving the radical cure of vivax malaria (IMPROV): a study protocol for a multicentre randomised, placebo-controlled comparison of short and long course primaquine regimens.** *BMC Infect Dis* 2015, **15**.
25. Taylor WRT, Thriemer K, von Seidlein L, al. e: **Short course primaquine for the radical cure of Plasmodium vivax malaria: a randomised placebo-controlled multicentre trial.** 2019 submitted.
26. Abreha T, Hwang J, Thriemer K, Tadesse Y, Girma S, Melaku Z, Assef A, Kassa M, Chatfield MD, Landman KZ, et al: **Comparison of artemether-lumefantrine and chloroquine with and without primaquine for the treatment of *Plasmodium vivax* infection in Ethiopia: A randomized controlled trial.** *PLoS Med* 2017, **14:**e1002299.
27. Baird JK, Rieckmann KH: **Can primaquine therapy for vivax malaria be improved?** *Trends Parasitol* 2003, **19:**115-120.
28. Khantikul N, Butraporn P, Kim HS, Leemingsawat S, Tempongko MA, Suwonkerd W: **Adherence to antimalarial drug therapy among vivax malaria patients in northern Thailand.** *J Health Popul Nutr* 2009, **27:**4-13.
29. Pereira EA, Ishikawa EA, Fontes CJ: **Adherence to Plasmodium vivax malaria treatment in the Brazilian Amazon Region.** *Malar J* 2011, **10:**355.
30. Maneeboonyang W, Lawpoolsri S, Puangsa-Art S, Yimsamran S, Thanyavanich N, Wuthisen P, Prommongkol S, Chaimongkul W, Rukmanee P, Rukmanee N, et al: **Directly observed therapy with primaquine to reduce the recurrence rate of *plasmodium vivax* infection along the Thai-Myanmar border.** *Southeast Asian J Trop Med Public Health* 2011, **42:**9-18.
31. Takeuchi R, Lawpoolsri S, Imwong M, Kobayashi J, Kaewkungwal J, Pukrittayakamee S, Puangsa-art S, Thanyavanich N, Maneeboonyang W, Day NP, Singhasivanon P: **Directly-observed therapy (DOT) for the radical 14-day primaquine treatment of Plasmodium vivax malaria on the Thai-Myanmar border.** *Malar J* 2010, **9:**308.
32. Chu CS, Bancone G, Moore KA, Win HH, Thitipanawan N, Po C, Chowwiwat N, Raksapraidee R, Wilairisak P, Phyo AP, et al: **Haemolysis in G6PD Heterozygous Females Treated with Primaquine for *Plasmodium vivax* Malaria: A Nested Cohort in a Trial of Radical Curative Regimens.** *PLoS Med* 2017, **14:**e1002224.
33. Lacerda MVG, Llanos-Cuentas A, Krudsood S, Lon C, Saunders DL, Mohammed R, Yilma D, Batista Pereira D, Espino FEJ, Mia RZ, et al: **Single-Dose Tafenoquine to Prevent Relapse of *Plasmodium vivax* Malaria.** *N Engl J Med* 2019, **380:**215-228.
34. Devine A, Parmiter M, Chu C, Bancone G, Nosten F, Price RN, Lubell Y, Yeung S: **Using G6PD Tests to Enable the Safe Treatment of Plasmodium vivax Infections with Primaquine on the Thailand-Myanmar border: a Cost-Effectiveness Analysis.** *Plos NTD under review* 2017.
35. Devine A, Howes RE, Price DJ, Moore KA, Ley B, Simpson JA, Dittrich S, Price RN: **Cost-Effectiveness Analysis of Sex-Stratified *Plasmodium vivax* Treatment Strategies Using Available G6PD Diagnostics to Accelerate Access to Radical Cure.** *Am J Trop Med Hyg* 2020.
36. Alam MS, Kibria MG, Jahan N, Thriemer K, Hossain MS, Douglas NM, Phru CS, Khan WA, Price RN, Ley B: **Field evaluation of quantitative point of care diagnostics to measure glucose-6-phosphate dehydrogenase activity.** *PLoS One* 2018, **13:**e0206331.

37. Pal S, Bansil P, Bancone G, Hrutkay S, Kahn M, Gornsawun G, Penpitchaporn P, Chu CS, Nosten F, Domingo GJ: **Evaluation of a Novel Quantitative Test for Glucose-6-Phosphate Dehydrogenase Deficiency: Bringing Quantitative Testing for Glucose-6-Phosphate Dehydrogenase Deficiency Closer to the Patient.** *Am J Trop Med Hyg* 2019, **100:**213-221.
38. Domingo GJ, Satyagraha AW, Anvikar A, Baird K, Bancone G, Bansil P: **G6PD testing in support of treatment and elimination of malaria: recommendations for evaluation of G6PD tests.** *Malar J* 2013, **12**.
39. WHO: **Guidelines for the treatment of malaria.** 3rd edition edition2015.
40. WHO: **Basic Malaria Microscopy - Part I.** In *Learner´s Guide*, vol. second edition2010.
41. Gamboa D, Ho MF, Bendezu J, Torres K, Chiodini PL, Barnwell JW, Incardona S, Perkins M, Bell D, McCarthy J, Cheng Q: **A large proportion of P. falciparum isolates in the Amazon region of Peru lack pfhrp2 and pfhrp3: implications for malaria rapid diagnostic tests.** *PLoS One* 2010, **5:**e8091.
42. Gatton ML, Dunn J, Chaudhry A, Ciketic S, Cunningham J, Cheng Q: **Implications of Parasites Lacking Plasmodium falciparum Histidine-Rich Protein 2 on Malaria Morbidity and Control When Rapid Diagnostic Tests Are Used for Diagnosis.** *J Infect Dis* 2017, **215:**1156-1166.
43. Cheng Q, Gatton ML, Barnwell J, Chiodini P, McCarthy J, Bell D, Cunningham J: **Plasmodium falciparum parasites lacking histidine-rich protein 2 and 3: a review and recommendations for accurate reporting.** *Malar J* 2014, **13:**283.
44. Lell B, May J, Schmidt-Ott RJ, Lehman LG, Luckner D, Greve B, Matousek P, Schmid D, Herbich K, Mockenhaupt FP, et al: **The role of red blood cell polymorphisms in resistance and susceptibility to malaria.** *Clin Infect Dis* 1999, **28:**794-799.
45. Stewart L, Gosling R, Griffin J, Gesase S, Campo J, Hashim R, Masika P, Mosha J, Bousema T, Shekalaghe S, et al: **Rapid assessment of malaria transmission using age-specific sero-conversion rates.** *PLoS One* 2009, **4:**e6083.
46. Taylor WRJ, Thriemer K, von Seidlein L, Yuentrakul P, Assawariyathipat T, Assefa A, Auburn S, Chand K, Chau NH, Cheah PY, et al: **Short-course primaquine for the radical cure of Plasmodium vivax malaria: a multicentre, randomised, placebo-controlled non-inferiority trial.** *Lancet* 2019.
47. Devine A, Pasaribu AP, Teferi T, Pham HT, Awab GR, Contantia F, Nguyen TN, Ngo VT, Tran TH, Hailu A, et al: **Provider and household costs of Plasmodium vivax malaria episodes: a multicountry comparative analysis of primary trial data.** *Bulletin of the World Health Organization* 2019, **97:**828-836.
48. Welie AG, Gebretekle GB, Stolk E, Mukuria C, Krahn MD, Enquoselassie F, Fenta TG: **Valuing Health State: An EQ-5D-5L Value Set for Ethiopians.** *Value Health Reg Issues* 2020, **22:**7-14.
49. Purba FD, Hunfeld JAM, Iskandarsyah A, Fitriana TS, Sadarjoen SS, Ramos-Goñi JM, Passchier J, Busschbach JJV: **The Indonesian EQ-5D-5L Value Set.** *Pharmacoeconomics* 2017, **35:**1153-1165.
50. **Change in Krintafel (tafenoquine) Label** [https://www.cdc.gov/malaria/new_info/2020/tafenoquine_2020.html]
51. Llanos-Cuentas A, Lacerda MV, Rueangweerayut R, Krudsood S, Gupta SK, Kochar SK, Arthur P, Chuenchom N, Mohrle JJ, Duparc S, et al: **Tafenoquine plus chloroquine for the treatment and relapse prevention of *Plasmodium vivax* malaria (DETECTIVE): a multicentre, double-blind, randomised, phase 2b dose-selection study.** *Lancet* 2014, **383:**1049-1058.
52. Pasaribu AP, Chokejindachai W, Sirivichayakul C, Tanomsing N, Chavez I, Tjitra E, Pasaribu S, Imwong M, White NJ, Dondorp AM: **A randomized comparison of dihydroartemisinin-piperaquine and artesunate-amodiaquine combined with primaquine for radical treatment of vivax malaria in Sumatera, Indonesia.** *J Infect Dis* 2013, **208:**1906-1913.
53. Jittamala P, Pukrittayakamee S, Ashley EA, Nosten F, Hanboonkunupakarn B, Lee SJ, Thana P, Chairat K, Blessborn D, Panapipat S, et al: **Pharmacokinetic interactions between primaquine and pyronaridine-artesunate in healthy adult Thai subjects.** *Antimicrob Agents Chemother* 2015, **59:**505-513.
